# Supplementary material for: Enhancing care quality and accessibility through digital technology-supported decentralisation of hypertension and diabetes management: a proof-of-concept study in rural Bangladesh
Source: BMJ Open. 2023 Nov 19;13(11):e073743. doi: 10.1136/bmjopen-2023-073743 (PMC10660961; doi:10.1136/bmjopen-2023-073743)
Supplement: Supplementary data [file bmjopen-2023-073743supp001.pdf]

Supplementary Materials

TITLE: Enhancing Care Quality and Accessibility through Digital Technology-Supported Decentralization of Hypertension and Diabetes Management: A Proof-of-Concept Study in Rural Bangladesh

Xie W, *et al. BMJ Open* 2023; 13:e073743. doi: 10.1136/bmjopen-2023-073743

The development and deployment of a digital platform for primary care hypertension and diabetes care coordination in Bangladesh

Background

The long-term nature of hypertension and diabetes (and other noncommunicable diseases), which require regular engagement and health monitoring for improved patient outcomes, make it critical for healthcare personnel in primary health care (PHC) teams to be collaborative and coordinated to ensure efficient and effective care delivery.[1,2] Digital platforms have the potential to enable greater collaboration and coordination that will be helpful in addressing many of the barriers to effective NCD management within the PHC system.[3] Digital platforms can help the health care system to document the risks and health states of the population, and to prioritize delivery of care to those at greatest need. Through more efficient data capture and sharing, guideline-based decision support, generation of individualized care plans and management of care delivery actions, the implementation of digital platforms support the move towards coordinated, evidence-based and data-driven NCD management for populations served by the PHC team.[4]

Identification of gaps in usual care

The first step in identifying the components of the intervention was to assess the gaps in usual care through systematic reviews of literature on epidemiological studies, NCD service delivery in LMIC and in Bangladesh, and through discussion with experts and stake holders. Multiple meetings with primary care physicians, NCD nurses, community health workers, and local health authorities were organized to understand the current care pathway, and to identify gaps in usual care. Consultations with domain experts, including cardiologists, nutritionists, epidemiologists, statisticians, data scientists, pharmacists, were conducted. The team mapped out the journey of patients under current model of government service delivery and a model of decentralized care with the involvement of the most peripheral primary care facilities and community-based interventions by community health workers (CHWs).

Through these processes, a few key gaps were identified include 1) using of paper-based health records limits exchange of critical information, 2) adherence to national guidelines and treatment protocols is inconsistent among health care providers, 3) patient management of medication adherence and follow-up are irregular, 4) lack of coordination in service delivery which involves a wide range of settings and personnel, which included subdistrict level primary care facilities, community clinics - the most peripheral primary health care facilities, and doctors, nurses, community healthcare providers (CHCPs), and CHWs.

Overview of the development processes

The digital platform was developed using the user-centred design (UCD), an iterative software development methodology in which the development team focuses on the users and their needs in each phase of the development process.[5] A multi-disciplinary technology development team consisting of domain experts, software engineers and user design researchers utilized UCD in the development of the Coordinate platform that with each iteration, produced a usable version of the software that was increasingly aligned with the needs of its users. The process was completed when a sufficiently developed version of the software was produced that could be deployed for use in the study.

One of the core value propositions of Coordinate platform is to use it in the community. In rural areas of Bangladesh, internet connectivity is a major problem, and many online platforms need to adopt offline functionality to operate in rural areas. Hence, offline functionality of the Coordinate platform was designed with different use cases and functionalities.

The Android OS was chosen as the operating system platform due to the greater flexibility it offers in app development, testing and deployment, along with users' familiarity due to widespread usage in South Asia and the availability of low-cost tablets. The platform is underpinned by an administrator web-dashboard application built using Laravel, an open-source PHP framework and connected to a database built using PostgresSQL, open-source relational database management system. The platform was development through four iterative loops from June 2020 to December 2021. The platform was designed following international standards of interoperability, with clearly documented application programming interfaces. The digital platform resources are open-source and can be readily utilized by the global health community.

Content and functionalities

Based on the results from the need assessment, the digital platform was built to support the needs of various health service providers, including new users (CHCPs, CHWs) who would be trained to provided care in a decentralized care model. Accordingly, a core set of functionalities was developed to support the coordination of patient care through enhanced data capture and sharing, guideline-based decision support, generation of individualized care plans and management of care delivery actions (**Supplementary Figure B**).

The system consists of a tablet-based application for community health workers to register participants at risk, administer in-field questionnaire, deliver care plan, and refer. CHCPs at community clinics have access to information entered by CHWs. CHCPs will then perform a simplified structured clinical evaluation, including medical

history, anthropometrics, blood pressure, and blood glucose. Based on the information obtained, the CHCPs conduct lifestyle education for high-risk behaviours with assistance from built-in point-of-care support and referred the participant with elevated risks for further confirmation at subdistrict NCD clinic. The participant's electronic health record will be updated to reflect the additional information obtained by CHCPs and be available to nurses and doctors at NCD clinic.

Once a participant approaches NCD clinic, nurses perform additional examinations and update patient data. The platform generates CVD risk scores based on WHO cardiovascular disease risk non-laboratory-based charts (South Asia) and have all pertinent data for physicians to make treatment decision and management plan, which are then incorporated into patient profile. The patient list is then generated and continuously updated for CHWs in the community and CHCPs at community clinics to follow up with routine examination, medication refill, and counselling. Some of the user interfaces were illustrated in **Supplementary Figure C**.

The doctors and nurses have access to a web-based central administration dashboard which allow them to track patient treatment progress and referrals, and to add/remove users (**Supplementary Figure D**). A demo video overview of the platform is available at https://youtu.be/nXmlX0_RZVY.

Conclusions

The digital platform is among the first to support a coordinated primary care delivery for hypertension and diabetes management across all levels of primary care providers and community health workers. Digital tools can be invaluable in strengthening health systems, although they need to be an integral part of an intervention package to exert effects within specific health system contexts.[6] Multifaceted intervention is often needed to maximize the impact of a digital tool.[2] Following international standards of interoperability throughout the designing phrase, the digital platform resources are open-source and can be readily utilized by the global health community.

In the next steps, we are planning to add training and education of primary care providers and patient self-management components to the platform. Specifically, we planned to develop online training program in collaboration with domain experts and key stakeholders. Digital training modules will be developed for use by PHC teams, tailored to user role and setting. These highly scalable modules for development of NCD care skills may reduce the need for in-person sessions. In addition, to empower community users, we planned to develop a user-facing module that gives participants access to their health information, personalised care plans, and evidence-based self-management content. Finally, we will refine and extend on the open-source decision-support algorithms in our platform to simplify the process of tailoring to local context, and to

enhance formulation of adaptive longitudinal care plans. This includes taking into account additional parameters in decision-making to strengthen care plan recommendations and drive effective disease management.

References

1. Kabir, A., Karim, M. N., Islam, R. M., Romero, L. & Billah, B. Health system readiness for non-communicable diseases at the primary care level: a systematic review. BMJ open 12, e060387 (2022).
2. Kruk, M. E., Nigenda, G. & Knaul, F. M. Redesigning primary care to tackle the global epidemic of noncommunicable disease. American journal of public health 105, 431-437 (2015).
3. Labrique, A. B. et al. Best practices in scaling digital health in low and middle income countries. Globalization and health 14, 1-8 (2018).
4. Mechael, P. et al. Barriers and gaps affecting mHealth in low and middle income countries: Policy white paper. (2010).
5. Bazzano AN, Martin J, Hicks E, Faughnan M, Murphy L. Human-centred design in global health: A scoping review of applications and contexts. Virgili G, editor. PLOS ONE 2017 Nov 1;12(11):e0186744.
6. Praveen, D., et al. (2014). "SMARTHealth India: development and field evaluation of a mobile clinical decision support system for cardiovascular diseases in rural India." JMIR mHealth and uHealth **2**(4): e3568.

Supplementary Figure A. Bangladesh national protocol for integrated management of hypertension, diabetes and cholesterol using a total cardiovascular risk approach

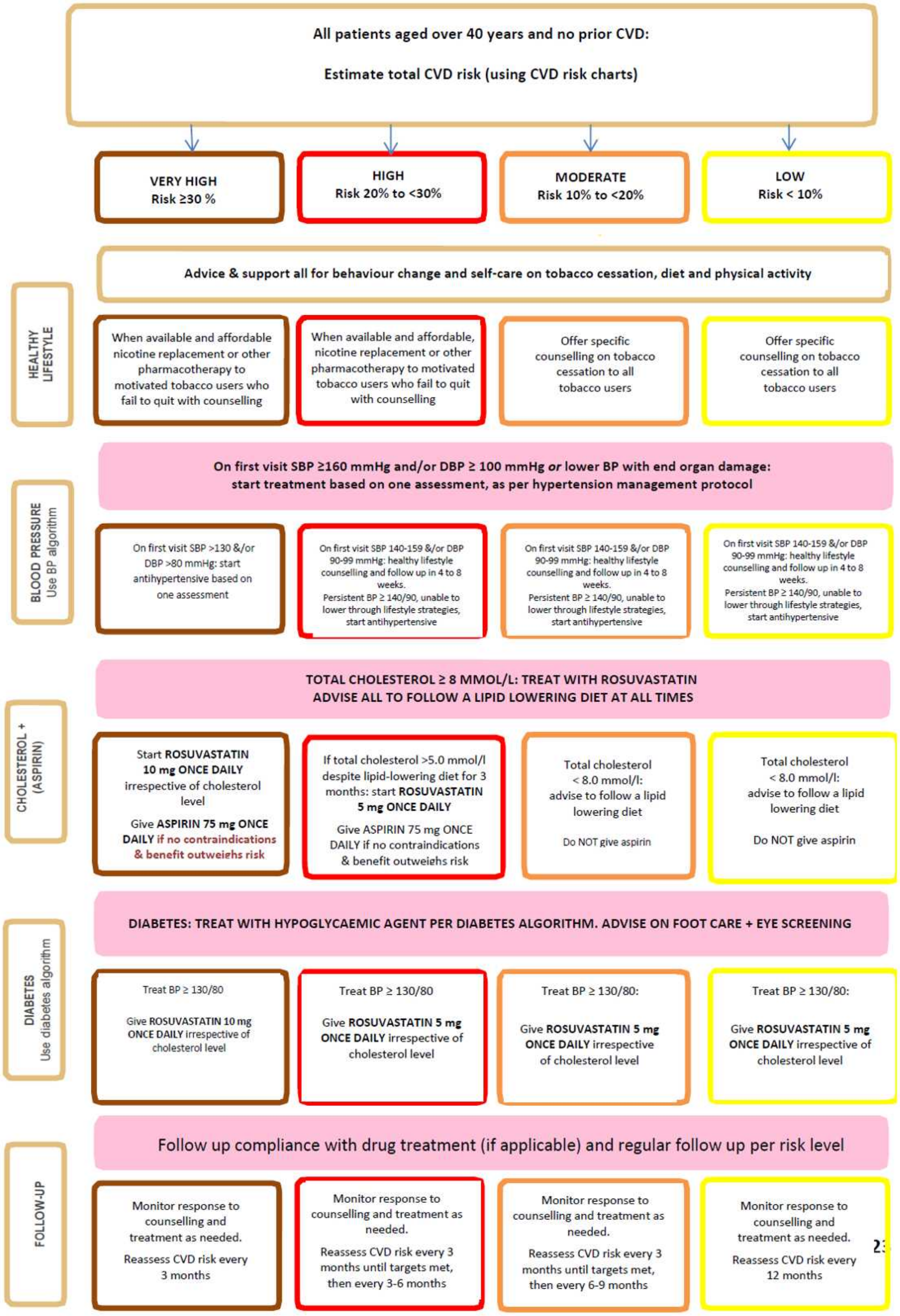

Xie W, *et al. BMJ Open* 2023; 13:e073743. doi: 10.1136/bmjopen-2023-073743

Bangladesh national protocol for management of hypertension at primary health care settings

Decision to start antihypertensive medication taken based on estimation of total cardiovascular disease (CVD) risk of patients aged ≥ 40 years [a, b]

Step 1: **START AMLODIPINE 5 mg ONCE DAILY** [c]

Step 2: Review after 1 month, if treatment target not met [d,e]
ADD LOSARTAN 50 mg ONCE DAILY

Step 3: Review after 1 month, if treatment target not met [d]
ADD HYDROCHLOROTHIAZIDE 12.5 mg ONCE DAILY

Step 4: Review after 1 month, if treatment target not met [d]
REFER TO A SPECIALIST

THIS PROTOCOL SHOULD NOT BE USED FOR PATIENTS WHO *ARE* PREGNANT

IF PREGNANT, REFER TO SPECIALIST [f]

DO NOT PRESCRIBE LOSARTAN (OR ANY ACE-I OR ARBs) to women who *may* become pregnant.

For women of childbearing age who *may* become pregnant:

- Start amlodipine 2.5 – 5 mg once daily
- Review after one month. If treatment target not met, increase amlodipine to 10 mg once daily [d]
- Review after one month, if treatment target not met, REFER [d]

a. Refer to 'Protocol for Integrated Management of Hypertension, Diabetes and Cholesterol using **'Total Cardiovascular Risk Approach'** for **making decision** to treat hypertension in patients **aged ≥ 40 years.**
b. This protocol may also be used to treat patients below 40 years of age when treatment for hypertension is indicated.
c. Consider Amlodipine 2.5 mg as starting dose for patients aged ≥ 60 years and/ or patients with low body weight (BMI ≤ 18.5).
d. Check that patient has been taking drugs regularly and correctly, prior to increasing dose.
e. Before initiating and several weeks after starting losartan, check serum creatinine and serum potassium. **Avoid losartan for women of childbearing age who may become pregnant.**
f. If treatment for hypertension is to be initiated for pregnant women before referring her to specialist, consider methyldopa 125 – 250 mg twice daily

Treatment target based on CVD risk based management	
Most patients	≤ 140/90 mmHg
Having comorbidity: diabetes, ischaemic heart disease, stroke, chronic kidney disease	≤ 130/80 mmHg
High CVD Risk >30% (as per CVD risk prediction chart)	≤ 130/80 mmHg

LILFESTYLE MODIFICATION ADVICE FOR ALL PATIENTS

- Stop all tobacco use, avoid secondhand tobacco smoke.
- Stop taking alcohol.
- Increase physical activity to equivalent of brisk walk 150 minutes per week.
- If overweight, lose weight.
- Eat heart-healthy diet:
  - Reduce dietary salt intake.
  - Eat ⩾ 5 servings of vegetables/fruit per day.
  - Use healthy oils, such as sesame (Til), olive, safflower, sunflower.
  - Eat nuts, peas, whole grains and foods rich in potassium like spinach, watermelon, yogurt and banana.
  - Limit red meat to once or twice a week at most.
  - Eat fish or other food rich in omega 3 fatty acids at least twice a week.
  - Avoid added sugar from sweets, cakes, cookies, fizzy drinks, sugar sweetened beverages

Xie W, *et al. BMJ Open* 2023; 13:e073743. doi: 10.1136/bmjopen-2023-073743

Bangladesh National Protocol for Management of Type 2 Diabetes at Primary Health Care Level

Test patients who have symptoms of diabetes. Test adults who are 40+ years old. Test pregnant women during antenatal checkup

Diabetes diagnosed as per diagnostic criteria

Advise on diet, physical exercise and healthy lifestyle

Goal for glycaemic control: Fasting plasma glucose 4.4 – 7.2 mmol/L; Postprandial plasma glucose ≤ 10 mmol/L; Glycated haemoglobin ≤ 7%

Fasting Plasma Glucose (FPG) 7.0 – 10.0 mmol/L, or<br>Random Plasma Glucose (RPG) 11.1 – 12.0 mmol/L, or<br>Glycated Haemoglobin (A1C) 6.5 - 8.5%	FPG 10.1 – 14 mmol/L, or<br>RPG 12.1 – 18 mmol/L, or<br>A1C 8.6 – 10.0%	FPG > 14 mmol/L, or<br>RPG > 18 mmol/L, or<br>A1C > 10.0%
Counsel on diet and physical activity<br>↓<br>Review in 1 month.<br>If glycaemic goal not achieved:<br>Initiate with METFORMIN 250 – 500 mg once daily. *<br>Counsel on diet, physical activity and adherence to treatment at all visits.<br>↓<br>Review in 1 month.<br>If goal not achieved, increase dose of METFORMIN to 500 mg twice daily<br>↓<br>Review in 1 month.<br>If goal not achieved, increase dose of METFORMIN to 1000 mg twice daily<br>↓<br>Review in 3 month.<br>If goal not achieved, *ADD* GLICLAZIDE, 40 mg once a day<br>Counsel on hypoglycemia at all subsequent visits<br>↓<br>Review in one month.<br>If goal not achieved, increase dose of GLICLAZIDE by 40 mg every month till goal is achieved/ maximum dose of 80 mg twice daily is reached<br>↓	Start METFORMIN 500 mg twice daily +<br>GLICLAZIDE 40 mg twice daily. *<br>Counsel on diet, physical activity and adherence to treatment at all visits<br>↓<br>Review in 1 month.<br>If glycaemic goal not achieved, increase dose of METFORMIN to 1000 mg twice daily<br>↓<br>Review in 1 month.<br>If goal not achieved, increase dose of GLICLAZIDE by 40 mg every month, till 80 mg twice daily<br>↓	REFER to higher-level of care for starting insulin

Review in 3 months.
If goal not achieved, despite adherence to medication, healthy diet, and physical activity, REFER to higher-level of care for starting insulin

* If serum creatinine is > 1.5 mg/ dl or there is contraindication/ intolerance to Metformin, start with Gliclazide 40 mg daily. Review and increase dose by 40 mg every month till goal is achieved/ maximum recommended dose is reached.

Xie W, *et al. BMJ Open* 2023; 13:e073743. doi: 10.1136/bmjopen-2023-073743

Supplementary Figure B. digital platform designed to support multiple healthcare personnel roles along the care pathway

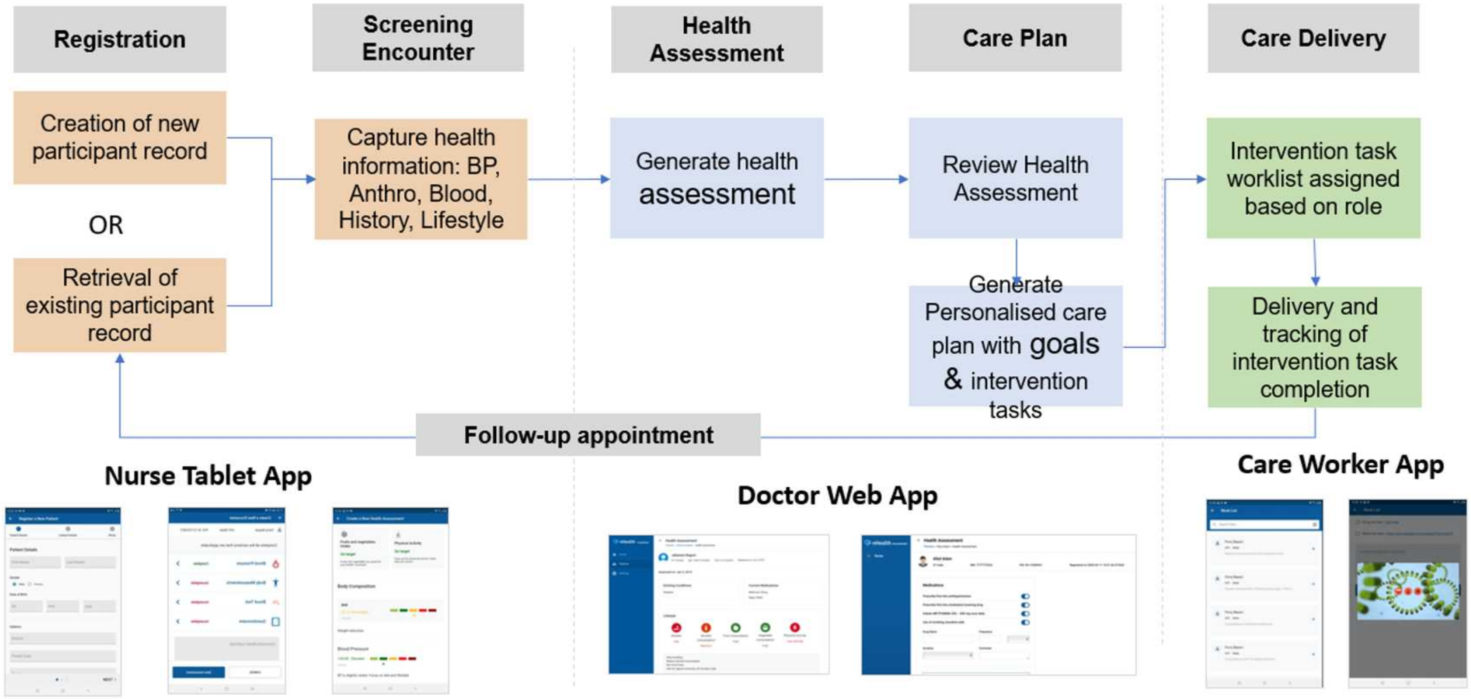

Xie W, *et al. BMJ Open* 2023; 13:e073743. doi: 10.1136/bmjopen-2023-073743

Supplementary Figure C. User interfaces for (A) registration, (B) patient record, (C) worklist, (D) referral, (E) care plan action, (F) counselling support

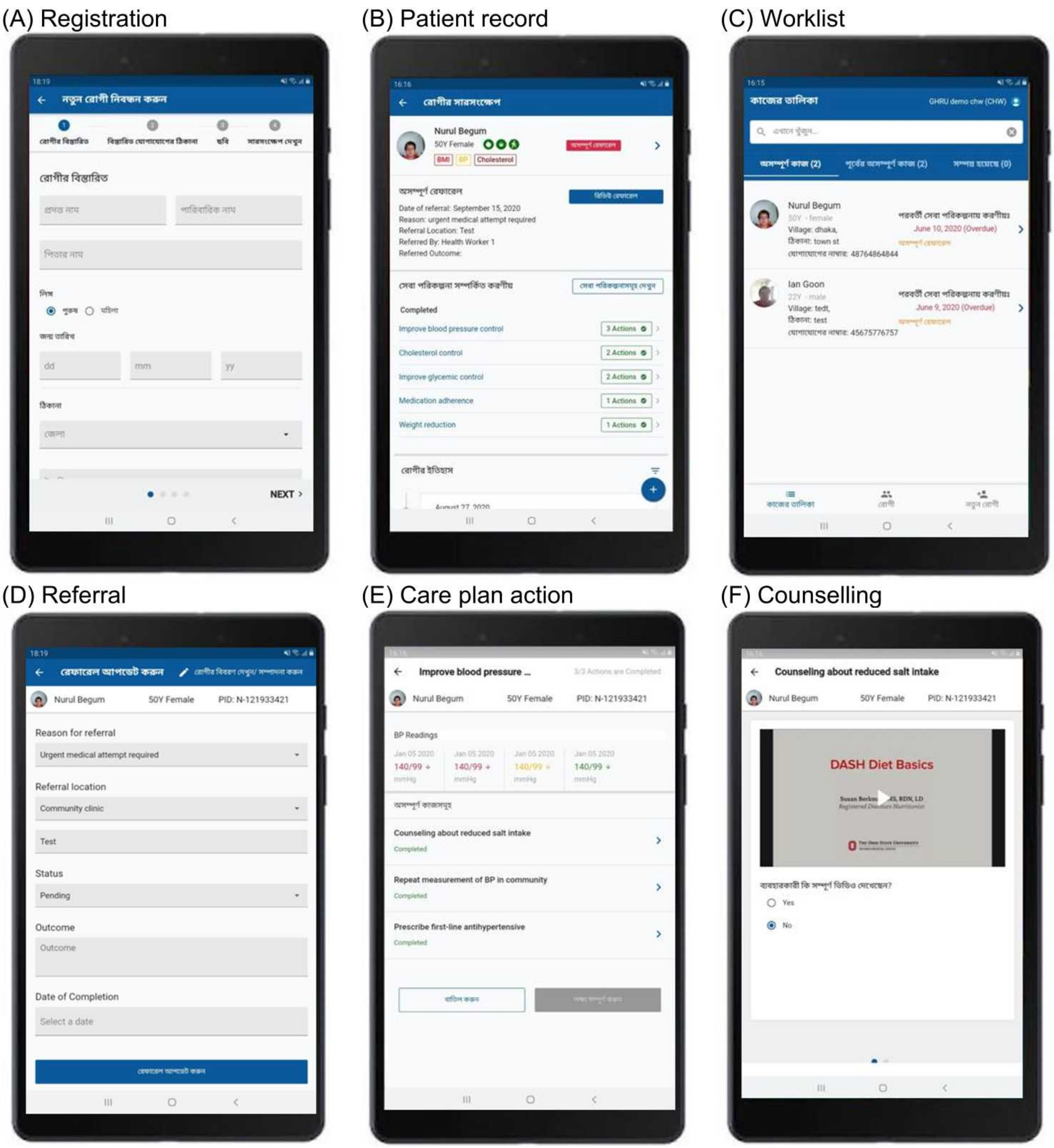

Xie W, *et al. BMJ Open* 2023; 13:e073743. doi: 10.1136/bmjopen-2023-073743

Supplementary Figure D. Central administration dashboard

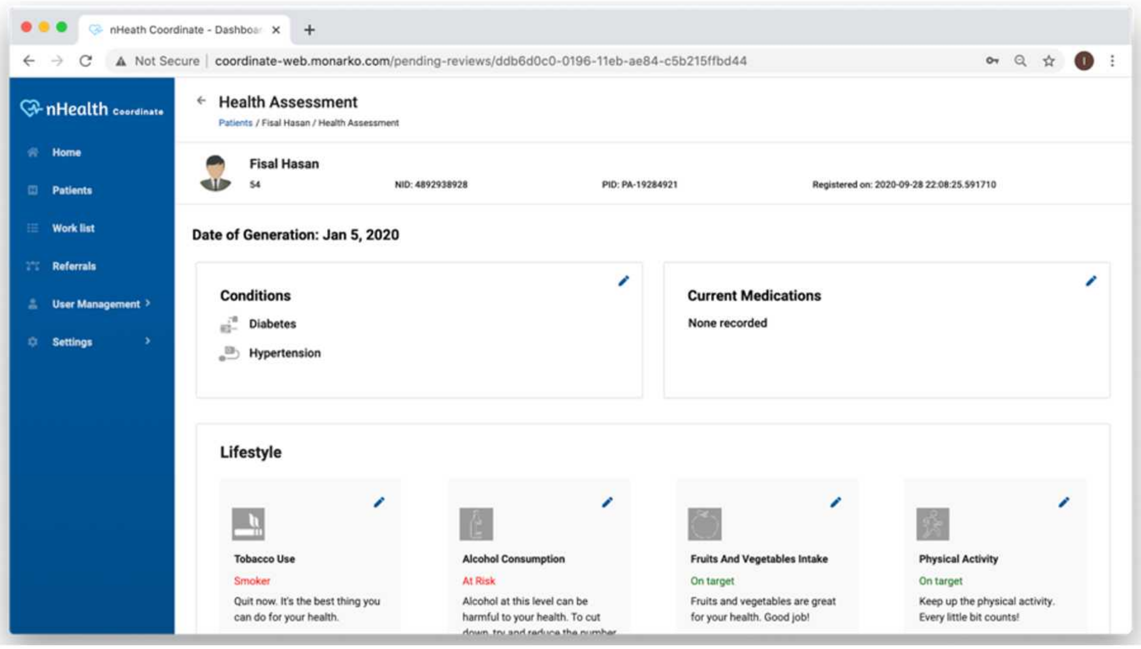

Supplementary Figure E. Consort diagram of the numbers of patients enrolled into care

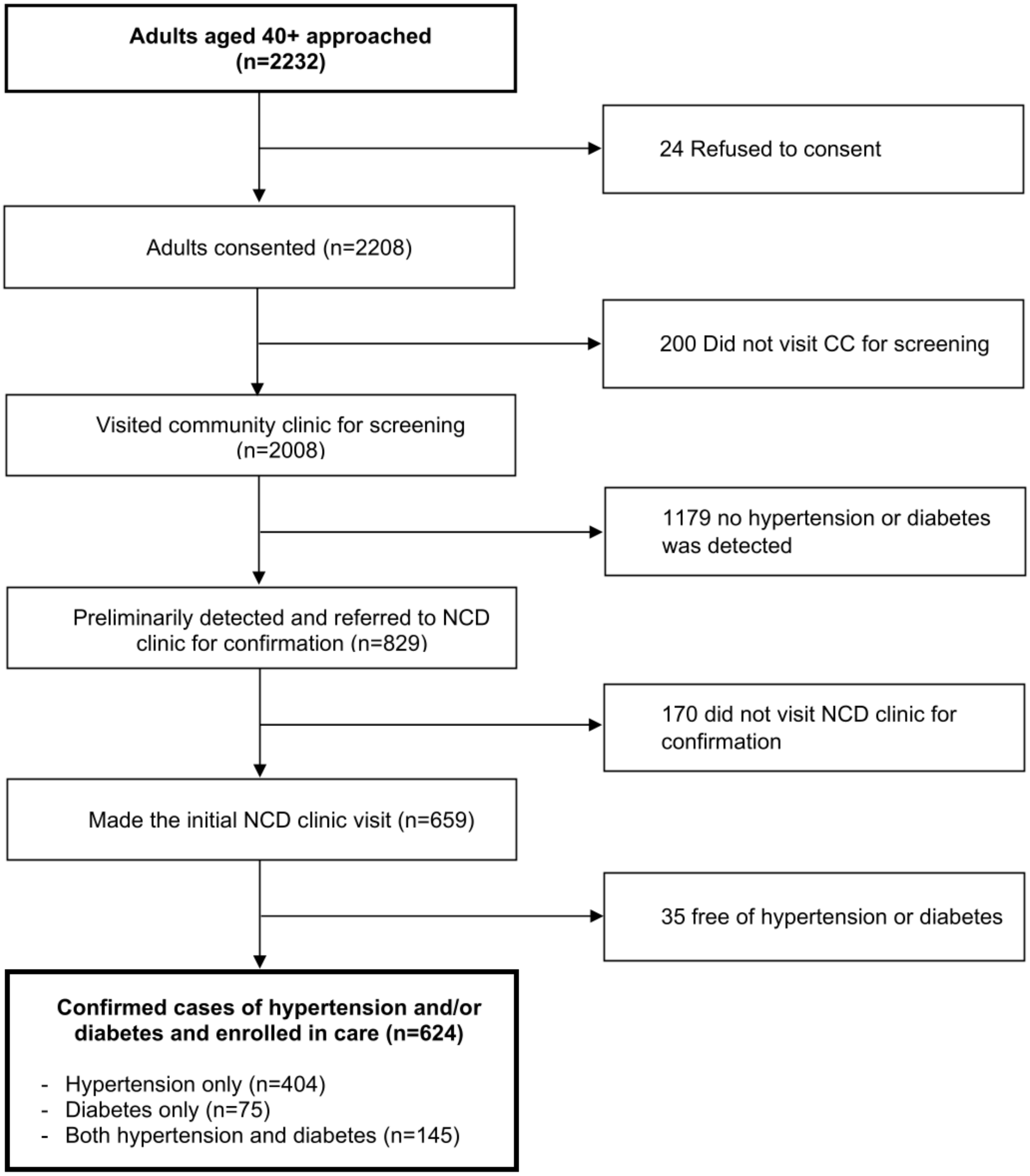

Xie W, *et al. BMJ Open* 2023; 13:e073743. doi: 10.1136/bmjopen-2023-073743

Supplementary Figure F. Change in mean blood pressure from baseline to follow-up visits *

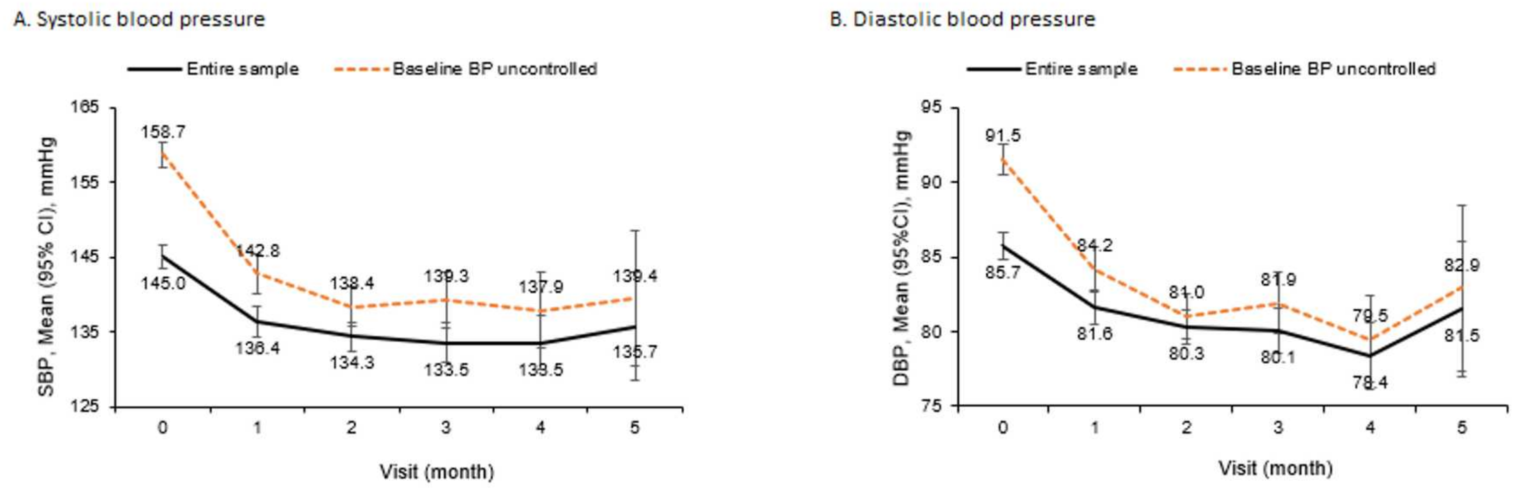

A. Systolic blood pressure

Entire sample	549	314	206	135	68	20
Uncontrolled	331	193	121	79	43	15

B. Diastolic blood pressure

Entire sample	549	314	206	135	68	20
Uncontrolled	220	146	101	71	36	17

* BP, blood pressure; SBP, systolic blood pressure; DBP, diastolic blood pressure
Results adjusted age and sex.
Uncontrolled blood pressure at baseline was defined as systolic blood pressure ≥140 mmHg or diastolic blood pressure ≥90 mmHg.

Xie W, *et al. BMJ Open* 2023; 13:e073743. doi: 10.1136/bmjopen-2023-073743

Supplementary Figure G. Number of patient-visits per month occurred in NCD clinic and community clinic by types of tasks

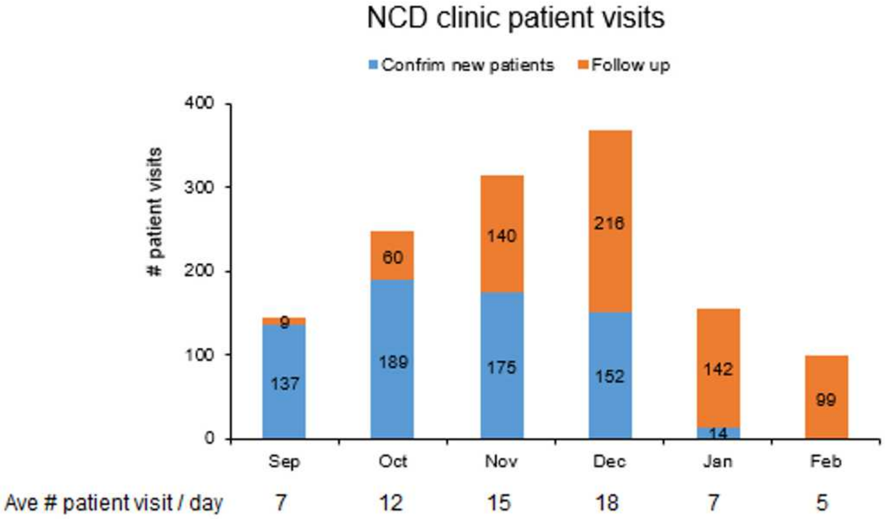

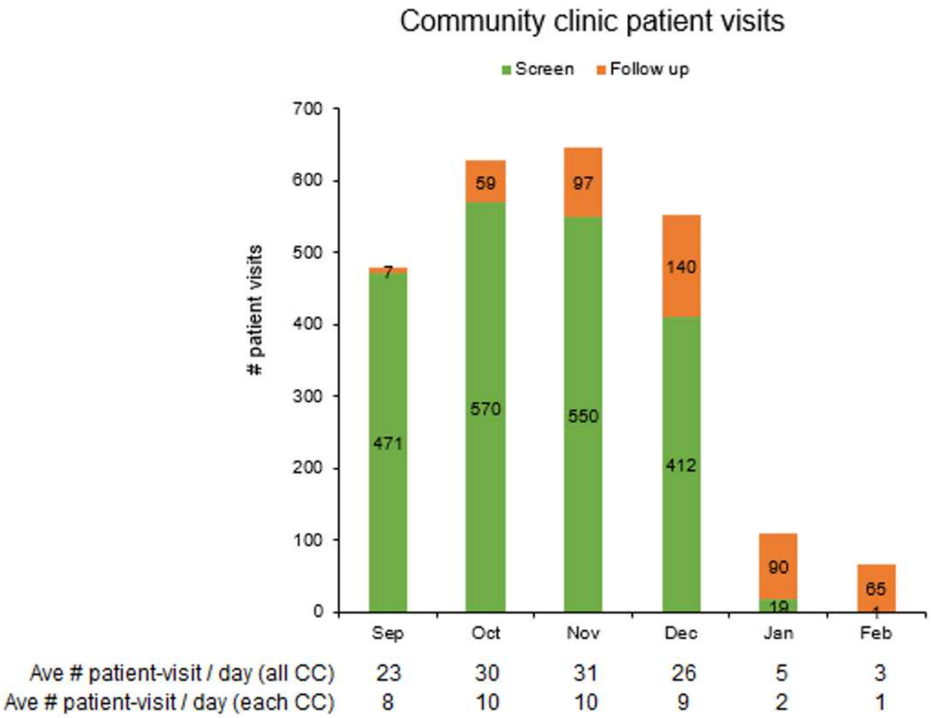

Supplementary Table A. Change in mean blood pressure from baseline to follow-up visits

**Visit** *	Systolic blood pressure		Diastolic blood pressure	
	Entire sample	Baseline uncontrolled cases †	Entire sample	Baseline uncontrolled cases †
1 month	-8.0 (-10.2, -5.9)	-15.2 (-17.8, -12.5)	-3.9 (-5.0, -2.7)	-7.2 (-8.7, -5.8)
2 months	-9.7 (-12.2, -7.3)	-19.7 (-22.7, -16.8)	-4.9 (-6.3, -3.6)	-10.2 (-11.9, -8.5)
3 months	-10.6 (-13.6, -7.2)	-18.8 (-22.8, -14.8)	-5.3 (-6.9, -3.6)	-9.5 (-11.7, -7.3)
4 months	-10.6 (-14.4, -6.8)	-19.9 (-24.8, -15.0)	-7.0 (-9.3, -4.6)	-11.8 (-14.7, -8.8)
5 months	-8.5 (-16.0, -0.8)	-18.3 (-27.5, -9.1)	-3.9 (-8.5, 0.8)	-8.3 (-14.1, -2.5)
Constant	126.7 (119.5, 134.1)	160.5 (150.7, 170.4)	77.2 (72.5, 81.8)	93.4 (86.5, 100.2)
N	549	331	549	331

* All models adjusted for age (40-49, 50-59, 60-69, 70-100), sex (male, female), education (no formal education, primary or lower, secondary or higher), religion (Islam, Hindu), BMI category (under, normal, overweight or obese), community clinics (Khorakhai, Manmathapur, Jahanabad).

† Baseline uncontrolled cases were defined as systolic blood pressure ≥ 140 mmHg or diastolic blood pressure ≥ 90 mmHg

Xie W, *et al. BMJ Open* 2023; 13:e073743. doi: 10.1136/bmjopen-2023-073743

Supplementary Table B. Factors associated with loss to follow-up

	LFU in the first visit	LFU over entire period
**Predictor**	IRR‡ (95% CI)	IRR‡ (95% CI)
Community clinic		
Khorakhai	1.00	1.00
Jahanabad	1.45 (1.02, 2.04)	1.21 (0.84, 1.74)
Manmathapur	1.58 (1.13, 2.22)	1.70 (1.22, 2.38)
Chronic conditions		
Both	1.00	1.00
Hypertension only	1.89 (1.31, 2.72)	1.85 (1.27, 2.70)
Diabetes only	1.27 (0.76, 2.14)	1.60 (0.96, 2.66)
Hypertension condition		
Known case, uncontrolled	1.00	1.00
Known case, controlled	1.42 (0.98, 2.06)	1.55 (1.07, 2.26)
New case	1.70 (1.20, 2.42)	1.48 (1.01, 2.15)
Religion		
Hindu	1.00	1.00
Islam	0.45 (0.32, 0.63)	0.85 (0.54, 1.32)

LFU, loss to follow up; IRR, incident rate ratio
Loss to follow-up over the entire study period is defined as no consultation recorded for two months or more.
IRRs derived from separate discrete time Poisson models controlling for age and sex, a logarithm exposure time were incorporated; only variables showing statistically significant associations were presented.

Supplementary Table C. Change in mean blood pressure from baseline to follow-up visits - sensitivity check for attrition and composition *

Visit	Original results	IPW weighting ‡	Restricting to patients with 3+ follow-up visits
**Uncontrolled blood pressure §**			
1 month	0.63 (0.55, 0.72)	0.65 (0.57, 0.75)	0.63 (0.51, 0.78)
2 months	0.48 (0.39, 0.60)	0.49 (0.39, 0.61)	0.48 (0.36, 0.64)
3 months	0.44 (0.33, 0.57)	0.43 (0.32, 0.57)	0.44 (0.33, 0.59)
4 months	0.49 (0.35, 0.69)	0.48 (0.34, 0.68)	0.50 (0.35, 0.71)
5 months	0.64 (0.32, 1.25)	0.60 (0.29, 1.24)	0.65 (0.33, 1.27)
N	549	549	135
**Uncontrolled blood glucose ¶**			
1 month	0.69 (0.58, 0.82)	0.71 (0.59, 0.85)	0.63 (0.49, 0.80)
2 months	0.66 (0.54, 0.81)	0.67 (0.53, 0.83)	0.59 (0.45, 0.77)
3 months	0.60 (0.45, 0.78)	0.62 (0.47, 0.82)	0.55 (0.41, 0.72)
4 months	0.54 (0.36, 0.80)	0.56 (0.37, 0.85)	0.47 (0.30, 0.72)
5 months	0.44 (0.20, 0.96)	0.42 (0.18, 0.95)	0.40 (0.18, 0.89)
N	220	220	71

* All models adjusted for age (40-49, 50-59, 60-69, 70-100), sex (male, female), education (no formal education, primary or lower, secondary or higher), religion (Islam, Hindu), BMI category (under, normal, overweight or obese), community clinics (Khorakhai, Manmathapur, Jahanabad).

‡ The inverse probability weighting approach weighted the analysis by the inverse of the predicted probability of dropping out from the study. A conditional logit model with the same set of covariates described above were used to predict the probabilities.

§ Uncontrolled blood pressure was defined as systolic blood pressure ≥ 140 mmHg or diastolic blood pressure ≥ 90 mmHg

¶ Uncontrolled blood glucose was defined as fasting plasma glucose ≥ 7.0 mmol/L or random plasma glucose ≥ 11.1 mmol/L

Xie W, *et al. BMJ Open* 2023; 13:e073743. doi: 10.1136/bmjopen-2023-073743
