# Supplementary material for: Enhancing care quality and accessibility through digital technology-supported decentralisation of hypertension and diabetes management: a proof-of-concept study in rural Bangladesh
Source: BMJ Open. 2023 Nov 19;13(11):e073743. doi: 10.1136/bmjopen-2023-073743 (PMC10660961; doi:10.1136/bmjopen-2023-073743)
Supplement: Author's manuscript [file bmjopen-2023-073743.draft_revisions.pdf]

BMJ Open

BMJ Open is committed to open peer review. As part of this commitment we make the peer review history of every article we publish publicly available.

When an article is published we post the peer reviewers' comments and the authors' responses online. We also post the versions of the paper that were used during peer review. These are the versions that the peer review comments apply to.

The versions of the paper that follow are the versions that were submitted during the peer review process. They are not the versions of record or the final published versions. They should not be cited or distributed as the published version of this manuscript.

BMJ Open is an open access journal and the full, final, typeset and author-corrected version of record of the manuscript is available on our site with no access controls, subscription charges or pay-per-view fees (http://bmjopen.bmj.com).

If you have any questions on BMJ Open's open peer review process please email info.bmjopen@bmj.com

BMJ Open

Enhancing Care Quality and Accessibility through Digital Technology-Supported Decentralization of Hypertension and Diabetes Management: A Proof-of-Concept Study in Rural Bangladesh

Journal:	*BMJ Open*
Manuscript ID	bmjopen-2023-073743
Article Type:	Original research
Date Submitted by the Author:	17-Mar-2023

Complete List of Authors:	Xie, Wubin; Nanyang Technological University<br>Paul, Rina Rani; BRAC University James P Grant School of Public Health, Centre for Non-communicable Diseases and Nutrition<br>Goon, Ian; UNSW, Tyree Foundation Institute of Health Engineering; Baker Heart and Diabetes Institute<br>Anan, Aysha; BRAC University James P Grant School of Public Health, Centre for Non-communicable Diseases and Nutrition<br>Rahim, Aminur; Sprightly Pte Ltd<br>Hossain, Md Mokbul; BRAC University, Centre for Non-communicable Diseases and Nutrition<br>Hersch, Fred; Google Health<br>Oldenburg, B; Baker Heart and Diabetes Institute<br>Chambers, John; Nanyang Technological University; Imperial College London, Department of Epidemiology and Biostatistics<br>Mridha, Malay Kanti; BRAC University James P Grant School of Public Health, Centre for Non-communicable Diseases and Nutrition

Keywords:	Hypertension < CARDIOLOGY, DIABETES & ENDOCRINOLOGY, Health Services Accessibility

SCHOLARONE™
Manuscripts

I, the Submitting Author has the right to grant and does grant on behalf of all authors of the Work (as defined in the below author licence), an exclusive licence and/or a non-exclusive licence for contributions from authors who are: i) UK Crown employees; ii) where BMJ has agreed a CC-BY licence shall apply, and/or iii) in accordance with the terms applicable for US Federal Government officers or employees acting as part of their official duties; on a worldwide, perpetual, irrevocable, royalty-free basis to BMJ Publishing Group Ltd ("BMJ") its licensees and where the relevant Journal is co-owned by BMJ to the co-owners of the Journal, to publish the Work in this journal and any other BMJ products and to exploit all rights, as set out in our licence.

The Submitting Author accepts and understands that any supply made under these terms is made by BMJ to the Submitting Author unless you are acting as an employee on behalf of your employer or a postgraduate student of an affiliated institution which is paying any applicable article publishing charge ("APC") for Open Access articles. Where the Submitting Author wishes to make the Work available on an Open Access basis (and intends to pay the relevant APC), the terms of reuse of such Open Access shall be governed by a Creative Commons licence – details of these licences and which Creative Commons licence will apply to this Work are set out in our licence referred to above.

Other than as permitted in any relevant BMJ Author's Self Archiving Policies, I confirm this Work has not been accepted for publication elsewhere, is not being considered for publication elsewhere and does not duplicate material already published. I confirm all authors consent to publication of this Work and authorise the granting of this licence.

For peer review only

Enhancing Care Quality and Accessibility through Digital Technology-Supported Decentralization of Hypertension and Diabetes Management: A Proof-of-Concept Study in Rural Bangladesh

Wubin Xie;[1] Rina Rani Paul; [2] Ian Goon;[3,4] Aysha Anan;[2] Aminur Rahim;[4] Md Mokbul Hossain;[2] Fred Hersch;[5] Brian Oldenburg;[4,7] John Chambers;[1,8*] Malay Kanti Mridha[2*]

[1] Lee Kong Chian School of Medicine, Nanyang Technological University, Singapore

[2] Centre for Non-communicable Diseases and Nutrition, BRAC James P Grant School of Public Health, Dhaka, Bangladesh

[3] Tyree Foundation Institute of Health Engineering, UNSW Sydney, NSW 2052, Australia

[4] Baker Heart and Diabetes Institute, Melbourne, Australia

[4] Sprightly Pte Ltd, Singapore

[5] Google Health, Palo Alto, CA, USA

[7] School of Psychology and Public Health, La Trobe University, Melbourne, Australia

[8] Department of Epidemiology and Biostatistics, School of Public Health, Imperial College London, London, UK

*** Corresponding authors:**

Prof John Chambers
E-mail: john.chambers@ntu.edu.sg
Tel: (65) 6592 3938

Prof Malay Kanti Mridha
E-mail: malay.mridha@bracu.ac.bd
Tel: (880) 2-48812213-18

Word count: 4170

For peer review only - http://bmjopen.bmj.com/site/about/guidelines.xhtml

Acknowledgements

This research was funded by the National Institute for Health Research (NIHR) (16/136/68) using UK aid from the UK Government to support global health research. The views expressed in this publication are those of the author(s) and not necessarily those of the NIHR or the UK Department of Health and Social Care.

The authors would like to thank Theresia Handayani Mina and Benjamin Lam Chih Chiang for their thoughtful comments on the manuscript.

Data availability statement

Available upon request.

Role of the funding source

The sponsor of the study was not involved in the study design, data collection, data analysis, data interpretation, or writing of the report. The authors had full access to all the data in the study and had final responsibility for the decision to submit for publication.

Contributors

MM, JC, IG, BO, RP, and FH contributed to the conception and design of the study. IG, AR, FH, MM, RP, MH, and AA worked on the development of the digital platform. RRP, AA, MM, and MH contributed to implementation of data collection and management. WX implemented analysis of data and wrote the first draft of the manuscript. All authors contributed to interpretation of data, and revision of the manuscript.

Declaration of interests

None.

For peer review only - http://bmjopen.bmj.com/site/about/guidelines.xhtml

Abstract

Objective The critical shortage of healthcare workers, particularly in rural areas, is a major barrier to quality care for noncommunicable disease (NCD) in low- and middle-income countries. In this proof-of-concept study, we aimed to test a decentralized model for integrated diabetes and hypertension management in rural Bangladesh to improve accessibility and quality of care.

Design and setting The key interventions comprised shifting screening, routine monitoring, and dispensing of medication refill from a doctor-managed subdistrict NCD clinic to non-physician health worker managed village level community clinics; a digital care coordination platform was developed for electronic health record, point-of-care support, referral, and routine patient follow-up. The study was conducted in Parbatipur subdistrict, Rangpur Division, Bangladesh.

Participants A total of 624 participants were enrolled in the study (mean[sd] age, 59.5 [12.0]; 65.1% female).

Outcomes Changes in blood pressure and blood glucose control, patient retention, and patient-visit volume at the NCD clinic and community clinics.

Results The proportion of patients with uncontrolled blood pressure reduced from 60% at baseline to 26% at the third month of follow-up, a 56% (IRR 0.44; 95% CI 0.33, 0.57) reduction after adjustment for covariates. The proportion of patients with uncontrolled blood glucose decreased from 74% to 43% at the third month of follow-up. Attrition rates immediately after baseline and during the entire study period were 29.1% and 36.2%, respectively.

For peer review only - http://bmjopen.bmj.com/site/about/guidelines.xhtml

For peer review only

Conclusion The study highlights the potential for involving lower-level primary care facilities and non-physician health workers to rapidly expand much needed services to the patients with hypertension and diabetes in Bangladesh, and in similar global settings. Further investigations are needed to evaluate the effectiveness of decentralized hypertension and diabetes care.

For peer review only

For peer review only - http://bmjopen.bmj.com/site/about/guidelines.xhtml

Strength and limitation of the study

- The study is the first to test a digital technology supported decentralized model for integrated diabetes and hypertension management in a resource-poor setting.
- Generated important preliminary effectiveness data on a decentralized model to rapidly expand primary care services to people with hypertension and diabetes.
- Enabled by digital tools, rich longitudinal data were collected to evaluate patient treatment outcome and retention.
- Duration of the study is short, and generalizability is limited by a local sample drawn from a subdistrict in northern Bangladesh.

For peer review only

Introduction

Cardiovascular disease (CVD) and type 2 diabetes are major causes of premature death and disability. An estimated 10.5% (or 536.6 million) adults are living with diabetes, CVD caused 18.6 million deaths worldwide in 2019.[1] Close to 80% of CVD deaths occurred in low- and middle-income countries (LMICs).[2,3] The number of people with hypertension, a highly prevalent, major risk factor for CVD, doubled from 1990 to 2019.[4] Complications of persistently elevated blood pressure and poor glycaemic control, such as heart disease, stroke, chronic kidney disease, blindness, pose enormous socioeconomic burdens on individuals, families, and healthcare systems, contributing to poverty, inequality and social instability.[5] Despite the availability of effective and inexpensive medications and other management options for diabetes and hypertension for many years, treatment and control rates of these conditions remain unacceptably low in LMICs, particularly in South Asia.[6,7] Recent estimates indicate that in South Asia, only 26% men and 37% women with hypertension were on treatment, and control rates were only 11% and 17% for men and women respectively.[4]

Traditionally designed to provide basic episodic care with an emphasis on maternal and child health and infectious diseases, primary healthcare systems in LMICs are under resourced and ill-prepared for the surging burden of CVD and other noncommunicable diseases (NCD).[8] The critical shortage of healthcare workers, particularly in rural areas, is a major barrier to quality care.[9] Most evidently in grassroot level facilities, primary care providers are untrained and unequipped to address primary and secondary prevention of NCD.[10,11] As a result, NCD care is poorly accessible with

For peer review only - http://bmjopen.bmj.com/site/about/guidelines.xhtml

primary care systems.[12] Long waiting time and high costs associated with travel and lost wages discourage people from seeking care from qualified providers.[13-16]

Although few examples exist for hypertension and diabetes care, decentralization with task shifting (sharing) in HIV care has been identified as a viable strategy to rapidly scale-up antiretroviral therapy in resource-limited settings.[17-19] In this model of care, stable patients are transferred through a 'down-referral' system from the doctor-managed clinic where they initiated care to non-physician health worker managed local primary health clinics for continued monitoring and treatment. Decentralized care improves geographic accessibility, reduces costs associated with care seeking, and enables the health system to serve a much larger patient population.[17] Local health service providers could monitor patients more effectively and efficiently, and to form a personal relationship that strengthens effective communication and counselling for treatment management.[20] People with HIV/AIDS managed locally are less likely to be lost to follow-up and more satisfied with care, compared with those managed in centralized clinics.[21]

Digital technologies offer the potential to improve access to and quality of healthcare in LMICs.[22] Digital tools have been tested in supporting quality improvement in hypertension and diabetes management in South Asian settings. Several pilot studies have documented the utility of digital tools to facilitate protocol-based algorithmic care through clinical decision support, and to promote continuity and coordination of patient visits across different healthcare settings, although findings are somewhat mixed.[23-25]

Little is known whether decentralizing routine hypertension and diabetes services to lower-level primary care facilities and non-physician health workers is beneficial for

For peer review only - http://bmjopen.bmj.com/site/about/guidelines.xhtml

enhancing care quality and accessibility in resource-poor settings, and how digital technology can be harnessed to support the processes. In the present proof-of-concept study, we aimed to generate preliminary data from testing the effectiveness of a digital technology supported decentralization of primary care diabetes and hypertension management in rural Bangladesh.

Methods

Context

Like many other LMICs, Bangladesh is experiencing rapid demographic and epidemiologic transitions. In the recent years, CVDs replaced infectious disease and maternal and neonatal conditions as the leading cause of death in Bangladesh.[26] The prevalence of hypertension and diabetes are high and continue to increase in recent years.[27] The 2017-2018 Bangladesh Demographic and Health Survey showed a 9.8% prevalence of diabetes and a 27.4% prevalence of hypertension among adult population.[28,29] Treatment and control rates remained low. Only 31.1% adults with hypertension and 28.2% adults with diabetes were on treatment, and merely 12% achieved blood pressure control and 7% had controlled diabetes.[29,30]

Bangladesh has a three-tiered (subdistrict, union, ward) primary healthcare system in rural areas (Figure 1). The subdistrict level Upazila Health Complexes, typically 50-bed hospitals, are at the top of the primary care system, serving a population of three to four hundred thousand. Community clinics are the most peripheral health care facilities and the first point of contact of public healthcare system to rural residents. With two rooms and drinking-water and lavatory facilities under a covered

For peer review only - http://bmjopen.bmj.com/site/about/guidelines.xhtml

For peer review only

waiting area, community clinics were designed to be accessible for more than 80% of rural population within less than 30-minute walking distance. Staffed by a Community Healthcare Provider with support from a few community health workers, community clinics have played critical roles in reducing malnutrition and child mortality, and improving maternal and child health.[31]

Since 2011, the Government of Bangladesh has promoted improved hypertension and diabetes care through national multisectoral actions, most notably through the establishment of dedicated noncommunicable disease related healthcare delivery points (“NCD Corners”) at subdistrict hospitals.[32] Although originally planned to involve lower-level primary care facilities in the delivery of NCD care, hypertension and diabetes care are largely unavailable at village level.[33]

Study settings

This proof-of-concept study was implemented in a newly initiated study NCD clinic at Parbatipur subdistrict, in collaboration with three community clinics at Khorakhai, Jahanabad, and Manmathapur. Parbatipur is a subdistrict of Dinajpur district, in Rangpur Division of northern Bangladesh (Supplementary Figure A). Parbatipur had a population of 365,103 in 2011. Public primary health care system in Parbatipur consists of one Upazila health complex (subdistrict hospital), 10 union health (sub)centres, and 34 community clinics.[34] With a maximum capacity of seeing 40-50 patients per day, the NCD Corner at the Parbatipur upazila health complex manages a few hundred diabetes and hypertension patients – a very small fraction of the estimated patient population with these conditions.

For peer review only - http://bmjopen.bmj.com/site/about/guidelines.xhtml

Study design

Decentralized hypertension and diabetes care comprised screening, routine monitoring, and dispensing of medication refill at village level community clinics and treatment initiation and titration at subdistrict level NCD clinic. Patient flow in this model of care included community health workers motivate adults aged 40 years and above to get screening for hypertension and diabetes at community clinics; once confirmed, patients received initial care at the study NCD clinic and subsequent follow-up at community clinics. At the follow-up visits, community healthcare providers provide basic physical examination, routine drug refill, ask for new symptoms, assess CVD risk, and counselling on medication adherence and healthy lifestyle. Patients were referred back to NCD clinic for medication titration and further evaluation if treatment target was not met or any urgent symptoms or conditions were recorded.

The study NCD clinic was staffed by one medical doctor and one paramedic. Both the doctor and the paramedic received a four-day training on hypertension and diabetes management following the package on essential NCD interventions of the World Health Organization. The paramedic had one year of post-graduate medical education. The doctor carried out physiological examination and provided diagnostic and treatment services according to the Bangladesh National Protocol for Management of Diabetes and Hypertension, adopted from the World Health Organization Package of essential non-communicable disease interventions.[35] The paramedic was responsible for taking medical history, measurement of blood pressure and blood glucose, delivery of health education, counselling, and medication disbursement.

For peer review only - http://bmjopen.bmj.com/site/about/guidelines.xhtml

One Community Healthcare Provider was employed to provide services in three community clinics (2 days per week in each community clinic). The Community Healthcare Provider was supported by three newly recruited NCD community health workers (one for each community clinic). The Community Healthcare Provider received a 1-year post-secondary education medical training. Community health workers were newly recruited from local communities with a minimum of secondary school education. The Community Healthcare Provider and the community health workers received a 7-day initial training on behavioural risk factors of hypertension and diabetes, anthropometry, lifestyle counselling and follow-up, and received ongoing regular (weekly to monthly) supervision from the study NCD clinic medical staff and the local study investigators.

The Community Healthcare Provider and community health workers were responsible for administering written informed consent, registering participants, referring adults for screening, follow-up, medication adherence, health education/counselling in the community. They were regularly supervised by the medical doctor from the study NCD clinic and by the study investigators. The study NCD clinic and community clinics were equipped with the necessary medical, pharmaceutical, and logistical supplies and equipment. Electronic health records were maintained by the study digital care coordination platform.

Patient screening and eligibility criteria

Adults aged 40 and above were approached at their households and registered and referred by community health workers to be screened at the community clinics.

For peer review only - http://bmjopen.bmj.com/site/about/guidelines.xhtml

Those with a history of hypertension or diabetes, and who screened positive for hypertension or diabetes were referred to the study NCD clinic for further evaluation. The WHO non-laboratory-based CVD risk scores were calculated.[35] Existing and new patients with hypertension and/or diabetes were enrolled. Diabetes was diagnosed if any of the following criteria was met: 1) participant reported taking medications for diabetes; 2) fasting plasma glucose (FPG) ≥ 7.0 mmol/L or random plasma glucose (RPG) ≥ 11.1 mmol/L with symptom of diabetes at one visit; 3) FPG ≥ 7.0 mmol/L or RPG ≥ 11.1 mmol/L at two separate visits, at least one day apart. Hypertension was diagnosed if any one of the following criteria was met: 1) participant reported taking medications for hypertension; 2) average systolic blood pressure (SBP) ≥160 mmHg and/or diastolic blood pressure (DBP) ≥100 mmHg on a single visit; 3) average SBP ≥130 mmHg and/or DBP ≥80 mmHg on a single visit and participant had a high risk of CVD (10-year CVD risk score ≥ 0.3) or participant reported a diagnosis of diabetes; 4) average SBP ≥140 mmHg and/or DBP ≥90 mmHg on two separate visit, at least one day apart. The diagnostic criteria were consistent with the Bangladesh National Protocol for Management of Diabetes and Hypertension.

Pregnant women and patients with life-threatening conditions were excluded from the study. Patients with very high blood pressure or those with acute symptoms of cardiovascular events were referred immediately to tertiary hospitals as per national protocol and were excluded from the analyses. Treatment decision and patient management followed the Bangladesh national protocol for integrated management of hypertension, diabetes, and cholesterol using a total cardiovascular risk approach (supplementary Figure B).

For peer review only

For peer review only - http://bmjopen.bmj.com/site/about/guidelines.xhtml

Digital platform for care coordination

A digital care coordination platform was developed to address challenges in delivering guideline-based decentralized care in primary healthcare settings. A detailed description of development and deployment of the digital system is available in **supplementary materials**. Briefly, the digital platform was developed in close collaboration with primary health care providers and policymakers through multiple stakeholder meetings and with extensive user-centric research to understand user needs. The system comprised an Android tablet-based application with dashboards to support task shifting/sharing of registering, screening, counselling, and routine patient management. The application allowed community health workers and Community Healthcare Providers to enter patient data, which were accessible to healthcare providers at subdistrict level NCD clinic. With built-in algorithms, the application flagged behavioural risk factors, prompts counselling requirements, highlights elevated levels of blood pressure/glucose, and generated referral recommendations for confirmatory diagnosis and treatment. At the study NCD clinic, a paramedic performed additional examinations and updated patients' data. The digital care coordination platform generated CVD risk scores based on WHO cardiovascular disease risk non-laboratory-based charts (South Asia) and had all pertinent information needed by the medical doctor at the NCD clinic to make treatment decisions and management plans, which were then incorporated into the patient record. Patient dashboards were continuously updated, enabling healthcare providers at community clinics to follow up with routine examination, medication refill, and counselling.

For peer review only - http://bmjopen.bmj.com/site/about/guidelines.xhtml

Data collection and measurements

The present study relied on routinely collected data through the digital care coordination platform. Data were collected during screening and follow-up visits between 1 September 2021 and 28 February 2022. Self-reported sociodemographic characteristics (age, sex, education, religion), health behaviours (tobacco use, physical activity, diet), medical history (diabetes, CVD, chronic kidney disease, respiratory diseases, cancer) were collected by interviewer administered questionnaire installed on tablets. Height was measured with stadiometers (Infant Child Adult ShorrBoard, Weight and Measure LLC), and weight was measured with a digital weighing machine (Tanita UM70). Blood pressure was measured with automatic digital monitor (Omron HEM 7120) following standard operating procedures. Capillary blood was used to measure fasting/random blood glucose with glucometers (Accu-Chek ® Active).

The primary outcome measurements were changes in proportion of people with uncontrolled conditions from baseline to follow-up. Patients were considered to have uncontrolled hypertension if they had SBP≥140 mmHg and DBP≥90 mmHg, and to have uncontrolled diabetes if they had FPG≥7.0 mmol/L or RPG ≥ 11.1mmol/L. Mean changes in blood pressure and blood glucose from baseline were considered as secondary outcomes.

Patient retention was assessed as the incidence of drop out for patients enrolled. Drop-out was defined as no recorded visit for ≥ 2 months. Intervention-related adverse events were documented as safety measurements. This study was approved by the Institutional Review Board of BRAC University (IRB reference number 2020-009-IR). Written informed consent was obtained from all participants.

For peer review only - http://bmjopen.bmj.com/site/about/guidelines.xhtml

Data safety and monitoring

A purpose-built database was developed to facilitate collection, secure storage, and appropriate sharing of study data, including reporting of clinically relevant results to individual participants. The database was commissioned under the supervision of the Chief Investigator and respective site Principal Investigator. Personal and clinical data were separated by pseudonymization to enhance data security. Appropriate authentication and access control mechanisms were implemented. Data were stored securely both locally and in cloud-based infrastructure. All personal identifiers were removed before analysis.

Statistical analysis

Descriptive statistics stratified by patient type (hypertension only, diabetes only, or having both conditions) were reported. Changes in proportion of patient with an uncontrolled condition were analysed with generalized estimating equation (GEE) Poisson regression with robust variance. GEE Poisson is widely used for repeated binary outcomes as it provides consistent estimates of the relative risk and is more stable than the GEE binomial model.[36,37] Change in uncontrolled blood pressure was examined among those with a diagnosis of hypertension (with or without diabetes), while change in uncontrolled blood glucose was evaluated among those with a diagnosis of diabetes, alone or with hypertension. Models were adjusted for age (40-49, 50-59, 60-69, 70-100), sex (male, female), education (no formal education, primary or lower, secondary or higher), religion (Islam, Hindu), BMI category (<18.5, 18.5-22.9, ≥23 kg/m^2), and community clinic location (Khorakhai, Manmathapur, Jahanabad). The

For peer review only - http://bmjopen.bmj.com/site/about/guidelines.xhtml

analyses were repeated for the subset patients who had uncontrolled hypertension or diabetes at enrolment.

As secondary outcomes, changes in SBP and DBP in each of the 5 visits from baseline were assessed with linear mixed effects models among those with a diagnosis of hypertension (with or without diabetes). Changes in mean fasting and random blood glucose were not assessed given the limited number of repeated measurements (patients had either FPG or RPG, not both). We assessed factors associated with loss to follow-up with Poisson models. A logarithm time to drop out was incorporated in incidence ratio estimation. Follow-up time was rounded to roughly one month interval to aid better interpretation. Data management and analyses were conducted using STATA MP v14. Statistical significance was determined by two-sided hypothesis tests ($p<0.05$).

Sensitivity analyses were conducted to assess potential bias introduced by sample attrition with inverse probability weighting. The inverse probability weighting approach weighted the analysis by the inverse of the predicted probability of dropping out from the study. A conditional logit model with the same set of covariates described above was used to predict the probabilities. In addition, we restricted our analyses to patients with at least three follow-up visits to test whether the results were sensitive to better balancing sample composition for timing of enrolment and follow-up.

Patient and public involvement

Before the study, we conducted formative research including a qualitative assessment to explore technology readiness and acceptability of services among the health providers and a quantitative assessment of readiness of health facilities to deliver NCD services. Multiple meetings with primary care physicians, NCD nurses, community

For peer review only - http://bmjopen.bmj.com/site/about/guidelines.xhtml

health workers, and local health authorities were organized to understand the current care pathway and to identify gaps in usual care. Informal discussions with patients regarding their experience with the current model of care were conducted. Patients were not involved in the analysis or interpretation of the data, nor did they contribute to the writing of the manuscript.

Results

Patient characteristics

Of the 2232 people approached, 2208 (98.9%) consented to participate in the study. Among those consented, 2008 people attended the screening of hypertension and diabetes at the community clinics, of whom 829 were found to have possible hypertension and/or diabetes and were referred to the study NCD clinic for further evaluation (Supplementary Figure C). From the 829 referred, 659 attended the study NCD clinic, and amongst them, 624 were confirmed and included in the analytic sample (mean[sd] age, 59.5 [12.0]; 65.1% female, 45.0% had no formal education). There were 404 patients who had hypertension without diabetes, 75 patients who had diabetes without hypertension, 145 patients who had both. At the baseline, of 549 patients with hypertension, 331 (60.3%) had uncontrolled blood pressure, while 163 out of 220 (74.1%) patients with diabetes had uncontrolled glucose levels (**Table 1**).

Changes in blood pressure and blood glucose

Amongst the 549 patients with hypertension, the age- and sex-adjusted proportion of people with uncontrolled blood pressure decreased from 60% (95% CI,

For peer review only

For peer review only - http://bmjopen.bmj.com/site/about/guidelines.xhtml

56%, 64%) at baseline to 26% (95% CI, 19%, 33%) at the third month of follow-up (Figure 2), a 56% (IRR 0.44; 95% CI 0.33, 0.57) reduction in the probability of having uncontrolled blood pressure after adjustment for covariates (Table 2). Among the subset of people with uncontrolled blood pressure at baseline, 65% (IRR 0.35; 95% CI 0.26, 0.47) and a 63% (IRR 0.37; 95% CI 0.18, 0.76) achieved blood pressure controll by the 3rd and 5th visit, respectively.

The mean age- and sex-adjusted SBP at baseline was 145.0 mmHg (95% CI, 143.4, 146.7) among the 549 people with hypertension. Those with uncontrolled BP had higher mean SBP at baseline (158.7 mmHg; 95% CI, 157.0, 160.4) compared with the entire sample of hypertension patients (Supplementary Figure D). A substantial reduction in SBP was observed by the first follow-up visit (-8.0 mmHg; 95% CI, -10.2, -5.9) and the reduction sustained throughout the study period (-8.5 mmHg at 5 months; 95% CI, -16.0, -0.8, Supplementary Table 1). The reduction in SBP was more pronounced among people with uncontrolled hypertension at baseline (first follow-up visit [-15.2 mmHg; 95% CI, -17.8, -12.5]; at the last follow-up visit [-18.3 mmHg; 95% CI, -27.5, -9.1]). Similar patterns of estimates were observed for DBP measures.

We observed a similar pattern of change in the control of blood glucose among the diabetics. At baseline, 74% (95% CI, 68%, 80%) of patient with diabetes had uncontrolled blood glucose. This proportion dropped to 43% (95% CI, 32%, 54%) at the third follow-up visit, and 31% (95% CI, 7.5%, 54.8%) at the fifth follow-up visit. The reduction in the proportion of patients with uncontrolled blood glucose was more pronounced among people with uncontrolled condition at baseline.

For peer review only - http://bmjopen.bmj.com/site/about/guidelines.xhtml

Patient retention, process evaluation, and sensitivity checks

The proportions of patients lost to follow-up immediately after baseline and during the entire study period were 29.1% and 36.2%, respectively (Figure 3). Factors associated with an increased probability of loss to follow-up included having a controlled condition, being a new patient, and only having hypertension at baseline (Supplementary Table 2). Given the nature of continuous enrolment, patients recruited earlier had more follow-up data compared with those entered the study later.

In total, 41% of follow-up visits were carried out at the community clinics. Average daily numbers of patient-visits ranged from 5 – 18 for NCD clinic and 1 – 10 in each community clinic (Supplementary Figure E). Within the 6-month study period, we recorded no deaths, or serious intervention-related adverse events. The results appeared robust in sensitivity analyses incorporating inverse probability weighs for drop-out and excluding respondents with 2 or less follow-up visits (Supplementary Table 3).

Discussion

In this study, we tested a digital technology-supported decentralization of diabetes and hypertension management in rural Bangladesh. The service delivery model with shifting routine hypertension and diabetes services to lowest level primary care facilities and non-physician health workers achieved a high treatment success rate and a relatively high patient retention. The results highlighted the potential of technology supported decentralized care to address critical shortage of health service providers

For peer review only - http://bmjopen.bmj.com/site/about/guidelines.xhtml

and geographical barriers to access, along retention of patients with hypertension and diabetes.

Primary care delivery of hypertension and diabetes services in Bangladesh is hampered by a critical shortage of trained health workers and poor geographical accessibility. In Bangladesh, only about one in three patients with hypertension or diabetes are currently on treatment.[28,29] The government NCD Corners at subdistrict level are remote and poorly accessible to a large body of population living with NCDs.[13] As a result, the majority of people with hypertension and diabetes are dependent on the largely unregulated private sector, and frequently received services from untrained or unqualified care providers.[38]

Our preliminary results showed that with the support of a digital care coordination platform and proper training, non-physician health workers based in the community or community clinics can successfully deliver screening for hypertension and diabetes, routine follow-up, counselling, drug refill, and other aspects of NCD management. This approach of healthcare delivery has the potential to substantially improve access to healthcare and reduce the burden on the physician-managed NCD clinics. Overall, the proportion of patient follow-up visits carried out at community clinics was 41%. Routine patient follow-up at community location could be further enhanced by introducing telemedicine and dose titration by non-physician health workers. Through technology-supported decentralization and the involvement of the large number of peripheral primary care facilities, hypertension and diabetes services could be rapidly expanded in Bangladesh and similar global settings.

For peer review only - http://bmjopen.bmj.com/site/about/guidelines.xhtml

In addition to the lack of human resources, inconsistent adherence to treatment protocol among health care providers, poor patient management, and irregular follow-up are important obstacles for effective hypertension and diabetes management.[13,39] As a result of these barriers, compounded by low levels of awareness and screening, the proportion of all patients with controlled condition is merely 7% for diabetes and 12% for hypertension in Bangladesh.[29,30] A variety of digital tools have been examined for hypertension and diabetes care quality improvement in South Asian settings with mixed findings.[23,24,40,41] Digital tools can be invaluable in strengthening health systems for NCD care delivery, however, they need to be an integral part of an multifaceted intervention, with thoughtful consideration of care pathways and user interfaces, to exert effects within specific health system contexts.[6] Following international standards of interoperability throughout the designing phrase, the digital platform resources developed in the present study are open-source and can be readily utilized by the global health community.

Treatment adherence and retention are major challenges in NCD management, particularly in LMICs. [42-44] In a recent study of task shifting to non-physician clinicians for integrated management of hypertension and diabetes in rural Cameron, Labhardt and colleagues reported a retention rate of 18.1% among treated patient at one year. Another community health worker-led, technology-enabled private sector intervention for diabetes and hypertension management among urban poor in India observed a 44.0% retention rate at 6 months.[44] The observed retention rates of 63.8% by the end of the present study appeared high. While the reasons were formally tested, possible

For peer review only - http://bmjopen.bmj.com/site/about/guidelines.xhtml

explanations include the ease of obtaining medication refill, alleviated travel-related burden, and more counselling time with non-physician health workers.

As a proof-of-concept study, the project comprised an uncontrolled and unblinded evaluation of a decentralized service model, supported by a digital care coordination platform. Nevertheless, the observed rates of 61% for blood pressure control and 69% for blood glucose control at the end of the study compared favourably with existing implementation studies and clinical trials of multicomponent health system interventions in developing countries.[45-48] For example, a recent cluster-randomized controlled trail in Bangladesh, India, and Sri Lanka, a multifaceted intervention involving trained community health workers for home-based blood pressure monitoring and counselling achieved blood pressure control in 53.2% of the participants in the intervention group and 43.7% of the control group.[46] The findings will inform the design of our planned further studies to test the feasibility and utility of the interventions within government primary care system, as well as to evaluate the effectiveness of the digital technology supported decentralized care in Bangladesh.

Conclusion

In the proof-of-concept study, a digital technology supported decentralized care model achieved high levels of blood pressure and blood glucose control among patients with hypertension and diabetes in Bangladesh. Our results suggest that with proper training, supportive supervision, and access to digital technology, non-physician health workers at peripheral primary facilities can deliver health screening, counselling, routine drug refill, and other aspects of patient management for people with hypertension and

For peer review only - http://bmjopen.bmj.com/site/about/guidelines.xhtml

diabetes. The present study highlighted the potential to involve peripheral primary care facilities and community health workers to rapidly expand much needed hypertension and diabetes services in Bangladesh, and similar global settings. Further investigations are needed to evaluate the effectiveness of decentralized hypertension and diabetes care.

For peer review only

References

1 Sun, H. *et al.* IDF Diabetes Atlas: Global, regional and country-level diabetes prevalence estimates for 2021 and projections for 2045. *Diabetes research and clinical practice* **183**, 109119 (2022).

2 Roth, G. A. *et al.* Global Burden of Cardiovascular Diseases and Risk Factors, 1990–2019: Update From the GBD 2019 Study. *Journal of the American College of Cardiology* **76**, 2982-3021 (2020). https://doi.org:https://doi.org/10.1016/j.jacc.2020.11.010

3 Prabhakaran, D. *et al.* Cardiovascular, respiratory, and related disorders: key messages from Disease Control Priorities. *The Lancet* **391**, 1224-1236 (2018).

4 Zhou, B. *et al.* Worldwide trends in hypertension prevalence and progress in treatment and control from 1990 to 2019: a pooled analysis of 1201 population-representative studies with 104 million participants. *The Lancet* **398**, 957-980 (2021).

5 Organization, W. H. Global action plan for the prevention and control of NCDs 2013–2020. 2013. *Geneva: WHO* (2019).

6 Manne-Goehler, J. *et al.* Health system performance for people with diabetes in 28 low-and middle-income countries: a cross-sectional study of nationally representative surveys. *PLoS medicine* **16**, e1002751 (2019).

7 Geldsetzer, P. *et al.* The state of hypertension care in 44 low-income and middle-income countries: a cross-sectional study of nationally representative individual-level data from 1· 1 million adults. *The Lancet* **394**, 652-662 (2019).

8 Kabir, A., Karim, M. N., Islam, R. M., Romero, L. & Billah, B. Health system readiness for non-communicable diseases at the primary care level: a systematic review. *BMJ open* **12**, e060387 (2022).

9 Frenk, J. *et al.* Health professionals for a new century: transforming education to strengthen health systems in an interdependent world. *The lancet* **376**, 1923-1958 (2010).

10 Chowdhury, H. A. *et al.* Assessing service availability and readiness of healthcare facilities to manage diabetes mellitus in Bangladesh: Findings from a nationwide survey. *PloS one* **17**, e0263259 (2022).

11 Islam, K., Huque, R., Saif-Ur-Rahman, K., ANM, E. K. & AHM, E. H. Implementation status of non-communicable disease control program at primary health care level in Bangladesh: Findings from a qualitative research. *Public Health in Practice*, 100271 (2022).

12 Organization, W. H. Declaration of alma-ata. (World Health Organization. Regional Office for Europe, 1978).

13 Rawal, L. B. *et al.* Non-communicable disease (NCD) corners in public sector health facilities in Bangladesh: a qualitative study assessing challenges and opportunities for improving NCD services at the primary healthcare level. *BMJ Open* **9**, e029562 (2019). https://doi.org:10.1136/bmjopen-2019-029562

14 Rawal, L., Jubayer, S., Choudhury, S. R., Islam, S. M. S. & Abdullah, A. S. Community health workers for non-communicable diseases prevention and control in Bangladesh: a qualitative study. *Global Health Research and Policy* **6**, 1 (2021). https://doi.org:10.1186/s41256-020-00182-z

For peer review only - http://bmjopen.bmj.com/site/about/guidelines.xhtml

15 Devi, R., Kanitkar, K., Narendhar, R., Sehmi, K. & Subramaniam, K. A Narrative Review of the Patient Journey Through the Lens of Non-communicable Diseases in Low- and Middle-Income Countries. *Advances in Therapy* **37**, 4808-4830 (2020). https://doi.org:10.1007/s12325-020-01519-3

16 Jindal, D. *et al.* Improving care for hypertension and diabetes in india by addition of clinical decision support system and task shifting in the national NCD program: I-TREC model of care. *BMC Health Services Research* **22**, 1-12 (2022).

17 Brennan, A. T. *et al.* Outcomes of stable HIV-positive patients down-referred from a doctor-managed antiretroviral therapy clinic to a nurse-managed primary health clinic for monitoring and treatment. *Aids* **25**, 2027-2036 (2011).

18 Sanne, I. *et al.* Nurse versus doctor management of HIV-infected patients receiving antiretroviral therapy (CIPRA-SA): a randomised non-inferiority trial. *The Lancet* **376**, 33-40 (2010).

19 Lehmann, U., Van Damme, W., Barten, F. & Sanders, D. Task shifting: the answer to the human resources crisis in Africa? *Human resources for health* **7**, 1-4 (2009).

20 Kruk, M. E., Nigenda, G. & Knaul, F. M. Redesigning primary care to tackle the global epidemic of noncommunicable disease. *American journal of public health* **105**, 431-437 (2015).

21 Kredo, T., Ford, N., Adeniyi, F. B. & Garner, P. Decentralising HIV treatment in lower‐and middle‐income countries. *Cochrane database of systematic reviews* (2013).

22 Labrique, A. B. *et al.* Best practices in scaling digital health in low and middle income countries. *Globalization and health* **14**, 1-8 (2018).

23 Anchala, R. *et al.* Evaluation of effectiveness and cost‐effectiveness of a clinical decision support system in managing hypertension in resource constrained primary health care settings: results from a cluster randomized trial. *Journal of the American Heart Association* **4**, e001213 (2015).

24 Prabhakaran, D. *et al.* Effectiveness of an mHealth-Based Electronic Decision Support System for Integrated Management of Chronic Conditions in Primary Care. *Circulation* **139**, 380-391 (2019). https://doi.org:doi:10.1161/CIRCULATIONAHA.118.038192

25 Kumar, A. *et al.* Designing and implementing an integrated non-communicable disease primary care intervention in rural Nepal. *BMJ global health* **4**, e001343 (2019).

26 Vos, T. *et al.* Global burden of 369 diseases and injuries in 204 countries and territories, 1990–2019: a systematic analysis for the Global Burden of Disease Study 2019. *The Lancet* **396**, 1204-1222 (2020).

27 Sathi, N. J., Islam, M. A., Ahmed, M. S. & Islam, S. M. S. Prevalence, trends and associated factors of hypertension and diabetes mellitus in Bangladesh: Evidence from BHDS 2011 and 2017–18. *PloS one* **17**, e0267243 (2022).

28 Khan, N., Oldroyd, J. C., Hossain, M. B. & Islam, R. M. Awareness, treatment, and control of diabetes in Bangladesh: evidence from the Bangladesh Demographic and Health Survey 2017/18. *International Journal of Clinical Practice* **2022** (2021).

29 Khan, M. N. *et al.* Prevalence, awareness, treatment, and control of hypertension in Bangladesh: Findings from National Demographic and Health Survey, 2017–2018. *The Journal of Clinical Hypertension* **23**, 1830-1842 (2021).

For peer review only - http://bmjopen.bmj.com/site/about/guidelines.xhtml

30 Khan, M. N., Oldroyd, J. C., Hossain, M. B. & Islam, R. M. Awareness, treatment, and control of diabetes in Bangladesh: evidence from the Bangladesh Demographic and Health Survey 2017/18. *medRxiv* (2021).
31 Riaz, B. K. *et al.* Risk factors for non-communicable diseases in Bangladesh: findings of the population-based cross-sectional national survey 2018. *BMJ open* **10**, e041334 (2020).
32 Non-communicable Disease Control Programme, D. G. o. H. S. (2018).
33 Bank, W. *Hypertension and Type-2 Diabetes in Bangladesh: Continuum of Care Assessment and Opportunities for Action*. (World Bank, 2018).
34 Government of People's Republic of Bangladesh, M. o. H. a. F. W. (2022).
35 Organization, W. H. WHO package of essential noncommunicable (PEN) disease interventions for primary health care. (2020).
36 Zou, G. & Donner, A. Extension of the modified Poisson regression model to prospective studies with correlated binary data. *Statistical methods in medical research* **22**, 661-670 (2013).
37 Yelland, L. N., Salter, A. B. & Ryan, P. Performance of the modified Poisson regression approach for estimating relative risks from clustered prospective data. *American journal of epidemiology* **174**, 984-992 (2011).
38 BRAC. Accessible and affordable primary health care for all: annual report progress of social enterprises April 2018 - April 2019. (BRAC Health Nutrition and Population Program, 2020).
39 Alam, B. B. *et al.* Hypertension and Type-2 diabetes in Bangladesh: continuum of care assessment and opportunities for action. (2020).
40 Venkateshmurthy, N. S. *et al.* m-Power Heart Project-a nurse care coordinator led, mHealth enabled intervention to improve the management of hypertension in India: study protocol for a cluster randomized trial. *Trials* **19**, 1-9 (2018).
41 Peiris, D. *et al.* SMARThealth India: a stepped-wedge, cluster randomised controlled trial of a community health worker managed mobile health intervention for people assessed at high cardiovascular disease risk in rural India. *PLoS One* **14**, e0213708 (2019).
42 Labhardt, N. D., Balo, J.-R., Ndam, M., Grimm, J.-J. & Manga, E. Task shifting to non-physician clinicians for integrated management of hypertension and diabetes in rural Cameroon: a programme assessment at two years. *BMC health services research* **10**, 1-10 (2010).
43 Kachimanga, C. *et al.* Implementation of a non-communicable disease clinic in rural Sierra Leone: early experiences and lessons learned. *Journal of public health policy* **42**, 422-438 (2021).
44 Deo, S. & Singh, P. Community health worker-led, technology-enabled private sector intervention for diabetes and hypertension management among urban poor: a retrospective cohort study from large Indian metropolitan city. *BMJ Open* **11**, e045246 (2021). https://doi.org:10.1136/bmjopen-2020-045246
45 He, J. *et al.* Effect of a Community Health Worker–Led Multicomponent Intervention on Blood Pressure Control in Low-Income Patients in Argentina: A Randomized Clinical Trial. *JAMA* **318**, 1016-1025 (2017). https://doi.org:10.1001/jama.2017.11358

For peer review only - http://bmjopen.bmj.com/site/about/guidelines.xhtml

46 Jafar, T. H. *et al.* A community-based intervention for managing hypertension in rural South Asia. *New England Journal of Medicine* **382**, 717-726 (2020).

47 Schwalm, J.-D. *et al.* A community-based comprehensive intervention to reduce cardiovascular risk in hypertension (HOPE 4): a cluster-randomised controlled trial. *The Lancet* **394**, 1231-1242 (2019). https://doi.org:https://doi.org/10.1016/S0140-6736(19)31949-X

48 Chow, C. K. *et al.* Prevalence, awareness, treatment, and control of hypertension in rural and urban communities in high-, middle-, and low-income countries. *Jama* **310**, 959-968 (2013).

For peer review only

For peer review only - http://bmjopen.bmj.com/site/about/guidelines.xhtml

Table 1. Patient characteristics at enrolment

Characteristics *	Entire Sample (n=624)	Hypertension only (n=404)	Diabetes only (n=75)	Hypertension & Diabetes (n=145)
Age in years, mean (sd)	59.5 (12.0)	61.5 (12.3)	55.1 (10.7)	56.4 (10.6)
Age				
40-49	136 (21.8)	68 (16.8)	28 (37.3)	40 (27.6)
50-59	179 (28.7)	110 (27.2)	20 (26.7)	49 (33.8)
60-69	169 (27.1)	116 (28.7)	18 (24.0)	35 (24.1)
70-100	140 (22.4)	110 (27.2)	9 (12.0)	21 (14.5)
Sex				
Female	406 (65.1)	274 (67.8)	41 (54.7)	91 (62.4)
Male	218 (34.9)	130 (32.2)	34 (45.3)	54 (37.6)
Religion				
Hindu	78 (12.5)	57 (14.1)	4 (5.3)	17 (11.7)
Islam	546 (87.5)	347 (85.9)	71 (94.7)	128 (88.3)
Education				
No formal school	281 (45.0)	199 (49.3)	24 (32.0)	58 (40.0)
Primary or lower	172 (27.6)	118 (29.2)	22 (29.3)	32 (22.1)
Secondary or higher	171 (27.4)	87 (21.5)	29 (38.7)	55 (37.9)
Community clinic				
Khorakhai	204 (32.7)	120 (29.7)	33 (44.0)	51 (35.2)
Jahanabad	202 (32.4)	132 (32.7)	23 (30.7)	47 (32.4)
Manmathapur	218 (34.9)	152 (37.6)	19 (25.3)	47 (32.4)
Tabaco use				
No	322 (51.6)	203 (50.3)	38 (50.7)	81 (55.9)
Yes	302 (48.4)	201 (49.7)	37 (49.3)	64 (44.1)
BMI categories				
<18.5 kg/m^2	68 (10.9)	55 (13.6)	6 (8.0)	7 (4.8)
18.5-22.9 kg/m^2	243 (38.9)	159 (39.4)	26 (34.7)	58 (40.0)
≥23.0 kg/m^2	313 (50.2)	190 (47.0)	43 (57.3)	80 (55.2)
Hypertension				
Baseline BP controlled	281 (39.7)	145 (35.9)	n/a	73 (50.3)
Baseline BP uncontrolled †	331 (60.3)	259 (64.1)	n/a	72 (49.7)
Diabetes				
Baseline glucose controlled	57 (25.9)	n/a	15 (20.0)	42 (29.0)
Baseline glucose uncontrolled †	163 (74.1)	n/a	60 (80.0)	103 (71.0)

BP, blood pressure; n/a, not applicable
* n (%) are presented, unless indicated otherwise.
† Uncontrolled blood pressure is defined as systolic blood pressure ≥140 mmHg or diastolic blood pressure ≥90 mmHg, while uncontrolled blood glucose is defined as fasting blood glucose ≥7 mmol/L or random blood glucose ≥11.1 mmol/L.

For peer review only - http://bmjopen.bmj.com/site/about/guidelines.xhtml

Table 2. Change in proportion of uncontrolled conditions over follow-up visits

Visit	Blood pressure		Blood glucose	
	Entire sample	Baseline uncontrolled cases †	Entire sample	Baseline uncontrolled cases ‡
1 month	0.63 (0.55, 0.72)	0.53 (0.47, 0.61)	0.69 (0.58, 0.82)	0.57 (0.48, 0.67)
2 months	0.48 (0.39, 0.60)	0.38 (0.30, 0.47)	0.66 (0.54, 0.81)	0.51 (0.41, 0.63)
3 months	0.44 (0.33, 0.57)	0.35 (0.26, 0.47)	0.60 (0.45, 0.78)	0.49 (0.38, 0.64)
4 months	0.49 (0.35, 0.69)	0.38 (0.27, 0.53)	0.54 (0.36, 0.80)	0.41 (0.26, 0.62)
5 months	0.64 (0.32, 1.25)	0.37 (0.18, 0.76)	0.44 (0.20, 0.96)	0.16 (0.05, 0.54)
Constant	0.44 (0.30, 0.64)	0.99 (0.93, 1.05)	1.04 (0.67, 1.60)	1.33 (0.98, 1.78)
N	549	331	220	163

* Models adjusted for age (40-49, 50-59, 60-69, 70-100), sex (male, female), education (no formal education, primary or lower, secondary or higher), religion (Islam, Hindu), BMI category (under, normal, overweight or obese), community clinics (Khorakhai, Manmathapur, Jahanabad).
† Baseline uncontrolled cases were defined patients with systolic blood pressure ≥ 140 mmHg or diastolic blood pressure ≥ 90 mmHg
‡ Baseline uncontrolled cases were defined patients with fasting plasma glucose ≥ 7.0 mmol/L or random plasma glucose ≥ 11.1 mmol/L

For peer review only

For peer review only - http://bmjopen.bmj.com/site/about/guidelines.xhtml

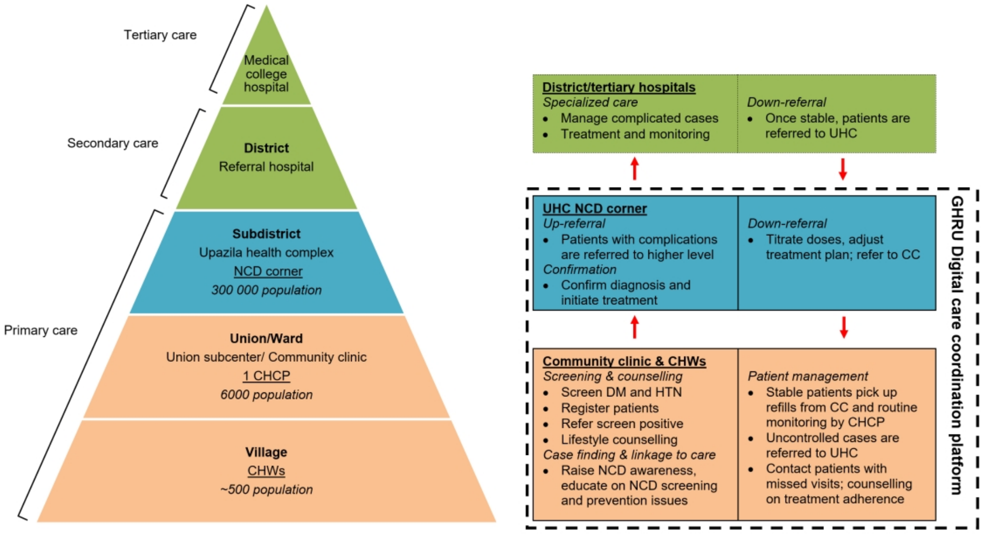

Figure 1. NCD service delivery hierarchy and coordinated NCD care

GHRU global health research unit; CHCP community health care provider; CHW community health worker; CC community clinic; UHC Upazila Health Complex; NCD non-communicable disease* Government community health workers include family welfare assistants, health assistants, and multipurpose health volunteers; however, a designated community health worker was recruited and trained for NCD related tasks in the present study. Union subcentres were not involved in this present study. Union subcentres are at higher level of the hierarchy than community clinics.

487x297mm (144 x 144 DPI)

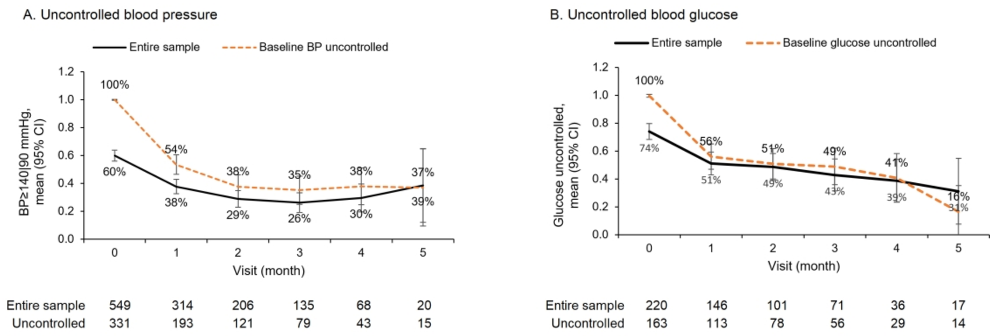

Figure 2. Change in proportion of uncontrolled conditions over follow-up visits

BP blood pressure Results adjusted age and sex Uncontrolled blood pressure is defined as systolic blood pressure ≥140 mmHg or diastolic blood pressure ≥90 mmHg, while uncontrolled blood glucose is defined as fasting blood glucose ≥7 mmol/L or random blood glucose ≥11.1 mmol/L.

563x190mm (144 x 144 DPI)

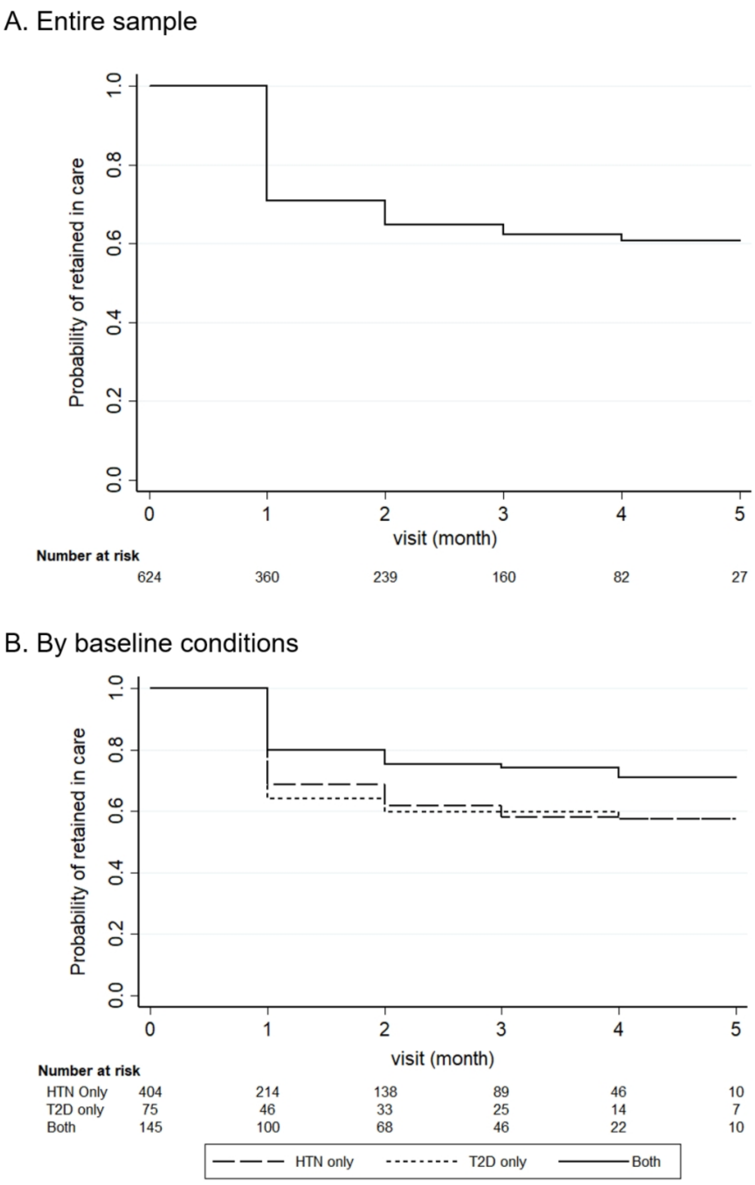

Figure 3. Patient loss to follow-up by baseline diagnosis

HTN hypertension; T2D type 2 diabetes * Loss to follow-up is defined as no consultation recorded for two months.Survival function estimated by the Kaplan-Meier method.Time interval between two adjacent visits is approximately one month.

203x288mm (144 x 144 DPI)

Supplementary Materials

TITLE: Enhancing Care Quality and Accessibility through Digital Technology-Supported Decentralization of Hypertension and Diabetes Management: A Proof-of-Concept Study in Rural Bangladesh

For peer review only

For peer review only - http://bmjopen.bmj.com/site/about/guidelines.xhtml

The development and deployment of a digital platform for primary care hypertension and diabetes care coordination in Bangladesh

Background

The long-term nature of hypertension and diabetes (and other noncommunicable diseases), which require regular engagement and health monitoring for improved patient outcomes, make it critical for healthcare personnel in primary health care (PHC) teams to be collaborative and coordinated to ensure efficient and effective care delivery.[1,2] Digital platforms have the potential to enable greater collaboration and coordination that will be helpful in addressing many of the barriers to effective NCD management within the PHC system.[3] Digital platforms can help the health care system to document the risks and health states of the population, and to prioritize delivery of care to those at greatest need. Through more efficient data capture and sharing, guideline-based decision support, generation of individualized care plans and management of care delivery actions, the implementation of digital platforms support the move towards coordinated, evidence-based and data-driven NCD management for populations served by the PHC team.[4]

Identification of gaps in usual care

The first step in identifying the components of the intervention was to assess the gaps in usual care through systematic reviews of literature on epidemiological studies, NCD service delivery in LMIC and in Bangladesh, and through discussion with experts and stake holders. Multiple meetings with primary care physicians, NCD nurses, community health workers, and local health authorities were organized to understand the current care pathway, and to identify gaps in usual care. Consultations with domain experts, including cardiologists, nutritionists, epidemiologists, statisticians, data scientists, pharmacists, were conducted. The team mapped out the journey of patients under current model of government service delivery and a model of decentralized care with the involvement of the most peripheral primary care facilities and community-based interventions by community health workers (CHWs).

Through these processes, a few key gaps were identified include 1) using of paper-based health records limits exchange of critical information, 2) adherence to national guidelines and treatment protocols is inconsistent among health care providers, 3) patient management of medication adherence and follow-up are irregular, 4) lack of coordination in service delivery which involves a wide range of settings and personnel, which included subdistrict level primary care facilities, community clinics - the most peripheral primary health care facilities, and doctors, nurses, community healthcare providers (CHCPs), and CHWs.

For peer review only - http://bmjopen.bmj.com/site/about/guidelines.xhtml

Overview of the development processes

The digital platform was developed using the user-centred design (UCD), an iterative software development methodology in which the development team focuses on the users and their needs in each phase of the development process.[5] A multi-disciplinary technology development team consisting of domain experts, software engineers and user design researchers utilized UCD in the development of the Coordinate platform that with each iteration, produced a usable version of the software that was increasingly aligned with the needs of its users. The process was completed when a sufficiently developed version of the software was produced that could be deployed for use in the study.

One of the core value propositions of Coordinate platform is to use it in the community. In rural areas of Bangladesh, internet connectivity is a major problem, and many online platforms need to adopt offline functionality to operate in rural areas. Hence, offline functionality of the Coordinate platform was designed with different use cases and functionalities.

The Android OS was chosen as the operating system platform due to the greater flexibility it offers in app development, testing and deployment, along with users' familiarity due to widespread usage in South Asia and the availability of low-cost tablets. The platform is underpinned by an administrator web-dashboard application built using Laravel, an open-source PHP framework and connected to a database built using PostgresSQL, open-source relational database management system. The platform was development through four iterative loops from June 2020 to December 2021. The platform was designed following international standards of interoperability, with clearly documented application programming interfaces. The digital platform resources are open-source and can be readily utilized by the global health community.

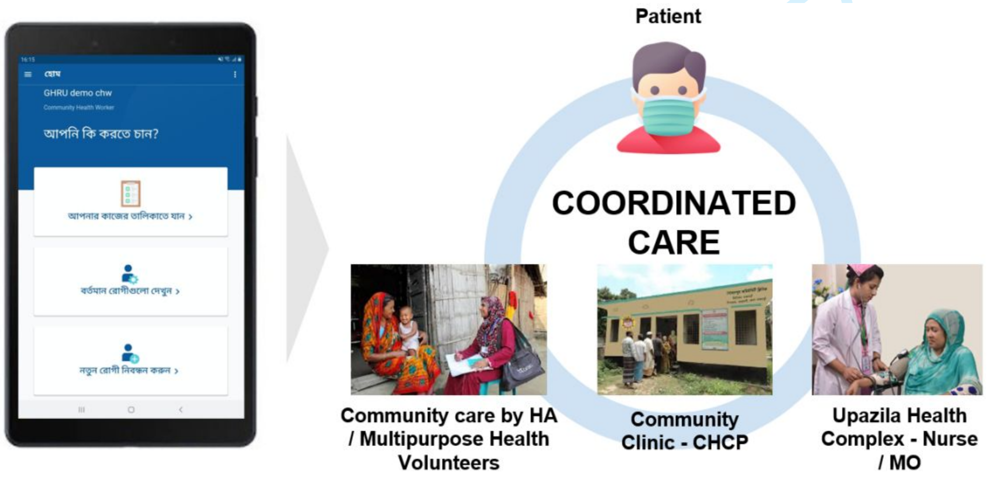

Figure 1. digital care coordination platform encompassing all primary care providers and community health workers

For peer review only - http://bmjopen.bmj.com/site/about/guidelines.xhtml

Content and functionalities

Based on the results from the need assessment, the digital platform was built to support the needs of various health service providers, including new users (CHCPs, CHWs) who would be trained to provided care in a decentralized care model (Figure 1). Accordingly, a core set of functionalities was developed to support the coordination of patient care through enhanced data capture and sharing, guideline-based decision support, generation of individualized care plans and management of care delivery actions (Figure 2).

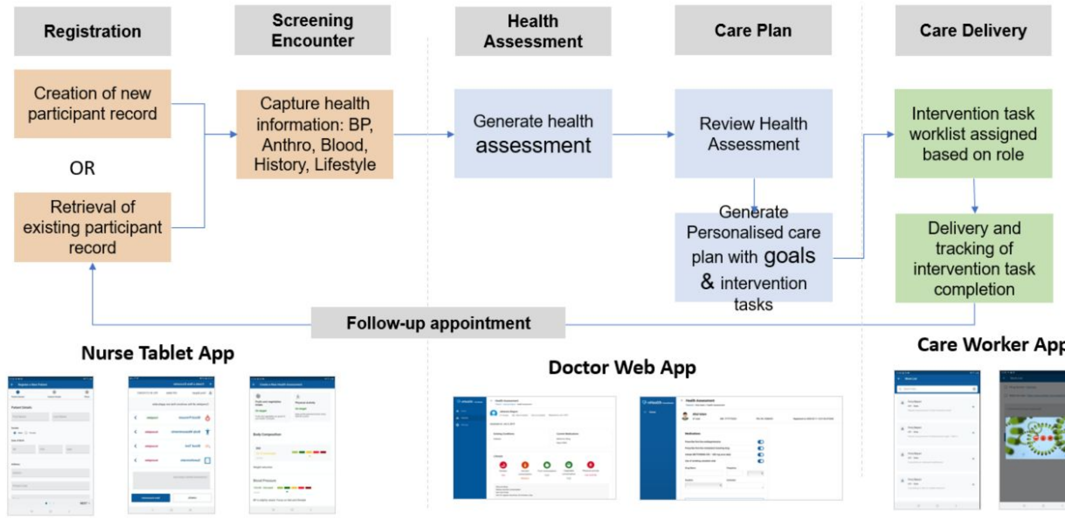

Figure 2. digital platform designed to support multiple healthcare personnel roles along the care pathway

The system consists of a tablet-based application for community health workers to register participants at risk, administer in-field questionnaire, deliver care plan, and refer. CHCPs at community clinics have access to information entered by CHWs. CHCPs will then perform a simplified structured clinical evaluation, including medical history, anthropometrics, blood pressure, and blood glucose. Based on the information obtained, the CHCPs conduct lifestyle education for high-risk behaviours with assistance from built-in point-of-care support and referred the participant with elevated risks for further confirmation at subdistrict NCD clinic. The participant's electronic health record will be updated to reflect the additional information obtained by CHCPs and be available to nurses and doctors at NCD clinic.

Once a participant approaches NCD clinic, nurses perform additional examinations and update patient data. The platform generates CVD risk scores based on WHO cardiovascular disease risk non-laboratory-based charts (South Asia) and have all pertinent data for physicians to make treatment decision and management plan, which are then incorporated into patient profile. The patient list is then generated and

For peer review only - http://bmjopen.bmj.com/site/about/guidelines.xhtml

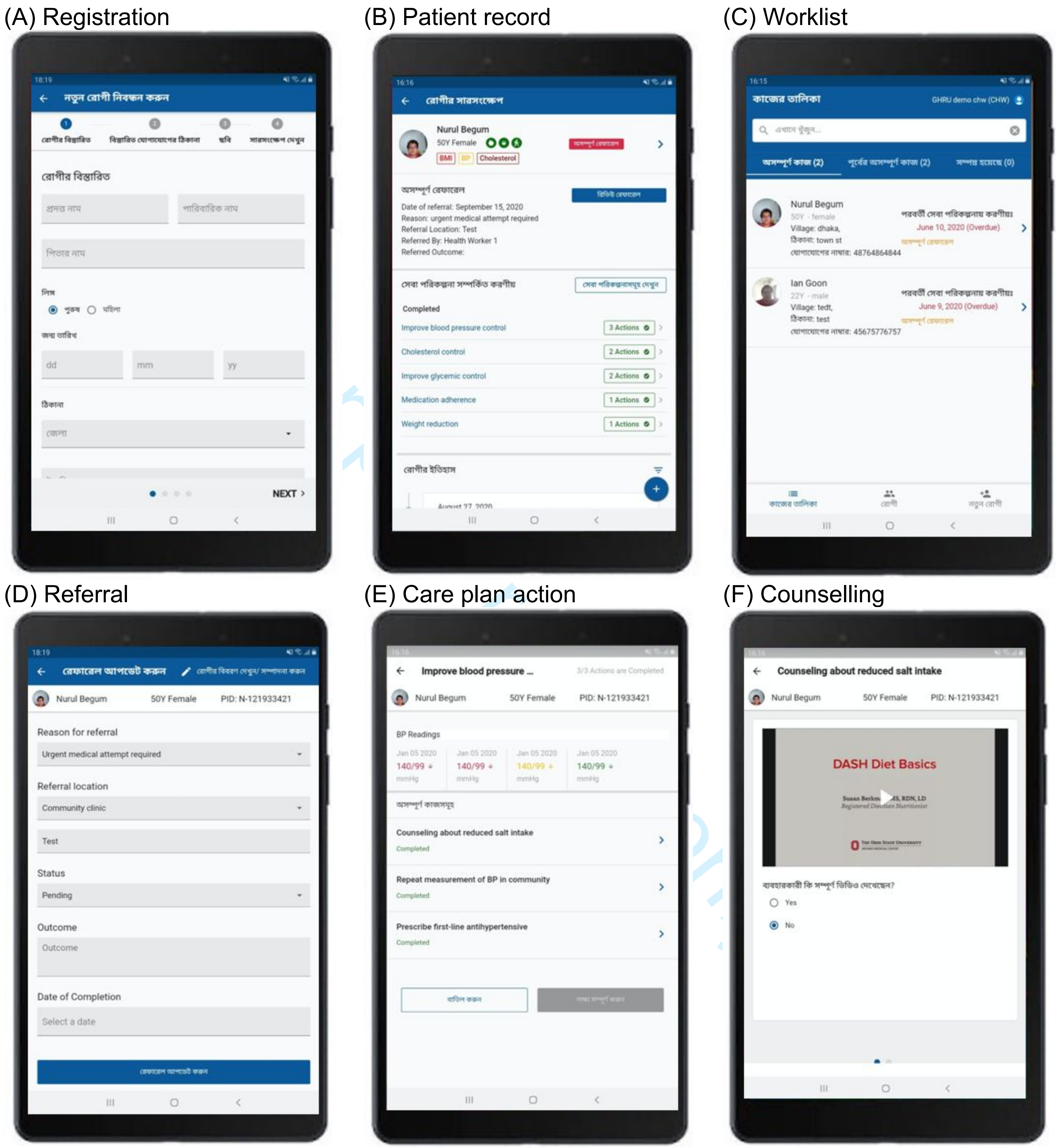

Figure 3. User interfaces for (A) registration, (B) patient record, (C) worklist, (D) referral, (E) care plan action, (F) counselling support

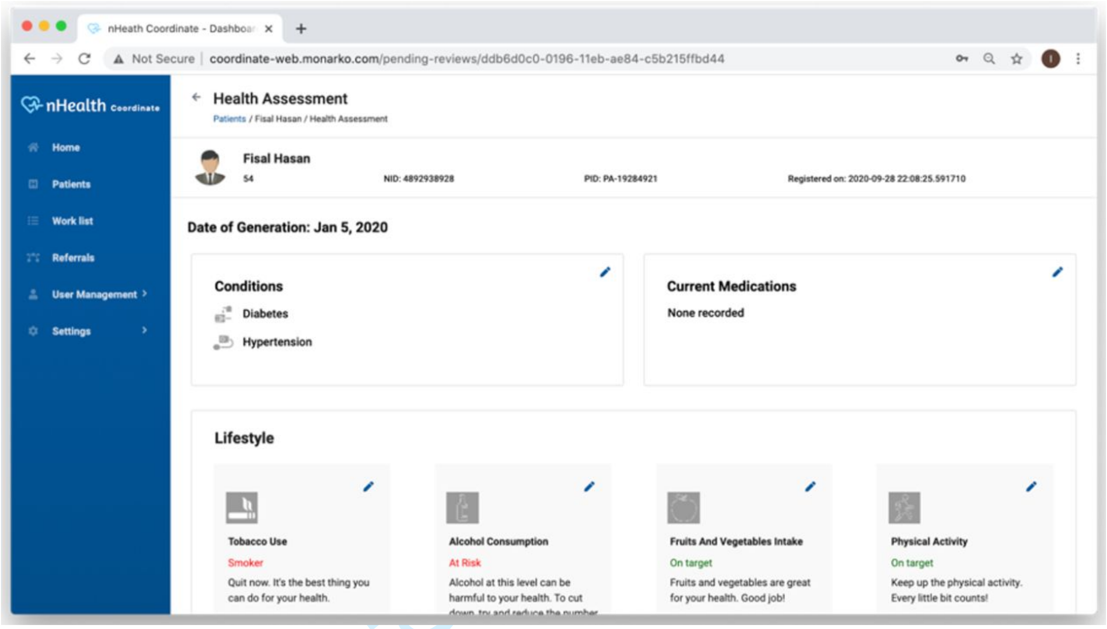

Figure 4. Central administration dashboard

continuously updated for CHWs in the community and CHCPs at community clinics to follow up with routine examination, medication refill, and counselling. Some of the user interfaces were illustrated in Figure 3.

The doctors and nurses have access to a web-based central administration dashboard which allow them to track patient treatment progress and referrals, and to add/remove users (Figure 4). A demo video overview of the platform is available at https://youtu.be/nXmlX0_RZVY.

Conclusions

The digital platform is among the first to support a coordinated primary care delivery for hypertension and diabetes management across all levels of primary care providers and community health workers. Digital tools can be invaluable in strengthening health systems, although they need to be an integral part of an intervention package to exert effects within specific health system contexts.[6] Multifaceted intervention is often needed to maximize the impact of a digital tool.[2] Following international standards of interoperability throughout the designing phrase, the digital platform resources are open-source and can be readily utilized by the global health community.

In the next steps, we are planning to add training and education of primary care providers and patient self-management components to the platform. Specifically, we planned to develop online training program in collaboration with domain experts and key stakeholders. Digital training modules will be developed for use by PHC teams, tailored

For peer review only - http://bmjopen.bmj.com/site/about/guidelines.xhtml

to user role and setting. These highly scalable modules for development of NCD care skills may reduce the need for in-person sessions. In addition, to empower community users, we planned to develop a user-facing module that gives participants access to their health information, personalised care plans, and evidence-based self-management content. Finally, we will refine and extend on the open-source decision-support algorithms in our platform to simplify the process of tailoring to local context, and to enhance formulation of adaptive longitudinal care plans. This includes taking into account additional parameters in decision-making to strengthen care plan recommendations and drive effective disease management.

References

1. Kabir, A., Karim, M. N., Islam, R. M., Romero, L. & Billah, B. Health system readiness for non-communicable diseases at the primary care level: a systematic review. BMJ open 12, e060387 (2022).
2. Kruk, M. E., Nigenda, G. & Knaul, F. M. Redesigning primary care to tackle the global epidemic of noncommunicable disease. American journal of public health 105, 431-437 (2015).
3. Labrique, A. B. et al. Best practices in scaling digital health in low and middle income countries. Globalization and health 14, 1-8 (2018).
4. Mechael, P. et al. Barriers and gaps affecting mHealth in low and middle income countries: Policy white paper. (2010).
5. Bazzano AN, Martin J, Hicks E, Faughnan M, Murphy L. Human-centred design in global health: A scoping review of applications and contexts. Virgili G, editor. PLOS ONE 2017 Nov 1;12(11):e0186744.
6. Praveen, D., et al. (2014). "SMARTHealth India: development and field evaluation of a mobile clinical decision support system for cardiovascular diseases in rural India." JMIR mHealth and uHealth **2**(4): e3568.

For peer review only

For peer review only - http://bmjopen.bmj.com/site/about/guidelines.xhtml

Supplementary Figure A. Study location (Parbatipur subdistrict, Dinajpur district)

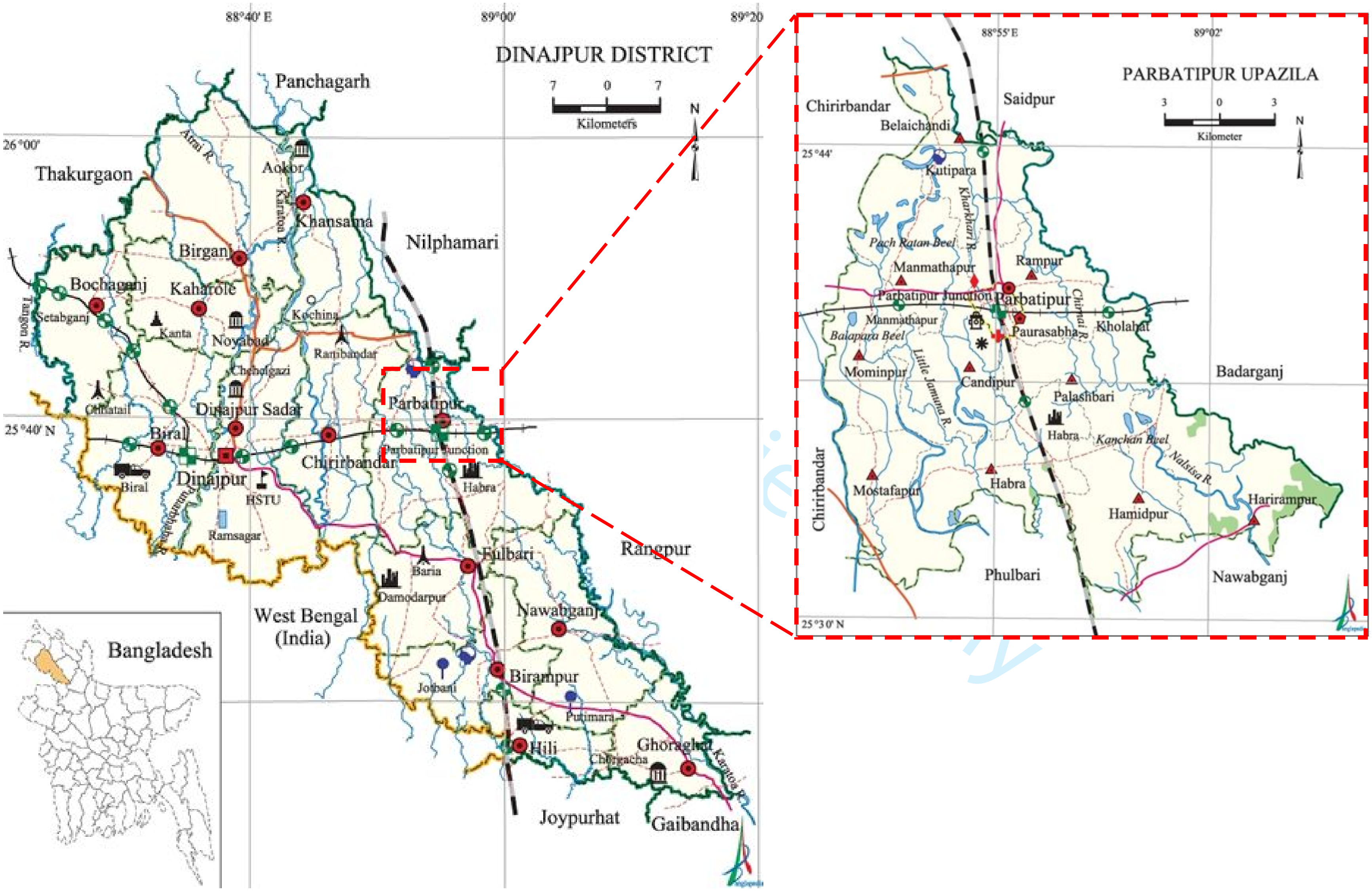

Supplementary Figure B. Bangladesh national protocol for integrated management of hypertension, diabetes and cholesterol using a total cardiovascular risk approach

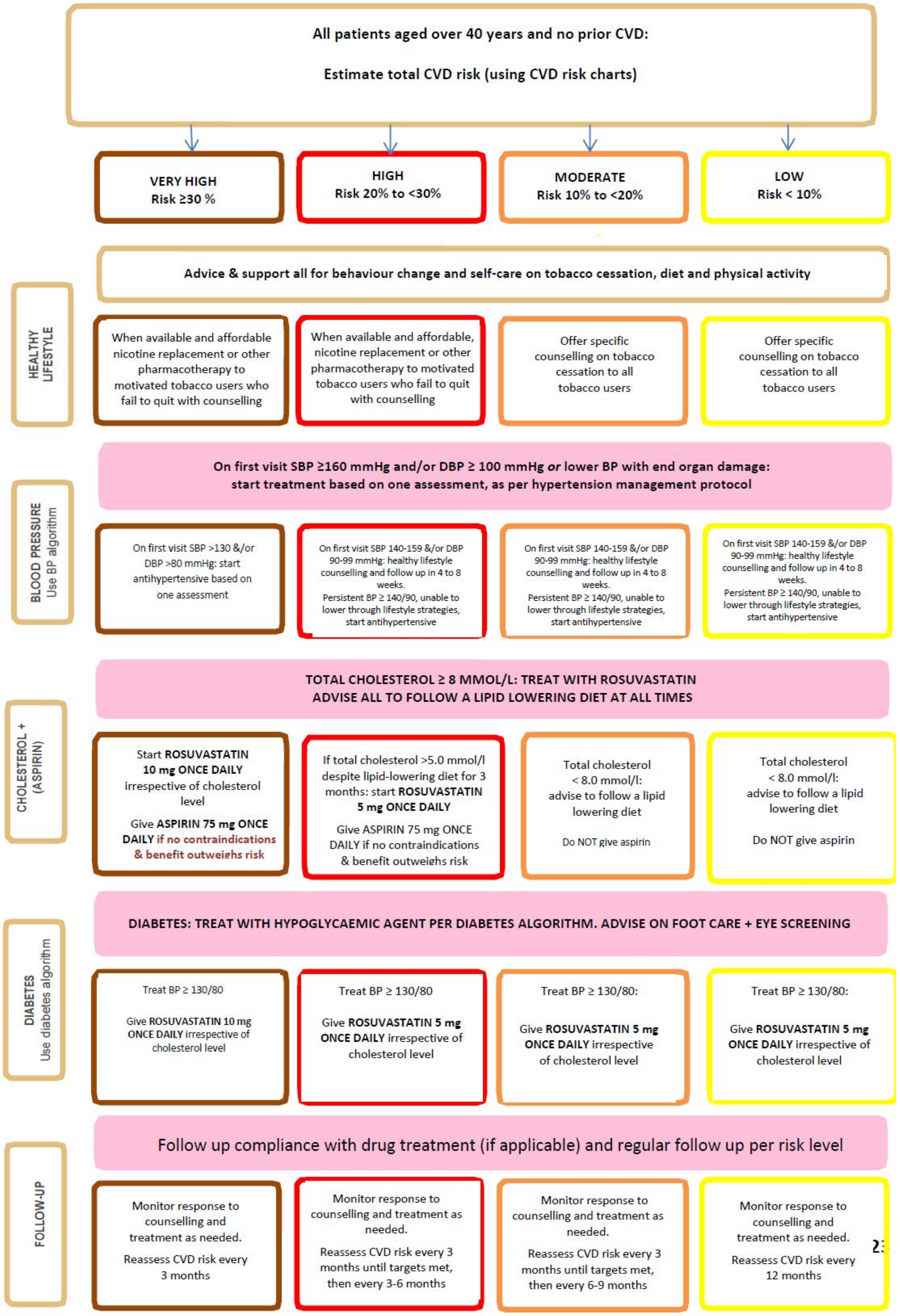

For peer review only - http://bmjopen.bmj.com/site/about/guidelines.xhtml

Bangladesh national protocol for management of hypertension at primary health care settings

Decision to start antihypertensive medication taken based on estimation of total cardiovascular disease (CVD) risk of patients aged ≥ 40 years [a, b]

Step 1: **START AMLODIPINE 5 mg ONCE DAILY** [c]

Step 2: Review after 1 month, if treatment target not met [d,e]
ADD LOSARTAN 50 mg ONCE DAILY

Step 3: Review after 1 month, if treatment target not met [d]
ADD HYDROCHLOROTHIAZIDE 12.5 mg ONCE DAILY

Step 4: Review after 1 month, if treatment target not met [d]
REFER TO A SPECIALIST

THIS PROTOCOL SHOULD NOT BE USED FOR PATIENTS WHO *ARE* PREGNANT

IF PREGNANT, REFER TO SPECIALIST [f]

DO NOT PRESCRIBE LOSARTAN (OR ANY ACE-I OR ARBs) to women who *may* become pregnant.

For women of childbearing age who *may* become pregnant:
- Start amlodipine 2.5 – 5 mg once daily
- Review after one month. If treatment target not met, increase amlodipine to 10 mg once daily [d]
- Review after one month, if treatment target not met, REFER [d]

a. Refer to 'Protocol for Integrated Management of Hypertension, Diabetes and Cholesterol using **'Total Cardiovascular Risk Approach'** for **making decision** to treat hypertension in patients **aged ≥ 40 years.**
b. This protocol may also be used to treat patients below 40 years of age when treatment for hypertension is indicated.
c. Consider Amlodipine 2.5 mg as starting dose for patients aged ≥ 60 years and/ or patients with low body weight (BMI ≤ 18.5).
d. Check that patient has been taking drugs regularly and correctly, prior to increasing dose.
e. Before initiating and several weeks after starting losartan, check serum creatinine and serum potassium. **Avoid losartan for women of childbearing age who may become pregnant.**
f. If treatment for hypertension is to be initiated for pregnant women before referring her to specialist, consider methyldopa 125 – 250 mg twice daily

**Treatment target based on CVD risk based management**	
Most patients	≤ 140/90 mmHg
Having comorbidity: diabetes, ischaemic heart disease, stroke, chronic kidney disease	≤ 130/80 mmHg
High CVD Risk >30% (as per CVD risk prediction chart)	≤ 130/80 mmHg

LILFESTYLE MODIFICATION ADVICE FOR ALL PATIENTS

- Stop all tobacco use, avoid secondhand tobacco smoke.
- Stop taking alcohol.
- Increase physical activity to equivalent of brisk walk 150 minutes per week.
- If overweight, lose weight.
- Eat heart-healthy diet:
  - Reduce dietary salt intake.
  - Eat ⩾ 5 servings of vegetables/fruit per day.
  - Use healthy oils, such as sesame (Til), olive, safflower, sunflower.
  - Eat nuts, peas, whole grains and foods rich in potassium like spinach, watermelon, yogurt and banana.
  - Limit red meat to once or twice a week at most.
  - Eat fish or other food rich in omega 3 fatty acids at least twice a week.
  - Avoid added sugar from sweets, cakes, cookies, fizzy drinks, sugar sweetened beverages

For peer review only - http://bmjopen.bmj.com/site/about/guidelines.xhtml

Bangladesh National Protocol for Management of Type 2 Diabetes at Primary Health Care Level

Test patients who have symptoms of diabetes. Test adults who are 40+ years old. Test pregnant women during antenatal checkup

Diabetes diagnosed as per diagnostic criteria
Advise on diet, physical exercise and healthy lifestyle
Goal for glycaemic control: Fasting plasma glucose 4.4 – 7.2 mmol/L; Postprandial plasma glucose ≤ 10 mmol/L; Glycated haemoglobin ≤ 7%

Fasting Plasma Glucose (FPG) 7.0 – 10.0 mmol/L, or<br>Random Plasma Glucose (RPG) 11.1 – 12.0 mmol/L, or<br>Glycated Haemoglobin (A1C) 6.5 - 8.5%	FPG 10.1 – 14 mmol/L, or<br>RPG 12.1 – 18 mmol/L, or<br>A1C 8.6 – 10.0%	FPG > 14 mmol/L, or<br>RPG > 18 mmol/L, or<br>A1C > 10.0%
Counsel on diet and physical activity<br>↓<br>Review in 1 month.<br>If glycaemic goal not achieved:<br>Initiate with METFORMIN 250 – 500 mg once daily. *<br>Counsel on diet, physical activity and adherence to treatment at all visits.<br>↓<br>Review in 1 month.<br>If goal not achieved, increase dose of METFORMIN to 500 mg twice daily<br>↓<br>Review in 1 month.<br>If goal not achieved, increase dose of METFORMIN to 1000 mg twice daily<br>↓<br>Review in 3 month.<br>If goal not achieved, *ADD* GLICLAZIDE, 40 mg once a day<br>Counsel on hypoglycemia at all subsequent visits<br>↓<br>Review in one month.<br>If goal not achieved, increase dose of GLICLAZIDE by 40 mg every month till goal is achieved/ maximum dose of 80 mg twice daily is reached<br>↓	Start METFORMIN 500 mg twice daily +<br>GLICLAZIDE 40 mg twice daily. *<br>Counsel on diet, physical activity and adherence to treatment at all visits<br>↓<br>Review in 1 month.<br>If glycaemic goal not achieved, increase dose of METFORMIN to 1000 mg twice daily<br>↓<br>Review in 1 month.<br>If goal not achieved, increase dose of GLICLAZIDE by 40 mg every month, till 80 mg twice daily<br>↓	REFER to higher-level of care for starting insulin
Review in 3 months.<br>If goal not achieved, despite adherence to medication, healthy diet, and physical activity, REFER to higher-level of care for starting insulin		

* If serum creatinine is > 1.5 mg/ dl or there is contraindication/ intolerance to Metformin, start with Gliclazide 40 mg daily. Review and increase dose by 40 mg every month till goal is achieved/ maximum recommended dose is reached.

Supplementary Figure C. Consort diagram of the numbers of patients enrolled into care

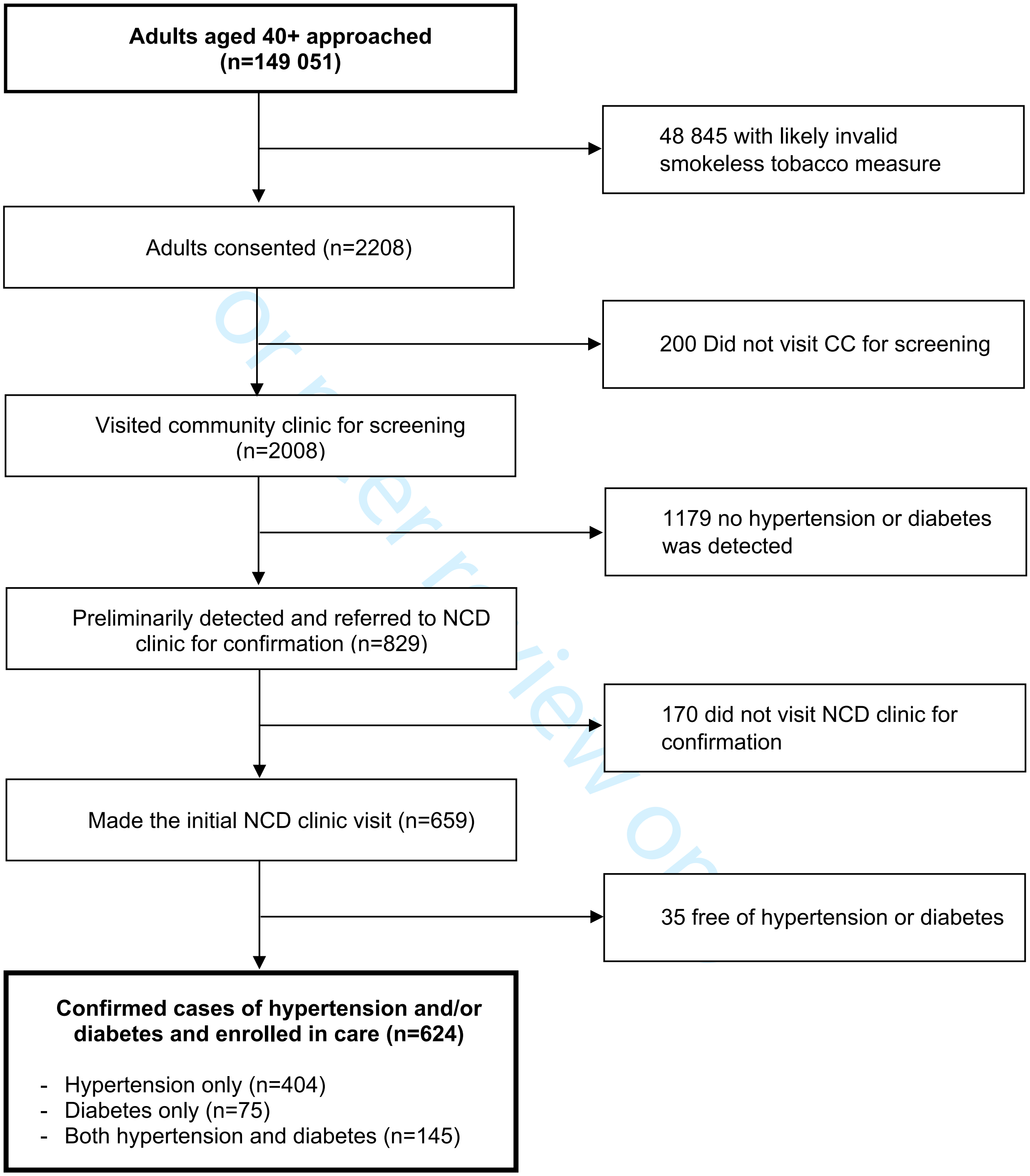

For peer review only - http://bmjopen.bmj.com/site/about/guidelines.xhtml

Supplementary Figure D. Change in mean blood pressure from baseline to follow-up visits *

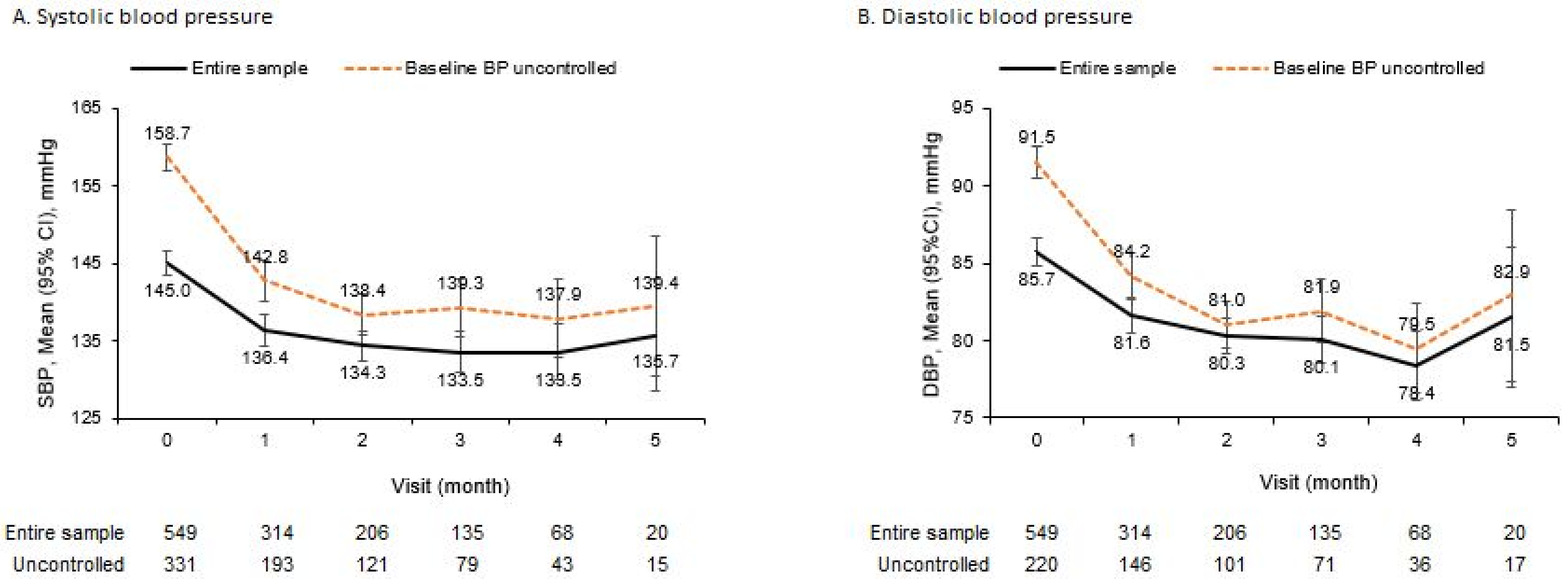

* BP, blood pressure; SBP, systolic blood pressure; DBP, diastolic blood pressure
Results adjusted age and sex.
Uncontrolled blood pressure at baseline was defined as systolic blood pressure ≥140 mmHg or diastolic blood pressure ≥90 mmHg.

For peer review only - http://bmjopen.bmj.com/site/about/guidelines.xhtml

Supplementary Figure E. Number of patient-visits per month occurred in NCD clinic and community clinic by types of tasks

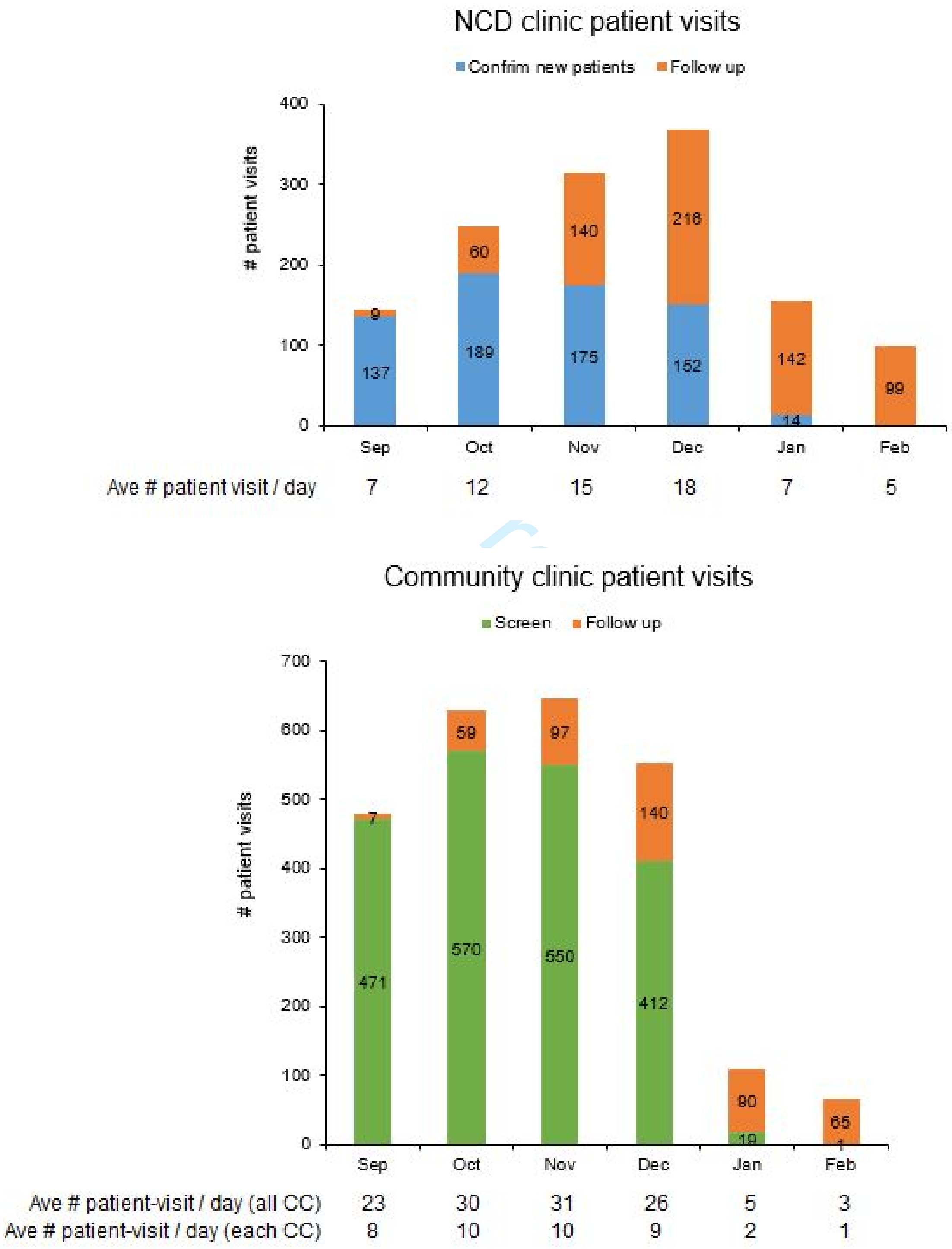

Supplementary Table 1. Change in mean blood pressure from baseline to follow-up visits

Visit *	Systolic blood pressure		Diastolic blood pressure	
	Entire sample	Baseline uncontrolled cases †	Entire sample	Baseline uncontrolled cases †
1 month	-8.0 (-10.2, -5.9)	-15.2 (-17.8, -12.5)	-3.9 (-5.0, -2.7)	-7.2 (-8.7, -5.8)
2 months	-9.7 (-12.2, -7.3)	-19.7 (-22.7, -16.8)	-4.9 (-6.3, -3.6)	-10.2 (-11.9, -8.5)
3 months	-10.6 (-13.6, -7.2)	-18.8 (-22.8, -14.8)	-5.3 (-6.9, -3.6)	-9.5 (-11.7, -7.3)
4 months	-10.6 (-14.4, -6.8)	-19.9 (-24.8, -15.0)	-7.0 (-9.3, -4.6)	-11.8 (-14.7, -8.8)
5 months	-8.5 (-16.0, -0.8)	-18.3 (-27.5, -9.1)	-3.9 (-8.5, 0.8)	-8.3 (-14.1, -2.5)
Constant	126.7 (119.5, 134.1)	160.5 (150.7, 170.4)	77.2 (72.5, 81.8)	93.4 (86.5, 100.2)
N	549	331	549	331

* All models adjusted for age (40-49, 50-59, 60-69, 70-100), sex (male, female), education (no formal education, primary or lower, secondary or higher), religion (Islam, Hindu), BMI category (under, normal, overweight or obese), community clinics (Khorakhai, Manmathapur, Jahanabad).
† Baseline uncontrolled cases were defined as systolic blood pressure ≥ 140 mmHg or diastolic blood pressure ≥ 90 mmHg

For peer review only

Supplementary Table 2. Factors associated with loss to follow-up

	LFU in the first visit	LFU over entire period
**Predictor**	IRR‡ (95% CI)	IRR‡ (95% CI)
Community clinic		
Khorakhai	1.00	1.00
Jahanabad	1.45 (1.02, 2.04)	1.21 (0.84, 1.74)
Manmathapur	1.58 (1.13, 2.22)	1.70 (1.22, 2.38)
Chronic conditions		
Both	1.00	1.00
Hypertension only	1.89 (1.31, 2.72)	1.85 (1.27, 2.70)
Diabetes only	1.27 (0.76, 2.14)	1.60 (0.96, 2.66)
Hypertension condition		
Known case, uncontrolled	1.00	1.00
Known case, controlled	1.42 (0.98, 2.06)	1.55 (1.07, 2.26)
New case	1.70 (1.20, 2.42)	1.48 (1.01, 2.15)
Religion		
Hindu	1.00	1.00
Islam	0.45 (0.32, 0.63)	0.85 (0.54, 1.32)

LFU, loss to follow up; IRR, incident rate ratio
Loss to follow-up over the entire study period is defined as no consultation recorded for two months or more.
IRRs derived from separate discrete time Poisson models controlling for age and sex, a logarithm exposure time were incorporated; only variables showing statistically significant associations were presented.

For peer review only - http://bmjopen.bmj.com/site/about/guidelines.xhtml

Supplementary Table 3. Change in mean blood pressure from baseline to follow-up visits - sensitivity check for attrition and composition [*]

Visit	Original results	IPW weighting [‡]	Restricting to patients with 3+ follow-up visits
**Uncontrolled blood pressure [§]**			
1 month	0.63 (0.55, 0.72)	0.65 (0.57, 0.75)	0.63 (0.51, 0.78)
2 months	0.48 (0.39, 0.60)	0.49 (0.39, 0.61)	0.48 (0.36, 0.64)
3 months	0.44 (0.33, 0.57)	0.43 (0.32, 0.57)	0.44 (0.33, 0.59)
4 months	0.49 (0.35, 0.69)	0.48 (0.34, 0.68)	0.50 (0.35, 0.71)
5 months	0.64 (0.32, 1.25)	0.60 (0.29, 1.24)	0.65 (0.33, 1.27)
N	549	549	135
**Uncontrolled blood glucose [¶]**			
1 month	0.69 (0.58, 0.82)	0.71 (0.59, 0.85)	0.63 (0.49, 0.80)
2 months	0.66 (0.54, 0.81)	0.67 (0.53, 0.83)	0.59 (0.45, 0.77)
3 months	0.60 (0.45, 0.78)	0.62 (0.47, 0.82)	0.55 (0.41, 0.72)
4 months	0.54 (0.36, 0.80)	0.56 (0.37, 0.85)	0.47 (0.30, 0.72)
5 months	0.44 (0.20, 0.96)	0.42 (0.18, 0.95)	0.40 (0.18, 0.89)
N	220	220	71

[*] All models adjusted for age (40-49, 50-59, 60-69, 70-100), sex (male, female), education (no formal education, primary or lower, secondary or higher), religion (Islam, Hindu), BMI category (under, normal, overweight or obese), community clinics (Khorakhai, Manmathapur, Jahanabad).

[‡] The inverse probability weighting approach weighted the analysis by the inverse of the predicted probability of dropping out from the study. A conditional logit model with the same set of covariates described above were used to predict the probabilities.

[§] Uncontrolled blood pressure was defined as systolic blood pressure ≥ 140 mmHg or diastolic blood pressure ≥ 90 mmHg

[¶] Uncontrolled blood glucose was defined as fasting plasma glucose ≥ 7.0 mmol/L or random plasma glucose ≥ 11.1 mmol/L

only

Reporting checklist for quality improvement in health care.

Based on the SQUIRE guidelines.

Instructions to authors

Complete this checklist by entering the page numbers from your manuscript where readers will find each of the items listed below.

Your article may not currently address all the items on the checklist. Please modify your text to include the missing information. If you are certain that an item does not apply, please write "n/a" and provide a short explanation.

Upload your completed checklist as an extra file when you submit to a journal.

In your methods section, say that you used the SQUIREreporting guidelines, and cite them as:

Ogrinc G, Davies L, Goodman D, Batalden P, Davidoff F, Stevens D. SQUIRE 2.0 (Standards for QUality Improvement Reporting Excellence): revised publication guidelines from a detailed consensus process

		Reporting Item	Page Number
**Title**			
	#1	Indicate that the manuscript concerns an initiative to improve healthcare (broadly defined to include the quality, safety, effectiveness, patientcenteredness, timeliness, cost, efficiency,	1

For peer review only

		and equity of healthcare)	
**Abstract**			
	#02a	Provide adequate information to aid in searching and indexing	3
	#02b	Summarize all key information from various sections of the text using the abstract format of the intended publication or a structured summary such as: background, local problem, methods, interventions, results, conclusions	3
**Introduction**			
Problem description	#3	Nature and significance of the local problem	6,7
Available knowledge	#4	Summary of what is currently known about the problem, including relevant previous studies	6,7
Rationale	#5	Informal or formal frameworks, models, concepts, and / or theories used to explain the problem, any reasons or assumptions that were used to develop the intervention(s), and reasons why the intervention(s) was expected to work	6,7
Specific aims	#6	Purpose of the project and of this report	8
**Methods**			
Context	#7	Contextual elements considered important at the outset of introducing the intervention(s)	8,9
Intervention(s)	#08a	Description of the intervention(s) in sufficient detail that others could reproduce it	10-13

For peer review only

Intervention(s)	#08b	Specifics of the team involved in the work	10-13
Study of the Intervention(s)	#09a	Approach chosen for assessing the impact of the intervention(s)	14
Study of the Intervention(s)	#09b	Approach used to establish whether the observed outcomes were due to the intervention(s)	15
Measures	#10a	Measures chosen for studying processes and outcomes of the intervention(s), including rationale for choosing them, their operational definitions, and their validity and reliability	14
Measures	#10b	Description of the approach to the ongoing assessment of contextual elements that contributed to the success, failure, efficiency, and cost	14,15
Measures	#10c	Methods employed for assessing completeness and accuracy of data	14,15
Analysis	#11a	Qualitative and quantitative methods used to draw inferences from the data	15,16
Analysis	#11b	Methods for understanding variation within the data, including the effects of time as a variable	15,16
Ethical considerations	#12	Ethical aspects of implementing and studying the intervention(s) and how they were addressed, including, but not limited to, formal ethics review and potential conflict(s) of interest	14
**Results**			
	#13a	Initial steps of the intervention(s) and their evolution over time	16,17

For peer review only

		(e.g., time-line diagram, flow chart, or table), including modifications made to the intervention during the project	
	#13b	Details of the process measures and outcome	18
	#13c	Contextual elements that interacted with the intervention(s)	18,19
	#13d	Observed associations between outcomes, interventions, and relevant contextual elements	17,18
	#13e	Unintended consequences such as unexpected benefits, problems, failures, or costs associated with the intervention(s).	18,19
	#13f	Details about missing data	17
**Discussion**			
Summary	#14a	Key findings, including relevance to the rationale and specific aims	19
Summary	#14b	Particular strengths of the project	21
Interpretation	#15a	Nature of the association between the intervention(s) and the outcomes	19-21
Interpretation	#15b	Comparison of results with findings from other publications	19-21
Interpretation	#15c	Impact of the project on people and systems	19-21
Interpretation	#15d	Reasons for any differences between observed and anticipated outcomes, including the influence of context	19-21
Interpretation	#15e	Costs and strategic trade-offs, including opportunity costs	19-21
Limitations	#16a	Limits to the generalizability of the work	21

For peer review only

Limitations	#16b	Factors that might have limited internal validity such as confounding, bias, or imprecision in the design, methods, measurement, or analysis	21
Limitations	#16c	Efforts made to minimize and adjust for limitations	21
Conclusion	#17a	Usefulness of the work	22
Conclusion	#17b	Sustainability	22
Conclusion	#17c	Potential for spread to other contexts	22
Conclusion	#17d	Implications for practice and for further study in the field	22
Conclusion	#17e	Suggested next steps	22
**Other information**			
Funding	#18	Sources of funding that supported this work. Role, if any, of the funding organization in the design, implementation, interpretation, and reporting	2

None The SQUIRE 2.0 checklist is distributed under the terms of the Creative Commons Attribution License CC BY-NC 4.0. This checklist can be completed online using https://www.goodreports.org/, a tool made by the EQUATOR Network in collaboration with Penelope.ai

For peer review only

BMJ Open

Enhancing Care Quality and Accessibility through Digital Technology-Supported Decentralization of Hypertension and Diabetes Management: A Proof-of-Concept Study in Rural Bangladesh

Journal:	*BMJ Open*
Manuscript ID	bmjopen-2023-073743.R1
Article Type:	Original research
Date Submitted by the Author:	17-Jul-2023

Complete List of Authors:	Xie, Wubin; Nanyang Technological University<br>Paul, Rina Rani; BRAC University James P Grant School of Public Health, Centre for Non-communicable Diseases and Nutrition<br>Goon, Ian; UNSW, Tyree Foundation Institute of Health Engineering; Baker Heart and Diabetes Institute<br>Anan, Aysha; BRAC University James P Grant School of Public Health, Centre for Non-communicable Diseases and Nutrition<br>Rahim, Aminur; Sprightly Pte Ltd<br>Hossain, Md Mokbul; BRAC University, Centre for Non-communicable Diseases and Nutrition<br>Hersch, Fred; Google Health<br>Oldenburg, B; Baker Heart and Diabetes Institute<br>Chambers, John; Nanyang Technological University; Imperial College London, Department of Epidemiology and Biostatistics<br>Mridha, Malay Kanti; BRAC University James P Grant School of Public Health, Centre for Non-communicable Diseases and Nutrition

**Primary Subject Heading**:	Global health
Secondary Subject Heading:	Health services research, Public health
Keywords:	Hypertension < CARDIOLOGY, DIABETES & ENDOCRINOLOGY, Health Services Accessibility

SCHOLARONE™ Manuscripts

I, the Submitting Author has the right to grant and does grant on behalf of all authors of the Work (as defined in the below author licence), an exclusive licence and/or a non-exclusive licence for contributions from authors who are: i) UK Crown employees; ii) where BMJ has agreed a CC-BY licence shall apply, and/or iii) in accordance with the terms applicable for US Federal Government officers or employees acting as part of their official duties; on a worldwide, perpetual, irrevocable, royalty-free basis to BMJ Publishing Group Ltd ("BMJ") its licensees and where the relevant Journal is co-owned by BMJ to the co-owners of the Journal, to publish the Work in this journal and any other BMJ products and to exploit all rights, as set out in our licence.

The Submitting Author accepts and understands that any supply made under these terms is made by BMJ to the Submitting Author unless you are acting as an employee on behalf of your employer or a postgraduate student of an affiliated institution which is paying any applicable article publishing charge ("APC") for Open Access articles. Where the Submitting Author wishes to make the Work available on an Open Access basis (and intends to pay the relevant APC), the terms of reuse of such Open Access shall be governed by a Creative Commons licence – details of these licences and which Creative Commons licence will apply to this Work are set out in our licence referred to above.

Other than as permitted in any relevant BMJ Author's Self Archiving Policies, I confirm this Work has not been accepted for publication elsewhere, is not being considered for publication elsewhere and does not duplicate material already published. I confirm all authors consent to publication of this Work and authorise the granting of this licence.

For peer review only

Enhancing Care Quality and Accessibility through Digital Technology-Supported Decentralization of Hypertension and Diabetes Management: A Proof-of-Concept Study in Rural Bangladesh

Wubin Xie;[1] Rina Rani Paul; [2] Ian Goon;[3,4] Aysha Anan;[2] Aminur Rahim;[4] Md Mokbul Hossain;[2] Fred Hersch;[5] Brian Oldenburg;[4,7] John Chambers;[1,8*] Malay Kanti Mridha[2*]

[1] Lee Kong Chian School of Medicine, Nanyang Technological University, Singapore

[2] Centre for Non-communicable Diseases and Nutrition, BRAC James P Grant School of Public Health, Dhaka, Bangladesh

[3] Tyree Foundation Institute of Health Engineering, UNSW Sydney, NSW 2052, Australia

[4] Baker Heart and Diabetes Institute, Melbourne, Australia

[4] Sprightly Pte Ltd, Singapore

[5] Google Health, Palo Alto, CA, USA

[7] School of Psychology and Public Health, La Trobe University, Melbourne, Australia

[8] Department of Epidemiology and Biostatistics, School of Public Health, Imperial College London, London, UK

*** Corresponding authors:**

Prof John Chambers
E-mail: john.chambers@ntu.edu.sg
Tel: (65) 6592 3938

Prof Malay Kanti Mridha
E-mail: malay.mridha@bracu.ac.bd
Tel: (880) 2-48812213-18

Word count: 4458

For peer review only - http://bmjopen.bmj.com/site/about/guidelines.xhtml

Abstract

Objective The critical shortage of healthcare workers, particularly in rural areas, is a major barrier to quality care for noncommunicable disease (NCD) in low- and middle-income countries. In this proof-of-concept study, we aimed to test a decentralized model for integrated diabetes and hypertension management in rural Bangladesh to improve accessibility and quality of care.

Design and setting The study is a single cohort proof-of-concept study. The key interventions comprised shifting screening, routine monitoring, and dispensing of medication refill from a doctor-managed subdistrict NCD clinic to non-physician health worker managed village level community clinics; a digital care coordination platform was developed for electronic health record, point-of-care support, referral, and routine patient follow-up. The study was conducted in Parbatipur subdistrict, Rangpur Division, Bangladesh.

Participants A total of 624 participants were enrolled in the study (mean[sd] age, 59.5 [12.0]; 65.1% female).

Outcomes Changes in blood pressure and blood glucose control, patient retention, and patient-visit volume at the NCD clinic and community clinics.

Results The proportion of patients with uncontrolled blood pressure reduced from 60% at baseline to 26% at the third month of follow-up, a 56% (IRR 0.44; 95% CI 0.33, 0.57) reduction after adjustment for covariates. The proportion of patients with uncontrolled blood glucose decreased from 74% to 43% at the third month of follow-up. Attrition rates

For peer review only - http://bmjopen.bmj.com/site/about/guidelines.xhtml

immediately after baseline and during the entire study period were 29.1% and 36.2%, respectively.

Conclusion The proof-of-concept study highlights the potential for involving lower-level primary care facilities and non-physician health workers to rapidly expand much needed services to the patients with hypertension and diabetes in Bangladesh, and in similar global settings. Further investigations are needed to evaluate the effectiveness of decentralized hypertension and diabetes care.

For peer review only

For peer review only - http://bmjopen.bmj.com/site/about/guidelines.xhtml

Strength and limitation of the study

- The study is among the first to evaluate a digital technology-supported, decentralized primary care model for integrated diabetes and hypertension management in a resource-constrained setting.
- Generated important preliminary effectiveness data on a decentralized model to rapidly expand primary care services to people with hypertension and diabetes.
- Enabled by digital tools, rich longitudinal data were collected to evaluate patient treatment outcome and retention.
- Duration of the study is short, and generalizability is limited by a local sample drawn from a subdistrict in northern Bangladesh.

For peer review only

For peer review only - http://bmjopen.bmj.com/site/about/guidelines.xhtml

Introduction

Cardiovascular disease (CVD) and type 2 diabetes are major causes of premature death and disability. An estimated 10.5% (or 536.6 million) adults are living with diabetes, CVD caused 18.6 million deaths worldwide in 2019.[1] Close to 80% of CVD deaths occurred in low- and middle-income countries (LMICs).[2, 3] The number of people with hypertension, a highly prevalent, major risk factor for CVD, doubled from 1990 to 2019.[4] Complications of persistently elevated blood pressure and poor glycaemic control, such as heart disease, stroke, chronic kidney disease, blindness, pose enormous socioeconomic burdens on individuals, families, and healthcare systems, contributing to poverty, inequality and social instability.[5] Despite the availability of effective and inexpensive medications and other management options for diabetes and hypertension for many years, treatment and control rates of these conditions remain unacceptably low in LMICs, particularly in South Asia.[6, 7] Recent estimates indicate that in South Asia, only 26% men and 37% women with hypertension were on treatment, and control rates were only 11% and 17% for men and women respectively.[4]

Traditionally designed to provide basic episodic care with an emphasis on maternal and child health and infectious diseases, primary healthcare systems in LMICs are under resourced and ill-prepared for the surging burden of CVD and other noncommunicable diseases (NCD).[8] The critical shortage of healthcare workers, particularly in rural areas, is a major barrier to quality care.[9] Most evidently in grassroot level facilities, primary care providers are untrained and unequipped to address primary and secondary prevention of NCD.[10, 11] As a result, NCD care is

For peer review only - http://bmjopen.bmj.com/site/about/guidelines.xhtml

poorly accessible with primary care systems.[12] Long waiting time and high costs associated with travel and lost wages discourage people from seeking care from qualified providers.[13-16]

Although few examples exist for hypertension and diabetes care, decentralization with task shifting (sharing) in HIV care has been identified as a viable strategy to rapidly scale-up antiretroviral therapy in resource-limited settings.[17-19] In this model of care, stable patients are transferred through a ‘down-referral’ system from the doctor-managed clinic where they initiated care to non-physician health worker managed local primary health clinics for continued monitoring and treatment. Decentralized care improves geographic accessibility, reduces costs associated with care seeking, and enables the health system to serve a much larger patient population.[17] Local health service providers could monitor patients more effectively and efficiently, and to form a personal relationship that strengthens effective communication and counselling for treatment management.[20] People with HIV/AIDS managed locally are less likely to be lost to follow-up and more satisfied with care, compared with those managed in centralized clinics.[21]

Digital technologies offer the potential to improve access to and quality of healthcare in LMICs.[22] Digital tools have been tested in supporting quality improvement in hypertension and diabetes management in South Asian settings. Several pilot studies have documented the utility of digital tools to facilitate protocol-based algorithmic care through clinical decision support, and to promote continuity and coordination of patient visits across different healthcare settings, although findings are somewhat mixed.[23-25]

For peer review only - http://bmjopen.bmj.com/site/about/guidelines.xhtml

Little is known whether decentralizing routine hypertension and diabetes services to lower-level primary care facilities and non-physician health workers is beneficial for enhancing care quality and accessibility in resource-poor settings, and how digital technology can be harnessed to support the processes. In the present proof-of-concept study, we aimed to 1) generate preliminary data on the effectiveness of a novel digital technology supported decentralization of primary care diabetes and hypertension management in a rural subdistrict in Northern Bangladesh, and 2) to understand its potential future implications for the patient care pathway, the patient load in primary care facilities at a subdistrict and village level, and patient retention.

Methods

Context

Like many other LMICs, Bangladesh is experiencing rapid demographic and epidemiologic transitions. In the recent years, CVDs replaced infectious disease and maternal and neonatal conditions as the leading cause of death in Bangladesh.[26] The prevalence of hypertension and diabetes are high and continue to increase in recent years.[27] The 2017-2018 Bangladesh Demographic and Health Survey showed a 9.8% prevalence of diabetes and a 27.4% prevalence of hypertension among adult population.[28, 29] Treatment and control rates remained low. Only 31.1% adults with hypertension and 28.2% adults with diabetes were on treatment, and merely 12% achieved blood pressure control and 7% had controlled diabetes.[29, 30]

Bangladesh has a three-tiered (subdistrict, union, ward) primary healthcare system in rural areas (Figure 1). The subdistrict level Upazila Health Complexes,

For peer review only - http://bmjopen.bmj.com/site/about/guidelines.xhtml

typically 50-bed hospitals, are at the top of the primary care system, serving a population of three to four hundred thousand. Community clinics are the most peripheral health care facilities and the first point of contact of public healthcare system to rural residents. With two rooms and drinking-water and lavatory facilities under a covered waiting area, community clinics were designed to be accessible for more than 80% of rural population within less than 30-minute walking distance. Staffed by a Community Healthcare Provider with support from a few community health workers, community clinics have played critical roles in reducing malnutrition and child mortality, and improving maternal and child health.[31]

Since 2011, the Government of Bangladesh has promoted improved hypertension and diabetes care through national multisectoral actions, most notably through the establishment of dedicated noncommunicable disease related healthcare delivery points ("NCD Corners") at subdistrict hospitals.[32] Although originally planned to involve lower-level primary care facilities in the delivery of NCD care, hypertension and diabetes care are largely unavailable at village level.[33]

Study settings

This proof-of-concept study was implemented in a newly initiated study NCD clinic at Parbatipur subdistrict, in collaboration with three community clinics at Khorakhai, Jahanabad, and Manmathapur. Parbatipur is a subdistrict of Dinajpur district, in Rangpur Division of northern Bangladesh. Parbatipur had a population of 365,103 in 2011. Public primary health care system in Parbatipur consists of one Upazila health complex (subdistrict hospital), 10 union health (sub)centres, and 34 community clinics.[34] With a maximum capacity of seeing 40-50 patients per day, the

For peer review only - http://bmjopen.bmj.com/site/about/guidelines.xhtml

NCD Corner at the Parbatipur upazila health complex manages a few hundred diabetes and hypertension patients – a very small fraction of the estimated patient population with these conditions.

Study design

The study is a single cohort proof-of-concept study. Decentralized hypertension and diabetes care comprised screening, routine monitoring, and dispensing of medication refill at village level community clinics and treatment initiation and titration at subdistrict level NCD clinic. Patient flow in this model of care included community health workers motivate adults aged 40 years and above to get screening for hypertension and diabetes at community clinics; once confirmed, patients received initial care at the study NCD clinic and subsequent follow-up at community clinics. At the follow-up visits, community healthcare providers perform a basic physical examination (i.e., body weight and height measurement, calculation of BMI, measure blood pressure and blood glucose measurement), inquire about any new symptoms, assessed CVD risk, emphasize the importance of medication adherence, provide advice regarding a healthy lifestyle, and refill prescribed medications. Patients were referred back to NCD clinic for medication titration and further evaluation if treatment target was not met or any urgent symptoms or conditions were recorded.

The study NCD clinic was staffed by one medical doctor and one paramedic. Both the doctor and the paramedic received a four-day training on hypertension and diabetes management following the package on essential NCD interventions of the World Health Organization. The paramedic had one year of post-graduate medical

For peer review only - http://bmjopen.bmj.com/site/about/guidelines.xhtml

education. The doctor carried out physiological examination and provided diagnostic and treatment services according to the Bangladesh National Protocol for Management of Diabetes and Hypertension, adopted from the World Health Organization Package of essential non-communicable disease interventions.[35] The paramedic was responsible for taking medical history, measurement of blood pressure and blood glucose, delivery of health education, counselling, and medication disbursement.

One Community Healthcare Provider was employed to provide services in three community clinics (2 days per week in each community clinic). The Community Healthcare Provider was supported by three newly recruited NCD community health workers (one for each community clinic). The Community Healthcare Provider received a 1-year post-secondary education medical training. Community health workers were newly recruited from local communities with a minimum of secondary school education. The Community Healthcare Provider and the community health workers received a 7-day initial training on behavioural risk factors of hypertension and diabetes, anthropometry, lifestyle counselling and follow-up, and received ongoing regular (weekly to monthly) supervision from the study NCD clinic medical staff and the local study investigators.

The Community Healthcare Provider and community health workers were responsible for administering written informed consent, registering participants, referring adults for screening, follow-up, medication adherence, health education/counselling in the community. They were regularly supervised by the medical doctor from the study NCD clinic and by the study investigators. The study NCD clinic and community clinics were equipped with the necessary medical, pharmaceutical, and logistical supplies and

For peer review only - http://bmjopen.bmj.com/site/about/guidelines.xhtml

equipment. Electronic health records were maintained by the study digital care coordination platform.

Patient screening and eligibility criteria

Adults aged 40 and above were approached at their households and registered and referred by community health workers to be screened at the community clinics. Those with a history of hypertension or diabetes, and who screened positive for hypertension or diabetes were referred to the study NCD clinic for further evaluation. The WHO non-laboratory-based CVD risk scores were calculated.[35] Existing and new patients with hypertension and/or diabetes were enrolled. Diabetes was diagnosed if any of the following criteria was met: 1) participant reported taking medications for diabetes; 2) fasting plasma glucose (FPG) ≥ 7.0 mmol/L or random plasma glucose (RPG) ≥ 11.1 mmol/L with symptom of diabetes at one visit; 3) FPG ≥ 7.0 mmol/L or RPG ≥ 11.1 mmol/L at two separate visits, at least one day apart. Hypertension was diagnosed if any one of the following criteria was met: 1) participant reported taking medications for hypertension; 2) average systolic blood pressure (SBP) ≥160 mmHg and/or diastolic blood pressure (DBP) ≥100 mmHg on a single visit; 3) average SBP ≥130 mmHg and/or DBP ≥80 mmHg on a single visit and participant had a high risk of CVD (10-year CVD risk score ≥ 0.3) or participant reported a diagnosis of diabetes; 4) average SBP ≥140 mmHg and/or DBP ≥90 mmHg on two separate visit, at least one day apart. The diagnostic criteria were consistent with the Bangladesh National Protocol for Management of Diabetes and Hypertension.

For peer review only - http://bmjopen.bmj.com/site/about/guidelines.xhtml

Pregnant women and patients with life-threatening conditions were excluded from the study. Patients with very high blood pressure or those with acute symptoms of cardiovascular events were referred immediately to tertiary hospitals as per national protocol and were excluded from the analyses. Treatment decision and patient management followed the Bangladesh national protocol for integrated management of hypertension, diabetes, and cholesterol using a total cardiovascular risk approach (supplementary Figure A).

Digital platform for care coordination

A digital care coordination platform was developed to address challenges in delivering guideline-based decentralized care in primary healthcare settings. A detailed description of development and deployment of the digital system is available in **supplementary materials** (*The development and deployment of a digital platform for primary care hypertension and diabetes care coordination in Bangladesh, Supplementary Figures B, C, and D*). Briefly, the digital platform was developed in close collaboration with primary health care providers and policymakers through multiple stakeholder meetings and with extensive user-centric research to understand user needs. The system comprised an Android tablet-based application with dashboards to support task shifting/sharing of registering, screening, counselling, and routine patient management. The application allowed community health workers and Community Healthcare Providers to enter patient data, which were accessible to healthcare providers at subdistrict level NCD clinic. With built-in algorithms, the application flagged behavioural risk factors, prompts counselling requirements, highlights elevated levels of blood pressure/glucose, and generated referral recommendations for confirmatory

For peer review only - http://bmjopen.bmj.com/site/about/guidelines.xhtml

diagnosis and treatment. At the study NCD clinic, a paramedic performed additional examinations and updated patients' data. The digital care coordination platform generated CVD risk scores based on WHO cardiovascular disease risk non-laboratory-based charts (South Asia) and had all pertinent information needed by the medical doctor at the NCD clinic to make treatment decisions and management plans, which were then incorporated into the patient record. Patient dashboards were continuously updated, enabling healthcare providers at community clinics to follow up with routine examination, medication refill, and counselling.

Data collection and measurements

The present study relied on routinely collected data through the digital care coordination platform. Data were collected during screening and follow-up visits between 1 September 2021 and 28 February 2022. Self-reported sociodemographic characteristics (age, sex, education, religion), health behaviours (tobacco use, physical activity, diet), medical history (diabetes, CVD, chronic kidney disease, respiratory diseases, cancer) were collected by interviewer administered questionnaire installed on tablets. Height was measured with stadiometers (Infant Child Adult ShorrBoard, Weight and Measure LLC), and weight was measured with a digital weighing machine (Tanita UM70). Blood pressure was measured with automatic digital monitor (Omron HEM 7120) following standard operating procedures. Capillary blood was used to measure fasting/random blood glucose with glucometers (Accu-Chek ® Active).

The primary outcome measurements were changes in proportion of people with uncontrolled conditions from baseline to follow-up. Patients were considered to have uncontrolled hypertension if they had SBP≥140 mmHg and DBP≥90 mmHg, and to

For peer review only - http://bmjopen.bmj.com/site/about/guidelines.xhtml

have uncontrolled diabetes if they had FPG≥7.0 mmol/L or RPG ≥ 11.1mmol/L. Mean changes in blood pressure and blood glucose from baseline were considered as secondary outcomes.

Patient retention was assessed as the incidence of drop out for patients enrolled. Drop-out was defined as no recorded visit for ≥ 2 months. Intervention-related adverse events were documented as safety measurements. This study was approved by the Institutional Review Board of BRAC University (IRB reference number 2020-009-IR). Written informed consent was obtained from all participants.

Data safety and monitoring

A purpose-built database was developed to facilitate collection, secure storage, and appropriate sharing of study data, including reporting of clinically relevant results to individual participants. The database was commissioned under the supervision of the Chief Investigator and respective site Principal Investigator. Personal and clinical data were separated by pseudonymization to enhance data security. Appropriate authentication and access control mechanisms were implemented. Data were stored securely both locally and in cloud-based infrastructure. All personal identifiers were removed before analysis.

Statistical analysis

Descriptive statistics stratified by patient type (hypertension only, diabetes only, or having both conditions) were reported. Changes in proportion of patients with an uncontrolled condition were analysed with generalized estimating equation (GEE) Poisson regression with robust variance. Follow-up visit was included as binary

For peer review only - http://bmjopen.bmj.com/site/about/guidelines.xhtml

indicators in the regression model to capture the change in blood pressure and blood glucose control relative to the baseline. GEE Poisson is widely used for repeated binary outcomes as it provides consistent estimates of the relative risk and is more stable than the GEE binomial model.[36, 37] Change in uncontrolled blood pressure was examined among those with a diagnosis of hypertension (with or without diabetes), while change in uncontrolled blood glucose was evaluated among those with a diagnosis of diabetes, alone or with hypertension. Models were adjusted for age (40-49, 50-59, 60-69, 70-100), sex (male, female), education (no formal education, primary or lower, secondary or higher), religion (Islam, Hindu), BMI category (<18.5, 18.5-22.9, ≥23 kg/m^2), and community clinic location (Khorakhai, Manmathapur, Jahanabad). The analyses were repeated for the subset patients who had uncontrolled hypertension or diabetes at enrolment.

As secondary outcomes, changes in SBP and DBP in each of the 5 visits from baseline were assessed with linear mixed effects models among those with a diagnosis of hypertension (with or without diabetes). Changes in mean fasting and random blood glucose were not assessed given the limited number of repeated measurements (patients had either FPG or RPG, not both). We assessed factors associated with loss to follow-up with Poisson models. A logarithm time to drop out was incorporated in incidence ratio estimation. Follow-up time was rounded to roughly one month interval to aid better interpretation. Data management and analyses were conducted using STATA MP v14. Statistical significance was determined by two-sided hypothesis tests ($p<0.05$).

Sensitivity analyses were conducted to assess potential bias introduced by sample attrition with inverse probability weighting. The inverse probability weighting

For peer review only - http://bmjopen.bmj.com/site/about/guidelines.xhtml

approach weighted the analysis by the inverse of the predicted probability of dropping out from the study. A conditional logit model with the same set of covariates described above was used to predict the probabilities. In addition, we restricted our analyses to patients with at least three follow-up visits to test whether the results were sensitive to better balancing sample composition for timing of enrolment and follow-up.

Patient and public involvement

Before the study, we conducted formative research including a qualitative assessment to explore technology readiness and acceptability of services among the health providers and a quantitative assessment of readiness of health facilities to deliver NCD services. Multiple meetings with primary care physicians, NCD nurses, community health workers, and local health authorities were organized to understand the current care pathway and to identify gaps in usual care. Informal discussions with patients regarding their experience with the current model of care were conducted. Patients were not involved in the analysis or interpretation of the data, nor did they contribute to the writing of the manuscript.

Results

Patient characteristics

Of the 2232 people approached, 2208 (98.9%) consented to participate in the study. Among those consented, 2008 people attended the screening of hypertension and diabetes at the community clinics, of whom 829 were found to have possible hypertension and/or diabetes and were referred to the study NCD clinic for further

For peer review only - http://bmjopen.bmj.com/site/about/guidelines.xhtml

evaluation (Supplementary Figure E). From the 829 referred, 659 attended the study NCD clinic, and amongst them, 624 were confirmed and included in the analytic sample (mean[sd] age, 59.5 [12.0]; 65.1% female, 45.0% had no formal education). There were 404 patients who had hypertension without diabetes, 75 patients who had diabetes without hypertension, 145 patients who had both. At the baseline, of 549 patients with hypertension, 331 (60.3%) had uncontrolled blood pressure, while 163 out of 220 (74.1%) patients with diabetes had uncontrolled glucose levels (**Table 1**).

Changes in blood pressure and blood glucose

Amongst the 549 patients with hypertension, the age- and sex-adjusted proportion of people with uncontrolled blood pressure decreased from 60% (95% CI, 56%, 64%) at baseline to 26% (95% CI, 19%, 33%) at the third month of follow-up (Figure 2), a 56% (IRR 0.44; 95% CI 0.33, 0.57) reduction in the probability of having uncontrolled blood pressure after adjustment for covariates (Table 2). The proportion of people with uncontrolled blood pressure rebounded to 39% (95% CI, 12%, 65%) at the fifth month of follow-up, and the difference was no longer statistically significant compared with the proportion at baseline (IRR 0.64; 95% CI, 0.32, 1.25), although the number of observations at the fifth visit (n=20) was notably smaller relative to baseline (n=549). Among the subset of people with uncontrolled blood pressure at baseline, 65% (IRR 0.35; 95% CI 0.26, 0.47) and a 63% (IRR 0.37; 95% CI 0.18, 0.76) achieved blood pressure control by the 3rd and 5th visit, respectively.

The mean age- and sex-adjusted SBP at baseline was 145.0 mmHg (95% CI, 143.4, 146.7) among the 549 people with hypertension. Those with uncontrolled BP had

For peer review only - http://bmjopen.bmj.com/site/about/guidelines.xhtml

higher mean SBP at baseline (158.7 mmHg; 95% CI, 157.0, 160.4) compared with the entire sample of hypertension patients (Supplementary Figure F). A substantial reduction in SBP was observed by the first follow-up visit (-8.0 mmHg; 95% CI, -10.2, -5.9) and the reduction sustained throughout the study period (-8.5 mmHg at 5 months; 95% CI, -16.0, -0.8, Supplementary Table A). The reduction in SBP was more pronounced among people with uncontrolled hypertension at baseline (first follow-up visit [-15.2 mmHg; 95% CI, -17.8, -12.5]; at the last follow-up visit [-18.3 mmHg; 95% CI, -27.5, -9.1]). Similar patterns of estimates were observed for DBP measures.

We observed a similar pattern of change in the control of blood glucose among the diabetics. At baseline, 74% (95% CI, 68%, 80%) of patient with diabetes had uncontrolled blood glucose. This proportion dropped to 43% (95% CI, 32%, 54%) at the third follow-up visit, and 31% (95% CI, 7.5%, 54.8%) at the fifth follow-up visit. The reduction in the proportion of patients with uncontrolled blood glucose was more pronounced among people with uncontrolled condition at baseline.

Patient retention, process evaluation, and sensitivity checks

The proportions of patients lost to follow-up immediately after baseline and during the entire study period were 29.1% and 36.2%, respectively (Figure 3). Factors associated with an increased probability of loss to follow-up included having a controlled condition, being a new patient, and only having hypertension at baseline (Supplementary Table B). Given the nature of continuous enrolment, patients recruited earlier had more follow-up data compared with those entered the study later.

For peer review only - http://bmjopen.bmj.com/site/about/guidelines.xhtml

In total, 41% of follow-up visits were carried out at the community clinics. Average daily numbers of patient-visits ranged from 5 – 18 for NCD clinic and 1 – 10 in each community clinic (Supplementary Figure G). Within the 6-month study period, we recorded no deaths, or serious intervention-related adverse events. The results appeared robust in sensitivity analyses incorporating inverse probability weighs for drop-out and excluding respondents with 2 or less follow-up visits (Supplementary Table C).

Discussion

In this study, we tested a digital technology-supported decentralization of diabetes and hypertension management in rural Bangladesh. The service delivery model with shifting routine hypertension and diabetes services to lowest level primary care facilities and non-physician health workers achieved a high treatment success rate and a relatively high patient retention. The results highlighted the potential of technology supported decentralized care to address critical shortage of health service providers and geographical barriers to access, along retention of patients with hypertension and diabetes.

Primary care delivery of hypertension and diabetes services in Bangladesh is hampered by a critical shortage of trained health workers and poor geographical accessibility. In Bangladesh, only about one in three patients with hypertension or diabetes are currently on treatment.[28, 29] The government NCD Corners at subdistrict level are remote and poorly accessible to a large body of population living with NCDs.[13] As a result, the majority of people with hypertension and diabetes are

For peer review only - http://bmjopen.bmj.com/site/about/guidelines.xhtml

For peer review only

dependent on the largely unregulated private sector, and frequently received services from untrained or unqualified care providers.[38]

Our preliminary results showed that with the support of a digital care coordination platform and proper training, non-physician health workers based in the community or community clinics can successfully deliver screening for hypertension and diabetes, routine follow-up, counselling, drug refill, and other aspects of NCD management. This approach of healthcare delivery has the potential to substantially improve access to healthcare and reduce the burden on the physician-managed NCD clinics. Overall, the proportion of patient follow-up visits carried out at community clinics was 41%. Routine patient follow-up at community location could be further enhanced by introducing telemedicine and dose titration by non-physician health workers. Through technology-supported decentralization and the involvement of the large number of peripheral primary care facilities, hypertension and diabetes services could be rapidly expanded in Bangladesh and similar global settings.

In addition to the lack of human resources, inconsistent adherence to treatment protocol among health care providers, poor patient management, and irregular follow-up are important obstacles for effective hypertension and diabetes management.[13, 39] As a result of these barriers, compounded by low levels of awareness and screening, the proportion of all patients with controlled condition is merely 7% for diabetes and 12% for hypertension in Bangladesh.[29, 30] A variety of digital tools have been examined for hypertension and diabetes care quality improvement in South Asian settings with mixed findings.[23, 24, 40, 41] Digital tools can be invaluable in strengthening health systems for NCD care delivery, however, they need to be an

For peer review only - http://bmjopen.bmj.com/site/about/guidelines.xhtml

integral part of an multifaceted intervention, with thoughtful consideration of care pathways and user interfaces, to exert effects within specific health system contexts.[6] Following international standards of interoperability throughout the designing phrase, the digital platform resources developed in the present study are open-source and can be readily utilized by the global health community.

Treatment adherence and retention are major challenges in NCD management, particularly in LMICs. [42-44] In a recent study of task shifting to non-physician clinicians for integrated management of hypertension and diabetes in rural Cameron, Labhardt and colleagues reported a retention rate of 18.1% among treated patient at one year. Another community health worker-led, technology-enabled private sector intervention for diabetes and hypertension management among urban poor in India observed a 44.0% retention rate at 6 months.[44] The observed retention rates of 63.8% by the end of the present study appeared high. While the reasons were not formally tested, possible explanations include the ease of obtaining medication refill, alleviated travel-related burden, and more counselling time with non-physician health workers.

Our findings showed several factors were associated with loss to follow-up, including being newly diagnosed, having a controlled condition at enrolment, and/or being diagnosed with hypertension only. In addition, the results showed a particularly high attrition rate immediately after enrolment, consistent with previous studies.[42, 44] Attrition incidence appeared to decrease with longer follow-up despite higher accumulative attrition rates over time, likely due to the depletion of individuals with high propensity of dropping out. These observations may have important implications for routine healthcare delivery to improve patient retention. Additional efforts may be

For peer review only - http://bmjopen.bmj.com/site/about/guidelines.xhtml

needed to emphasize the need for regular follow-up to newly diagnosed patients, those with controlled status at enrolment, and those detected with hypertension only, especially at enrolment.

As a proof-of-concept study, the project comprised an uncontrolled and unblinded evaluation of a decentralized service model, supported by a digital care coordination platform. Nevertheless, the observed rates of 61% for blood pressure control and 69% for blood glucose control at the end of the study compared favourably with existing implementation studies and clinical trials of multicomponent health system interventions in developing countries.[45-48] For example, a recent cluster-randomized controlled trail in Bangladesh, India, and Sri Lanka, a multifaceted intervention involving trained community health workers for home-based blood pressure monitoring and counselling achieved blood pressure control in 53.2% of the participants in the intervention group and 43.7% of the control group.[46] The findings will inform the design of our planned further studies to test the feasibility and utility of the interventions within government primary care system, as well as to evaluate the effectiveness of the digital technology supported decentralized care in Bangladesh.

Conclusion

In the proof-of-concept study, a digital technology supported decentralized care model achieved high levels of blood pressure and blood glucose control among patients with hypertension and diabetes in Bangladesh. Our results suggest that with proper training, supportive supervision, and access to digital technology, non-physician health workers at peripheral primary facilities can deliver health screening, counselling, routine

For peer review only - http://bmjopen.bmj.com/site/about/guidelines.xhtml

For peer review only

drug refill, and other aspects of patient management for people with hypertension and diabetes. The present study highlighted the potential to involve peripheral primary care facilities and community health workers to rapidly expand much needed hypertension and diabetes services in Bangladesh, and similar global settings. Further investigations are needed to evaluate the effectiveness of decentralized hypertension and diabetes care.

For peer review only

For peer review only - http://bmjopen.bmj.com/site/about/guidelines.xhtml

Contributors

MM, JC, IG, BO, RP, and FH contributed to the conception and design of the study. IG, AR, FH, MM, RP, MH, and AA worked on the development of the digital platform. RRP, AA, MM, and MH contributed to implementation of data collection and management. WX implemented analysis of data and wrote the first draft of the manuscript. All authors contributed to interpretation of data, and revision of the manuscript.

Competing interests

None.

Funding

This research was funded by the National Institute for Health Research (NIHR) (16/136/68). The funder had no role in study design, data collection and analysis, decision to publish, or preparation of the manuscript.

Data availability statement

The South Asia Biobank data are available to researchers upon request to the study Data Access Committee. Contact forms and emails are provided on the GHRU website (www.ghru-southasia.org).

Ethics approval

This study was approved by the Institutional Review Board of BRAC University (IRB reference number 2020-009-IR).

For peer review only

For peer review only - http://bmjopen.bmj.com/site/about/guidelines.xhtml

References

[1] H. Sun *et al.*, "IDF Diabetes Atlas: Global, regional and country-level diabetes prevalence estimates for 2021 and projections for 2045," *Diabetes research and clinical practice,* vol. 183, p. 109119, 2022.

[2] G. A. Roth *et al.*, "Global Burden of Cardiovascular Diseases and Risk Factors, 1990–2019: Update From the GBD 2019 Study," *Journal of the American College of Cardiology,* vol. 76, no. 25, pp. 2982-3021, 2020/12/22/ 2020, doi: https://doi.org/10.1016/j.jacc.2020.11.010.

[3] D. Prabhakaran *et al.*, "Cardiovascular, respiratory, and related disorders: key messages from Disease Control Priorities," *The Lancet,* vol. 391, no. 10126, pp. 1224-1236, 2018.

[4] B. Zhou *et al.*, "Worldwide trends in hypertension prevalence and progress in treatment and control from 1990 to 2019: a pooled analysis of 1201 population-representative studies with 104 million participants," *The Lancet,* vol. 398, no. 10304, pp. 957-980, 2021.

[5] W. H. Organization, "Global action plan for the prevention and control of NCDs 2013–2020. 2013," *Geneva: WHO,* 2019.

[6] J. Manne-Goehler *et al.*, "Health system performance for people with diabetes in 28 low-and middle-income countries: a cross-sectional study of nationally representative surveys," *PLoS medicine,* vol. 16, no. 3, p. e1002751, 2019.

[7] P. Geldsetzer *et al.*, "The state of hypertension care in 44 low-income and middle-income countries: a cross-sectional study of nationally representative individual-level data from 1· 1 million adults," *The Lancet,* vol. 394, no. 10199, pp. 652-662, 2019.

[8] A. Kabir, M. N. Karim, R. M. Islam, L. Romero, and B. Billah, "Health system readiness for non-communicable diseases at the primary care level: a systematic review," *BMJ open,* vol. 12, no. 2, p. e060387, 2022.

[9] J. Frenk *et al.*, "Health professionals for a new century: transforming education to strengthen health systems in an interdependent world," *The lancet,* vol. 376, no. 9756, pp. 1923-1958, 2010.

[10] H. A. Chowdhury *et al.*, "Assessing service availability and readiness of healthcare facilities to manage diabetes mellitus in Bangladesh: Findings from a nationwide survey," *PloS one,* vol. 17, no. 2, p. e0263259, 2022.

[11] K. Islam, R. Huque, K. Saif-Ur-Rahman, E. K. ANM, and E. H. AHM, "Implementation status of non-communicable disease control program at primary health care level in Bangladesh: Findings from a qualitative research," *Public Health in Practice,* p. 100271, 2022.

[12] W. H. Organization, "Declaration of alma-ata," World Health Organization. Regional Office for Europe, 1978.

[13] L. B. Rawal *et al.*, "Non-communicable disease (NCD) corners in public sector health facilities in Bangladesh: a qualitative study assessing challenges and opportunities for improving NCD services at the primary healthcare level," *BMJ Open,* vol. 9, no. 10, p. e029562, 2019, doi: 10.1136/bmjopen-2019-029562.

[14] L. Rawal, S. Jubayer, S. R. Choudhury, S. M. S. Islam, and A. S. Abdullah, "Community health workers for non-communicable diseases prevention and

For peer review only - http://bmjopen.bmj.com/site/about/guidelines.xhtml

control in Bangladesh: a qualitative study," *Global Health Research and Policy,* vol. 6, no. 1, p. 1, 2021/01/06 2021, doi: 10.1186/s41256-020-00182-z.

[15] R. Devi, K. Kanitkar, R. Narendhar, K. Sehmi, and K. Subramaniam, "A Narrative Review of the Patient Journey Through the Lens of Non-communicable Diseases in Low- and Middle-Income Countries," *Advances in Therapy,* vol. 37, no. 12, pp. 4808-4830, 2020/12/01 2020, doi: 10.1007/s12325-020-01519-3.

[16] D. Jindal *et al.*, "Improving care for hypertension and diabetes in india by addition of clinical decision support system and task shifting in the national NCD program: I-TREC model of care," *BMC Health Services Research,* vol. 22, no. 1, pp. 1-12, 2022.

[17] A. T. Brennan *et al.*, "Outcomes of stable HIV-positive patients down-referred from a doctor-managed antiretroviral therapy clinic to a nurse-managed primary health clinic for monitoring and treatment," *Aids,* vol. 25, no. 16, pp. 2027-2036, 2011. [Online]. Available: https://www.ncbi.nlm.nih.gov/pmc/articles/PMC3669640/pdf/nihms-446164.pdf.

[18] I. Sanne *et al.*, "Nurse versus doctor management of HIV-infected patients receiving antiretroviral therapy (CIPRA-SA): a randomised non-inferiority trial," *The Lancet,* vol. 376, no. 9734, pp. 33-40, 2010.

[19] U. Lehmann, W. Van Damme, F. Barten, and D. Sanders, "Task shifting: the answer to the human resources crisis in Africa?," *Human resources for health,* vol. 7, no. 1, pp. 1-4, 2009.

[20] M. E. Kruk, G. Nigenda, and F. M. Knaul, "Redesigning primary care to tackle the global epidemic of noncommunicable disease," *American journal of public health,* vol. 105, no. 3, pp. 431-437, 2015.

[21] T. Kredo, N. Ford, F. B. Adeniyi, and P. Garner, "Decentralising HIV treatment in lower ‐ and middle ‐ income countries," *Cochrane database of systematic reviews,* no. 6, 2013.

[22] A. B. Labrique *et al.*, "Best practices in scaling digital health in low and middle income countries," *Globalization and health,* vol. 14, no. 1, pp. 1-8, 2018.

[23] R. Anchala, S. Kaptoge, H. Pant, E. Di Angelantonio, O. H. Franco, and D. Prabhakaran, "Evaluation of effectiveness and cost ‐ effectiveness of a clinical decision support system in managing hypertension in resource constrained primary health care settings: results from a cluster randomized trial," *Journal of the American Heart Association,* vol. 4, no. 1, p. e001213, 2015. [Online]. Available: https://www.ahajournals.org/doi/pdf/10.1161/JAHA.114.001213?download=true.

[24] D. Prabhakaran *et al.*, "Effectiveness of an mHealth-Based Electronic Decision Support System for Integrated Management of Chronic Conditions in Primary Care," *Circulation,* vol. 139, no. 3, pp. 380-391, 2019, doi: doi:10.1161/CIRCULATIONAHA.118.038192.

[25] A. Kumar *et al.*, "Designing and implementing an integrated non-communicable disease primary care intervention in rural Nepal," *BMJ global health,* vol. 4, no. 2, p. e001343, 2019. [Online]. Available: https://escholarship.org/content/qt42v9w4rk/qt42v9w4rk.pdf?t=qf72ye.

[26] T. Vos *et al.*, "Global burden of 369 diseases and injuries in 204 countries and territories, 1990–2019: a systematic analysis for the Global Burden of Disease Study 2019," *The Lancet,* vol. 396, no. 10258, pp. 1204-1222, 2020.

For peer review only - http://bmjopen.bmj.com/site/about/guidelines.xhtml

[27] N. J. Sathi, M. A. Islam, M. S. Ahmed, and S. M. S. Islam, "Prevalence, trends and associated factors of hypertension and diabetes mellitus in Bangladesh: Evidence from BHDS 2011 and 2017–18," *PloS one,* vol. 17, no. 5, p. e0267243, 2022. [Online]. Available: https://www.ncbi.nlm.nih.gov/pmc/articles/PMC9064112/pdf/pone.0267243.pdf.
[28] N. Khan, J. C. Oldroyd, M. B. Hossain, and R. M. Islam, "Awareness, treatment, and control of diabetes in Bangladesh: evidence from the Bangladesh Demographic and Health Survey 2017/18," *International Journal of Clinical Practice,* vol. 2022, 2021.
[29] M. N. Khan *et al.*, "Prevalence, awareness, treatment, and control of hypertension in Bangladesh: Findings from National Demographic and Health Survey, 2017–2018," *The Journal of Clinical Hypertension,* vol. 23, no. 10, pp. 1830-1842, 2021. [Online]. Available: https://www.ncbi.nlm.nih.gov/pmc/articles/PMC8678656/pdf/JCH-23-1830.pdf.
[30] M. N. Khan, J. C. Oldroyd, M. B. Hossain, and R. M. Islam, "Awareness, treatment, and control of diabetes in Bangladesh: evidence from the Bangladesh Demographic and Health Survey 2017/18," *medRxiv,* 2021.
[31] B. K. Riaz *et al.*, "Risk factors for non-communicable diseases in Bangladesh: findings of the population-based cross-sectional national survey 2018," *BMJ open,* vol. 10, no. 11, p. e041334, 2020. [Online]. Available: https://www.ncbi.nlm.nih.gov/pmc/articles/PMC7703434/pdf/bmjopen-2020-041334.pdf.
[32] D. G. o. H. S. Non-communicable Disease Control Programme, "Multi-sectoral action plan for prevention and control of non-communicable diseases 2018-2025," ed, 2018.
[33] W. Bank, *Hypertension and Type-2 Diabetes in Bangladesh: Continuum of Care Assessment and Opportunities for Action*. World Bank, 2018.
[34] M. o. H. a. F. W. Government of People's Republic of Bangladesh, "Facility Registry," ed, 2022.
[35] W. H. Organization, "WHO package of essential noncommunicable (PEN) disease interventions for primary health care," 2020.
[36] G. Zou and A. Donner, "Extension of the modified Poisson regression model to prospective studies with correlated binary data," *Statistical methods in medical research,* vol. 22, no. 6, pp. 661-670, 2013.
[37] L. N. Yelland, A. B. Salter, and P. Ryan, "Performance of the modified Poisson regression approach for estimating relative risks from clustered prospective data," *American journal of epidemiology,* vol. 174, no. 8, pp. 984-992, 2011.
[38] BRAC, "Accessible and affordable primary health care for all: annual report progress of social enterprises April 2018 - April 2019," BRAC Health Nutrition and Population Program, 2020. [Online]. Available: http://www.brac.net/program/wp-content/uploads/2019/09/HNPP-SE-Annual-Report-18-19.pdf
[39] B. B. Alam, A. Koziel, N. Fraser-Hurt, Z. Shubber, A. F. Olabiran, and J. B. Mamza, "Hypertension and Type-2 diabetes in Bangladesh: continuum of care assessment and opportunities for action," 2020.
[40] N. S. Venkateshmurthy *et al.*, "m-Power Heart Project-a nurse care coordinator led, mHealth enabled intervention to improve the management of hypertension in

For peer review only - http://bmjopen.bmj.com/site/about/guidelines.xhtml

India: study protocol for a cluster randomized trial," *Trials,* vol. 19, no. 1, pp. 1-9, 2018.

[41] D. Peiris *et al.*, "SMARThealth India: a stepped-wedge, cluster randomised controlled trial of a community health worker managed mobile health intervention for people assessed at high cardiovascular disease risk in rural India," *PLoS One,* vol. 14, no. 3, p. e0213708, 2019. [Online]. Available: https://www.ncbi.nlm.nih.gov/pmc/articles/PMC6435227/pdf/pone.0213708.pdf.

[42] N. D. Labhardt, J.-R. Balo, M. Ndam, J.-J. Grimm, and E. Manga, "Task shifting to non-physician clinicians for integrated management of hypertension and diabetes in rural Cameroon: a programme assessment at two years," *BMC health services research,* vol. 10, no. 1, pp. 1-10, 2010.

[43] C. Kachimanga *et al.*, "Implementation of a non-communicable disease clinic in rural Sierra Leone: early experiences and lessons learned," *Journal of public health policy,* vol. 42, no. 3, pp. 422-438, 2021. [Online]. Available: https://www.ncbi.nlm.nih.gov/pmc/articles/PMC8452567/pdf/41271_2021_Article_304.pdf.

[44] S. Deo and P. Singh, "Community health worker-led, technology-enabled private sector intervention for diabetes and hypertension management among urban poor: a retrospective cohort study from large Indian metropolitan city," *BMJ Open,* vol. 11, no. 8, p. e045246, 2021, doi: 10.1136/bmjopen-2020-045246.

[45] J. He *et al.*, "Effect of a Community Health Worker–Led Multicomponent Intervention on Blood Pressure Control in Low-Income Patients in Argentina: A Randomized Clinical Trial," *JAMA,* vol. 318, no. 11, pp. 1016-1025, 2017, doi: 10.1001/jama.2017.11358.

[46] T. H. Jafar *et al.*, "A community-based intervention for managing hypertension in rural South Asia," *New England Journal of Medicine,* vol. 382, no. 8, pp. 717-726, 2020. [Online]. Available: https://www.nejm.org/doi/pdf/10.1056/NEJMoa1911965?articleTools=true.

[47] J.-D. Schwalm *et al.*, "A community-based comprehensive intervention to reduce cardiovascular risk in hypertension (HOPE 4): a cluster-randomised controlled trial," *The Lancet,* vol. 394, no. 10205, pp. 1231-1242, 2019/10/05/ 2019, doi: https://doi.org/10.1016/S0140-6736(19)31949-X.

[48] C. K. Chow *et al.*, "Prevalence, awareness, treatment, and control of hypertension in rural and urban communities in high-, middle-, and low-income countries," *Jama,* vol. 310, no. 9, pp. 959-968, 2013.

For peer review only

For peer review only - http://bmjopen.bmj.com/site/about/guidelines.xhtml

Figure 1. NCD service delivery hierarchy and coordinated NCD care

GHRU global health research unit; CHCP community health care provider; CHW community health worker; CC community clinic; UHC Upazila Health Complex; NCD non-communicable disease

* Government community health workers include family welfare assistants, health assistants, and multipurpose health volunteers; however, a designated community health worker was recruited and trained for NCD related tasks in the present study. Union subcentres were not involved in this present study. Union subcentres are at higher level of the hierarchy than community clinics.

Figure 2. digital platform designed to support multiple healthcare personnel roles along the care pathway

Figure 3. Change in proportion of uncontrolled conditions over follow-up visits *

* BP blood pressure
Results adjusted age and sex
Uncontrolled blood pressure is defined as systolic blood pressure ≥140 mmHg or diastolic blood pressure ≥90 mmHg, while uncontrolled blood glucose is defined as fasting blood glucose ≥7 mmol/L or random blood glucose ≥11.1 mmol/L.

For peer review only

Table 1. Patient characteristics at enrolment

Characteristics *	Entire Sample (n=624)	Hypertension only (n=404)	Diabetes only (n=75)	Hypertension & Diabetes (n=145)
Age in years, mean (sd)	59.5 (12.0)	61.5 (12.3)	55.1 (10.7)	56.4 (10.6)
Age				
40-49	136 (21.8)	68 (16.8)	28 (37.3)	40 (27.6)
50-59	179 (28.7)	110 (27.2)	20 (26.7)	49 (33.8)
60-69	169 (27.1)	116 (28.7)	18 (24.0)	35 (24.1)
70-100	140 (22.4)	110 (27.2)	9 (12.0)	21 (14.5)
Sex				
Female	406 (65.1)	274 (67.8)	41 (54.7)	91 (62.4)
Male	218 (34.9)	130 (32.2)	34 (45.3)	54 (37.6)
Religion				
Hindu	78 (12.5)	57 (14.1)	4 (5.3)	17 (11.7)
Islam	546 (87.5)	347 (85.9)	71 (94.7)	128 (88.3)
Education				
No formal school	281 (45.0)	199 (49.3)	24 (32.0)	58 (40.0)
Primary or lower	172 (27.6)	118 (29.2)	22 (29.3)	32 (22.1)
Secondary or higher	171 (27.4)	87 (21.5)	29 (38.7)	55 (37.9)
Community clinic				
Khorakhai	204 (32.7)	120 (29.7)	33 (44.0)	51 (35.2)
Jahanabad	202 (32.4)	132 (32.7)	23 (30.7)	47 (32.4)
Manmathapur	218 (34.9)	152 (37.6)	19 (25.3)	47 (32.4)
Tabaco use				
No	322 (51.6)	203 (50.3)	38 (50.7)	81 (55.9)
Yes	302 (48.4)	201 (49.7)	37 (49.3)	64 (44.1)
BMI categories				
<18.5 kg/m^2	68 (10.9)	55 (13.6)	6 (8.0)	7 (4.8)
18.5-22.9 kg/m^2	243 (38.9)	159 (39.4)	26 (34.7)	58 (40.0)
≥23.0 kg/m^2	313 (50.2)	190 (47.0)	43 (57.3)	80 (55.2)
Hypertension				
Baseline BP controlled	281 (39.7)	145 (35.9)	n/a	73 (50.3)
Baseline BP uncontrolled †	331 (60.3)	259 (64.1)	n/a	72 (49.7)
Diabetes				
Baseline glucose controlled	57 (25.9)	n/a	15 (20.0)	42 (29.0)
Baseline glucose uncontrolled †	163 (74.1)	n/a	60 (80.0)	103 (71.0)

BP, blood pressure; n/a, not applicable

* n (%) are presented, unless indicated otherwise.

† Uncontrolled blood pressure is defined as systolic blood pressure ≥140 mmHg or diastolic blood pressure ≥90 mmHg, while uncontrolled blood glucose is defined as fasting blood glucose ≥7 mmol/L or random blood glucose ≥11.1 mmol/L.

For peer review only - http://bmjopen.bmj.com/site/about/guidelines.xhtml

Table 2. Change in proportion of uncontrolled conditions over follow-up visits

Visit	Blood pressure		Blood glucose	
	Entire sample	Baseline uncontrolled cases †	Entire sample	Baseline uncontrolled cases ‡
1 month	0.63 (0.55, 0.72)	0.53 (0.47, 0.61)	0.69 (0.58, 0.82)	0.57 (0.48, 0.67)
2 months	0.48 (0.39, 0.60)	0.38 (0.30, 0.47)	0.66 (0.54, 0.81)	0.51 (0.41, 0.63)
3 months	0.44 (0.33, 0.57)	0.35 (0.26, 0.47)	0.60 (0.45, 0.78)	0.49 (0.38, 0.64)
4 months	0.49 (0.35, 0.69)	0.38 (0.27, 0.53)	0.54 (0.36, 0.80)	0.41 (0.26, 0.62)
5 months	0.64 (0.32, 1.25)	0.37 (0.18, 0.76)	0.44 (0.20, 0.96)	0.16 (0.05, 0.54)
Constant	0.44 (0.30, 0.64)	0.99 (0.93, 1.05)	1.04 (0.67, 1.60)	1.33 (0.98, 1.78)
N	549	331	220	163

* Models adjusted for age (40-49, 50-59, 60-69, 70-100), sex (male, female), education (no formal education, primary or lower, secondary or higher), religion (Islam, Hindu), BMI category (under, normal, overweight or obese), community clinics (Khorakhai, Manmathapur, Jahanabad).

† Baseline uncontrolled cases were defined patients with systolic blood pressure ≥ 140 mmHg or diastolic blood pressure ≥ 90 mmHg

‡ Baseline uncontrolled cases were defined patients with fasting plasma glucose ≥ 7.0 mmol/L or random plasma glucose ≥ 11.1 mmol/L

For peer review only - http://bmjopen.bmj.com/site/about/guidelines.xhtml

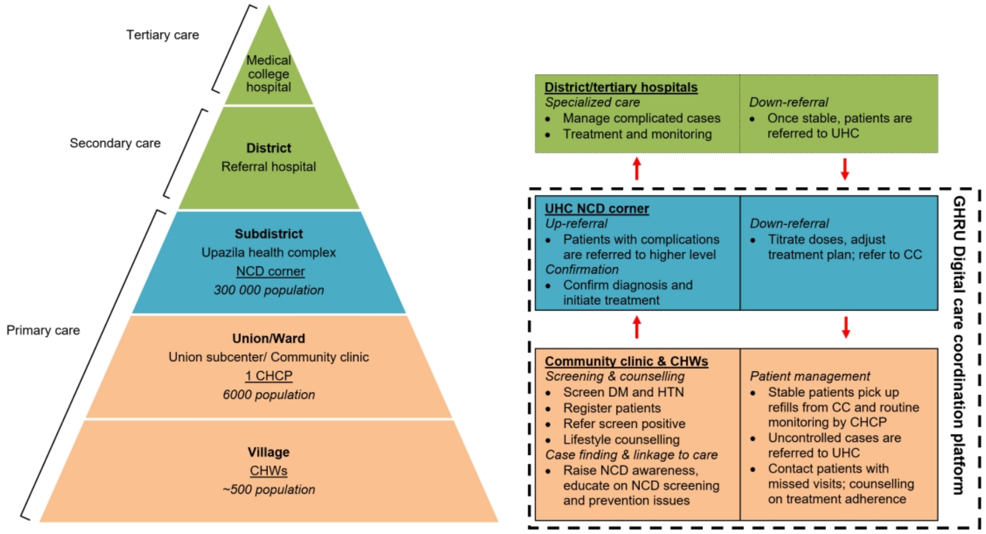

Figure 1. GHRU global health research unit; CHCP community health care provider; CHW community health worker; CC community clinic; UHC Upazila Health Complex; NCD non-communicable disease
* Government community health workers include family welfare assistants, health assistants, and multipurpose health volunteers; however, a designated CHW was recruited and trained for NCD related tasks in the present study. Union subcentres were not involved in this present study. Union subcentres are at higher level of the hierarchy than community clinics.

487x297mm (144 x 144 DPI)

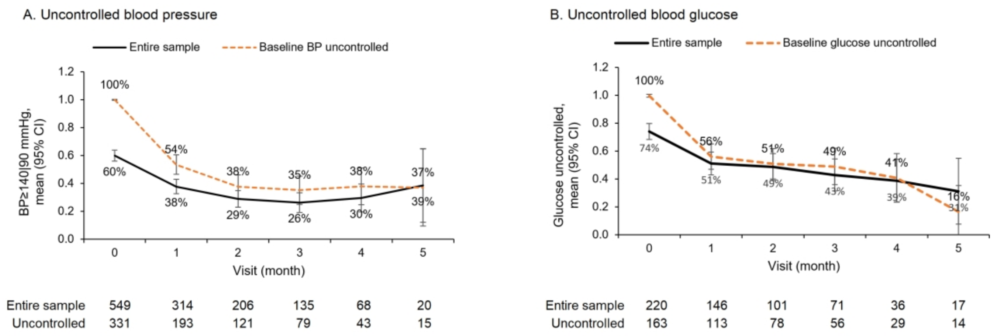

Figure 2. Change in proportion of uncontrolled conditions over follow-up visits

BP blood pressure Results adjusted age and sex Uncontrolled blood pressure is defined as systolic blood pressure ≥140 mmHg or diastolic blood pressure ≥90 mmHg, while uncontrolled blood glucose is defined as fasting blood glucose ≥7 mmol/L or random blood glucose ≥11.1 mmol/L.

563x190mm (144 x 144 DPI)

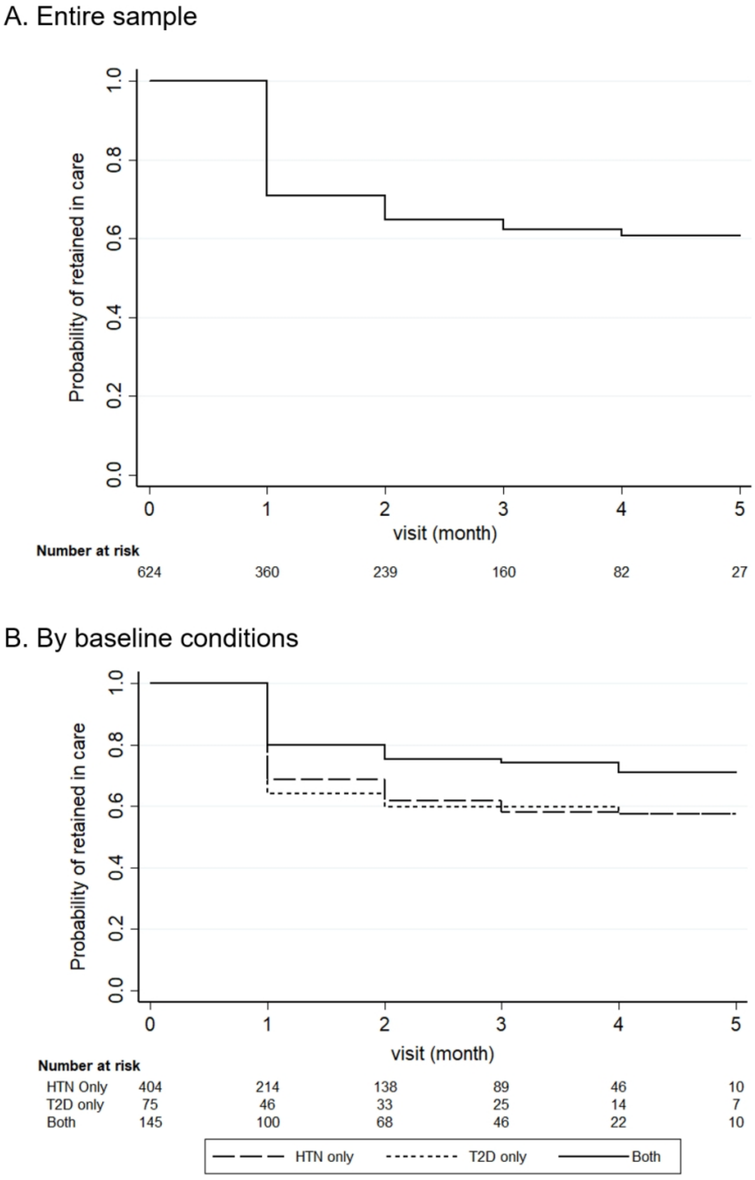

Figure 3. Patient loss to follow-up by baseline diagnosis

HTN hypertension; T2D type 2 diabetes * Loss to follow-up is defined as no consultation recorded for two months.Survival function estimated by the Kaplan-Meier method.Time interval between two adjacent visits is approximately one month.

203x288mm (144 x 144 DPI)

Supplementary Materials

TITLE: Enhancing Care Quality and Accessibility through Digital Technology-Supported Decentralization of Hypertension and Diabetes Management: A Proof-of-Concept Study in Rural Bangladesh

For peer review only

For peer review only - http://bmjopen.bmj.com/site/about/guidelines.xhtml

The development and deployment of a digital platform for primary care hypertension and diabetes care coordination in Bangladesh

Background

The long-term nature of hypertension and diabetes (and other noncommunicable diseases), which require regular engagement and health monitoring for improved patient outcomes, make it critical for healthcare personnel in primary health care (PHC) teams to be collaborative and coordinated to ensure efficient and effective care delivery.[1,2] Digital platforms have the potential to enable greater collaboration and coordination that will be helpful in addressing many of the barriers to effective NCD management within the PHC system.[3] Digital platforms can help the health care system to document the risks and health states of the population, and to prioritize delivery of care to those at greatest need. Through more efficient data capture and sharing, guideline-based decision support, generation of individualized care plans and management of care delivery actions, the implementation of digital platforms support the move towards coordinated, evidence-based and data-driven NCD management for populations served by the PHC team.[4]

Identification of gaps in usual care

The first step in identifying the components of the intervention was to assess the gaps in usual care through systematic reviews of literature on epidemiological studies, NCD service delivery in LMIC and in Bangladesh, and through discussion with experts and stake holders. Multiple meetings with primary care physicians, NCD nurses, community health workers, and local health authorities were organized to understand the current care pathway, and to identify gaps in usual care. Consultations with domain experts, including cardiologists, nutritionists, epidemiologists, statisticians, data scientists, pharmacists, were conducted. The team mapped out the journey of patients under current model of government service delivery and a model of decentralized care with the involvement of the most peripheral primary care facilities and community-based interventions by community health workers (CHWs).

Through these processes, a few key gaps were identified include 1) using of paper-based health records limits exchange of critical information, 2) adherence to national guidelines and treatment protocols is inconsistent among health care providers, 3) patient management of medication adherence and follow-up are irregular, 4) lack of coordination in service delivery which involves a wide range of settings and personnel, which included subdistrict level primary care facilities, community clinics - the most peripheral primary health care facilities, and doctors, nurses, community healthcare providers (CHCPs), and CHWs.

For peer review only - http://bmjopen.bmj.com/site/about/guidelines.xhtml

Overview of the development processes

The digital platform was developed using the user-centred design (UCD), an iterative software development methodology in which the development team focuses on the users and their needs in each phase of the development process.[5] A multi-disciplinary technology development team consisting of domain experts, software engineers and user design researchers utilized UCD in the development of the Coordinate platform that with each iteration, produced a usable version of the software that was increasingly aligned with the needs of its users. The process was completed when a sufficiently developed version of the software was produced that could be deployed for use in the study.

One of the core value propositions of Coordinate platform is to use it in the community. In rural areas of Bangladesh, internet connectivity is a major problem, and many online platforms need to adopt offline functionality to operate in rural areas. Hence, offline functionality of the Coordinate platform was designed with different use cases and functionalities.

The Android OS was chosen as the operating system platform due to the greater flexibility it offers in app development, testing and deployment, along with users' familiarity due to widespread usage in South Asia and the availability of low-cost tablets. The platform is underpinned by an administrator web-dashboard application built using Laravel, an open-source PHP framework and connected to a database built using PostgresSQL, open-source relational database management system. The platform was development through four iterative loops from June 2020 to December 2021. The platform was designed following international standards of interoperability, with clearly documented application programming interfaces. The digital platform resources are open-source and can be readily utilized by the global health community.

Content and functionalities

Based on the results from the need assessment, the digital platform was built to support the needs of various health service providers, including new users (CHCPs, CHWs) who would be trained to provided care in a decentralized care model. Accordingly, a core set of functionalities was developed to support the coordination of patient care through enhanced data capture and sharing, guideline-based decision support, generation of individualized care plans and management of care delivery actions (**Supplementary Figure B**).

The system consists of a tablet-based application for community health workers to register participants at risk, administer in-field questionnaire, deliver care plan, and refer. CHCPs at community clinics have access to information entered by CHWs. CHCPs will then perform a simplified structured clinical evaluation, including medical

For peer review only - http://bmjopen.bmj.com/site/about/guidelines.xhtml

history, anthropometrics, blood pressure, and blood glucose. Based on the information obtained, the CHCPs conduct lifestyle education for high-risk behaviours with assistance from built-in point-of-care support and referred the participant with elevated risks for further confirmation at subdistrict NCD clinic. The participant's electronic health record will be updated to reflect the additional information obtained by CHCPs and be available to nurses and doctors at NCD clinic.

Once a participant approaches NCD clinic, nurses perform additional examinations and update patient data. The platform generates CVD risk scores based on WHO cardiovascular disease risk non-laboratory-based charts (South Asia) and have all pertinent data for physicians to make treatment decision and management plan, which are then incorporated into patient profile. The patient list is then generated and continuously updated for CHWs in the community and CHCPs at community clinics to follow up with routine examination, medication refill, and counselling. Some of the user interfaces were illustrated in **Supplementary Figure C**.

The doctors and nurses have access to a web-based central administration dashboard which allow them to track patient treatment progress and referrals, and to add/remove users (**Supplementary Figure D**). A demo video overview of the platform is available at https://youtu.be/nXmlX0_RZVY.

Conclusions

The digital platform is among the first to support a coordinated primary care delivery for hypertension and diabetes management across all levels of primary care providers and community health workers. Digital tools can be invaluable in strengthening health systems, although they need to be an integral part of an intervention package to exert effects within specific health system contexts.[6] Multifaceted intervention is often needed to maximize the impact of a digital tool.[2] Following international standards of interoperability throughout the designing phrase, the digital platform resources are open-source and can be readily utilized by the global health community.

In the next steps, we are planning to add training and education of primary care providers and patient self-management components to the platform. Specifically, we planned to develop online training program in collaboration with domain experts and key stakeholders. Digital training modules will be developed for use by PHC teams, tailored to user role and setting. These highly scalable modules for development of NCD care skills may reduce the need for in-person sessions. In addition, to empower community users, we planned to develop a user-facing module that gives participants access to their health information, personalised care plans, and evidence-based self-management content. Finally, we will refine and extend on the open-source decision-support algorithms in our platform to simplify the process of tailoring to local context, and to

For peer review only - http://bmjopen.bmj.com/site/about/guidelines.xhtml

enhance formulation of adaptive longitudinal care plans. This includes taking into account additional parameters in decision-making to strengthen care plan recommendations and drive effective disease management.

References

1. Kabir, A., Karim, M. N., Islam, R. M., Romero, L. & Billah, B. Health system readiness for non-communicable diseases at the primary care level: a systematic review. BMJ open 12, e060387 (2022).
2. Kruk, M. E., Nigenda, G. & Knaul, F. M. Redesigning primary care to tackle the global epidemic of noncommunicable disease. American journal of public health 105, 431-437 (2015).
3. Labrique, A. B. et al. Best practices in scaling digital health in low and middle income countries. Globalization and health 14, 1-8 (2018).
4. Mechael, P. et al. Barriers and gaps affecting mHealth in low and middle income countries: Policy white paper. (2010).
5. Bazzano AN, Martin J, Hicks E, Faughnan M, Murphy L. Human-centred design in global health: A scoping review of applications and contexts. Virgili G, editor. PLOS ONE 2017 Nov 1;12(11):e0186744.
6. Praveen, D., et al. (2014). "SMARTHealth India: development and field evaluation of a mobile clinical decision support system for cardiovascular diseases in rural India." JMIR mHealth and uHealth **2**(4): e3568.

For peer review only - http://bmjopen.bmj.com/site/about/guidelines.xhtml

Supplementary Figure A. Bangladesh national protocol for integrated management of hypertension, diabetes and cholesterol using a total cardiovascular risk approach

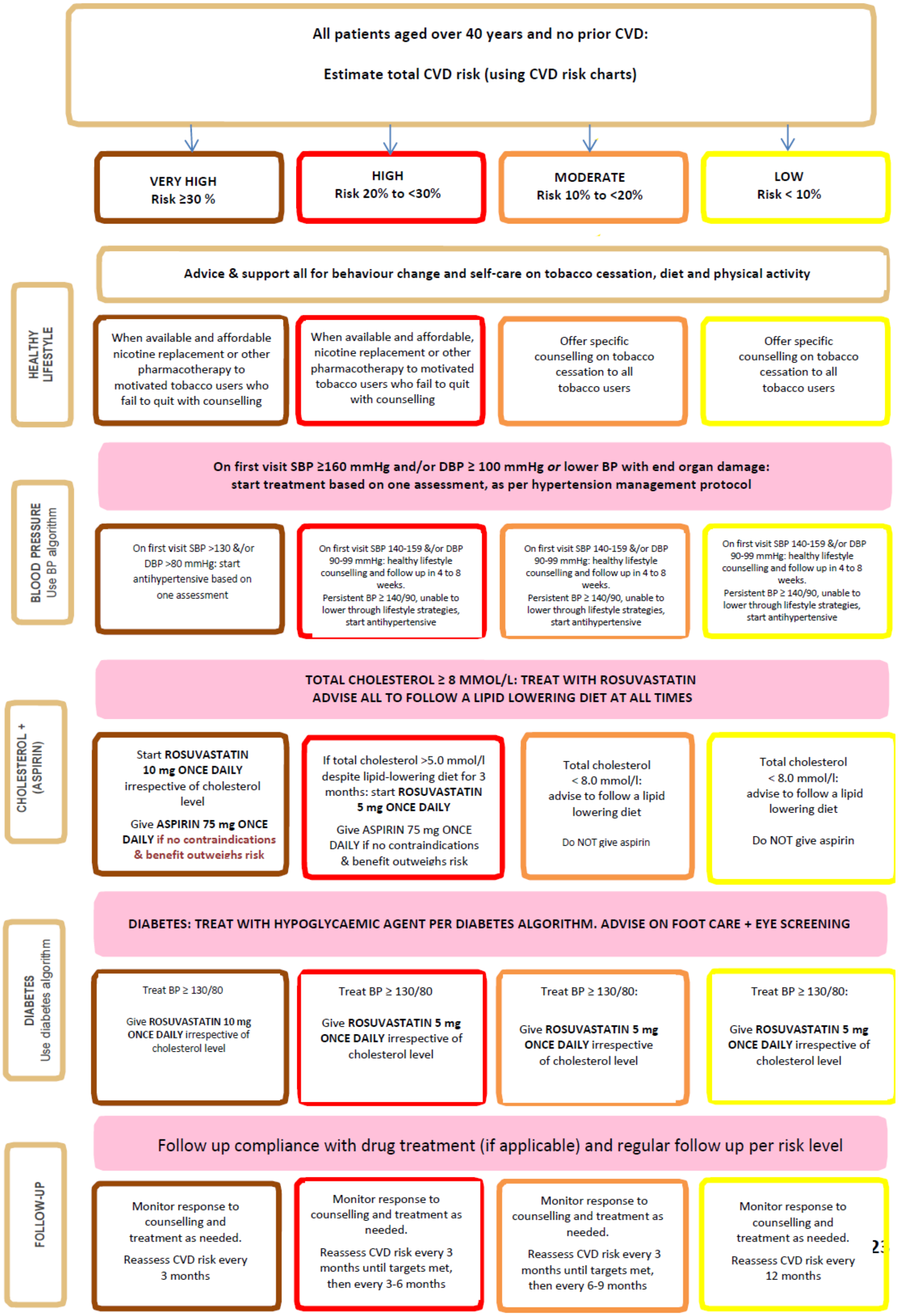

For peer review only - http://bmjopen.bmj.com/site/about/guidelines.xhtml

Bangladesh national protocol for management of hypertension at primary health care settings

Decision to start antihypertensive medication taken based on estimation of total cardiovascular disease (CVD) risk of patients aged ≥ 40 years [a, b]

Step 1: **START AMLODIPINE 5 mg ONCE DAILY** [c]

Step 2: Review after 1 month, if treatment target not met [d,e]
ADD LOSARTAN 50 mg ONCE DAILY

Step 3: Review after 1 month, if treatment target not met [d]
ADD HYDROCHLOROTHIAZIDE 12.5 mg ONCE DAILY

Step 4: Review after 1 month, if treatment target not met [d]
REFER TO A SPECIALIST

THIS PROTOCOL SHOULD NOT BE USED FOR PATIENTS WHO *ARE* PREGNANT

IF PREGNANT, REFER TO SPECIALIST [f]

DO NOT PRESCRIBE LOSARTAN (OR ANY ACE-I OR ARBs) to women who *may* become pregnant.

For women of childbearing age who *may* become pregnant:

- Start amlodipine 2.5 – 5 mg once daily
- Review after one month. If treatment target not met, increase amlodipine to 10 mg once daily [d]
- Review after one month, if treatment target not met, REFER [d]

a. Refer to 'Protocol for Integrated Management of Hypertension, Diabetes and Cholesterol using **'Total Cardiovascular Risk Approach'** for **making decision** to treat hypertension in patients **aged ≥ 40 years.**
b. This protocol may also be used to treat patients below 40 years of age when treatment for hypertension is indicated.
c. Consider Amlodipine 2.5 mg as starting dose for patients aged ≥ 60 years and/ or patients with low body weight (BMI ≤ 18.5).
d. Check that patient has been taking drugs regularly and correctly, prior to increasing dose.
e. Before initiating and several weeks after starting losartan, check serum creatinine and serum potassium. **Avoid losartan for women of childbearing age who may become pregnant.**
f. If treatment for hypertension is to be initiated for pregnant women before referring her to specialist, consider methyldopa 125 – 250 mg twice daily

Treatment target based on CVD risk based management	
Most patients	≤ 140/90 mmHg
Having comorbidity: diabetes, ischaemic heart disease, stroke, chronic kidney disease	≤ 130/80 mmHg
High CVD Risk >30% (as per CVD risk prediction chart)	≤ 130/80 mmHg

LILFESTYLE MODIFICATION ADVICE FOR ALL PATIENTS

- Stop all tobacco use, avoid secondhand tobacco smoke.
- Stop taking alcohol.
- Increase physical activity to equivalent of brisk walk 150 minutes per week.
- If overweight, lose weight.
- Eat heart-healthy diet:
  - Reduce dietary salt intake.
  - Eat ⩾ 5 servings of vegetables/fruit per day.
  - Use healthy oils, such as sesame (Til), olive, safflower, sunflower.
  - Eat nuts, peas, whole grains and foods rich in potassium like spinach, watermelon, yogurt and banana.
  - Limit red meat to once or twice a week at most.
  - Eat fish or other food rich in omega 3 fatty acids at least twice a week.
  - Avoid added sugar from sweets, cakes, cookies, fizzy drinks, sugar sweetened beverages

Bangladesh National Protocol for Management of Type 2 Diabetes at Primary Health Care Level

Test patients who have symptoms of diabetes. Test adults who are 40+ years old. Test pregnant women during antenatal checkup

Diabetes diagnosed as per diagnostic criteria

Advise on diet, physical exercise and healthy lifestyle

Goal for glycaemic control: Fasting plasma glucose 4.4 – 7.2 mmol/L; Postprandial plasma glucose ≤ 10 mmol/L; Glycated haemoglobin ≤ 7%

Fasting Plasma Glucose (FPG) 7.0 – 10.0 mmol/L, or<br>Random Plasma Glucose (RPG) 11.1 – 12.0 mmol/L, or<br>Glycated Haemoglobin (A1C) 6.5 - 8.5%	FPG 10.1 – 14 mmol/L, or<br>RPG 12.1 – 18 mmol/L, or<br>A1C 8.6 – 10.0%	FPG > 14 mmol/L, or<br>RPG > 18 mmol/L, or<br>A1C > 10.0%
Counsel on diet and physical activity<br>↓<br>Review in 1 month.<br>If glycaemic goal not achieved:<br>Initiate with METFORMIN 250 – 500 mg once daily. *<br>Counsel on diet, physical activity and adherence to treatment at all visits.<br>↓<br>Review in 1 month.<br>If goal not achieved, increase dose of METFORMIN to 500 mg twice daily<br>↓<br>Review in 1 month.<br>If goal not achieved, increase dose of METFORMIN to 1000 mg twice daily<br>↓<br>Review in 3 month.<br>If goal not achieved, *ADD* GLICLAZIDE, 40 mg once a day<br>Counsel on hypoglycemia at all subsequent visits<br>↓<br>Review in one month.<br>If goal not achieved, increase dose of GLICLAZIDE by 40 mg every month till goal is achieved/ maximum dose of 80 mg twice daily is reached<br>↓	Start METFORMIN 500 mg twice daily +<br>GLICLAZIDE 40 mg twice daily. *<br>Counsel on diet, physical activity and adherence to treatment at all visits<br>↓<br>Review in 1 month.<br>If glycaemic goal not achieved, increase dose of METFORMIN to 1000 mg twice daily<br>↓<br>Review in 1 month.<br>If goal not achieved, increase dose of GLICLAZIDE by 40 mg every month, till 80 mg twice daily<br>↓	REFER to higher-level of care for starting insulin
Review in 3 months.<br>If goal not achieved, despite adherence to medication, healthy diet, and physical activity, REFER to higher-level of care for starting insulin		

* If serum creatinine is > 1.5 mg/ dl or there is contraindication/ intolerance to Metformin, start with Gliclazide 40 mg daily. Review and increase dose by 40 mg every month till goal is achieved/ maximum recommended dose is reached.

For peer review only - http://bmjopen.bmj.com/site/about/guidelines.xhtml

Supplementary Figure B. digital platform designed to support multiple healthcare personnel roles along the care pathway

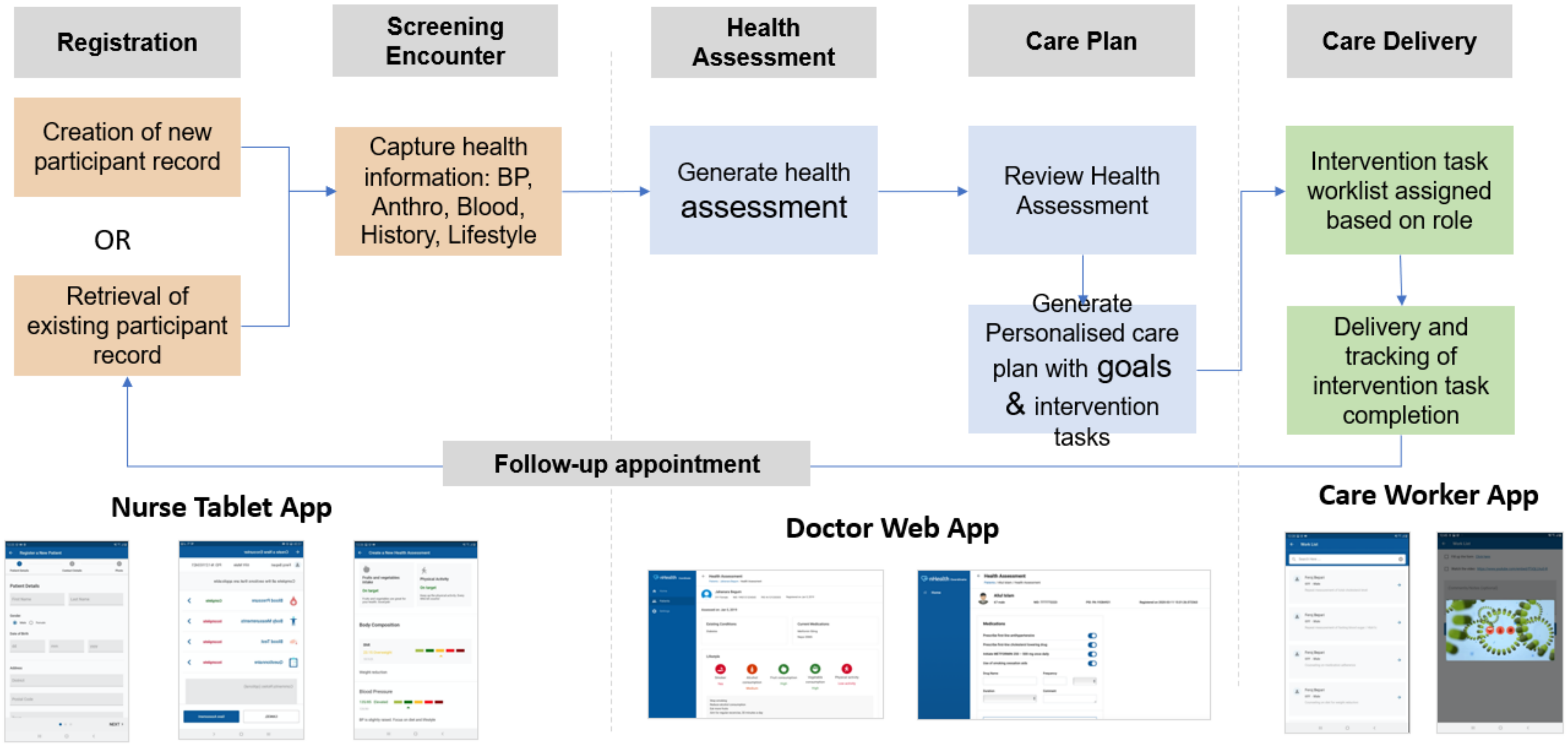

For peer review only - http://bmjopen.bmj.com/site/about/guidelines.xhtml

Supplementary Figure C. User interfaces for (A) registration, (B) patient record, (C) worklist, (D) referral, (E) care plan action, (F) counselling support

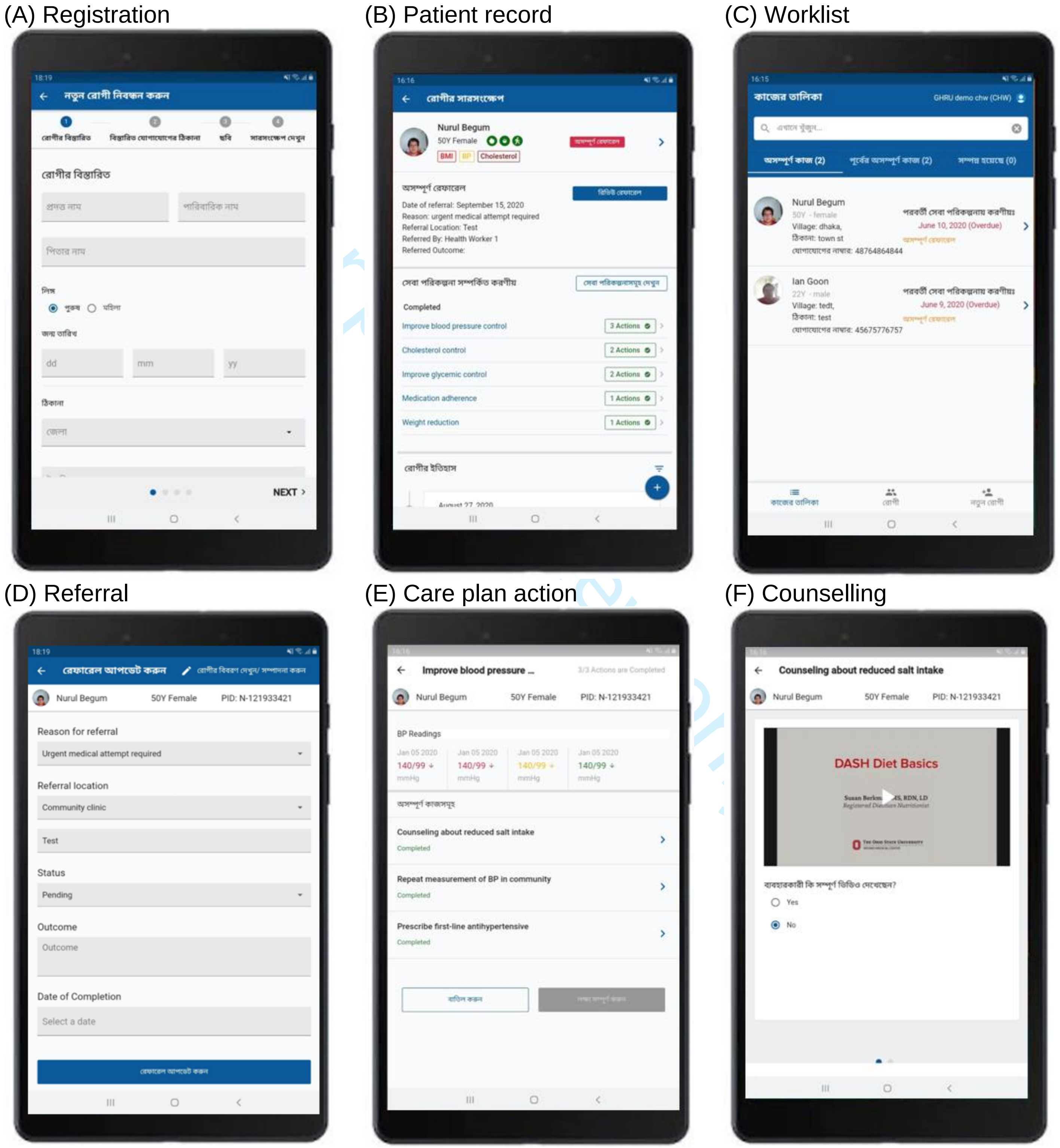

Supplementary Figure D. Central administration dashboard

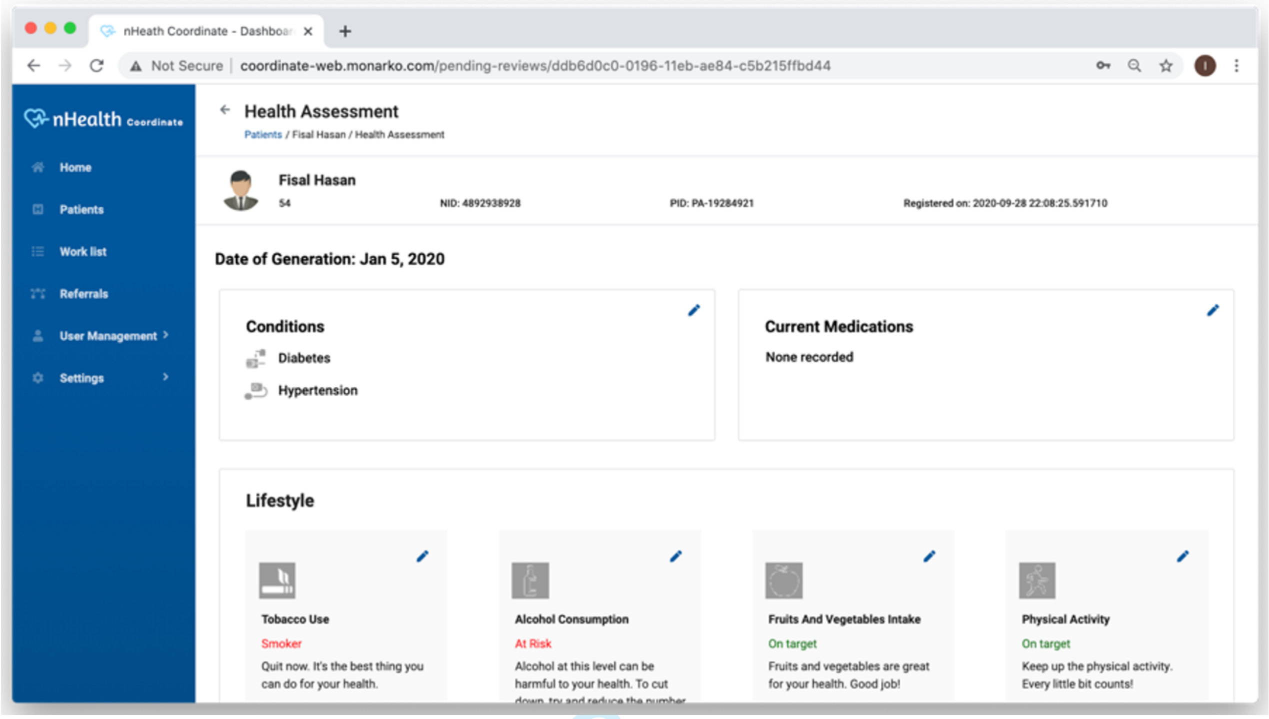

For peer review only - http://bmjopen.bmj.com/site/about/guidelines.xhtml

Supplementary Figure E. Consort diagram of the numbers of patients enrolled into care

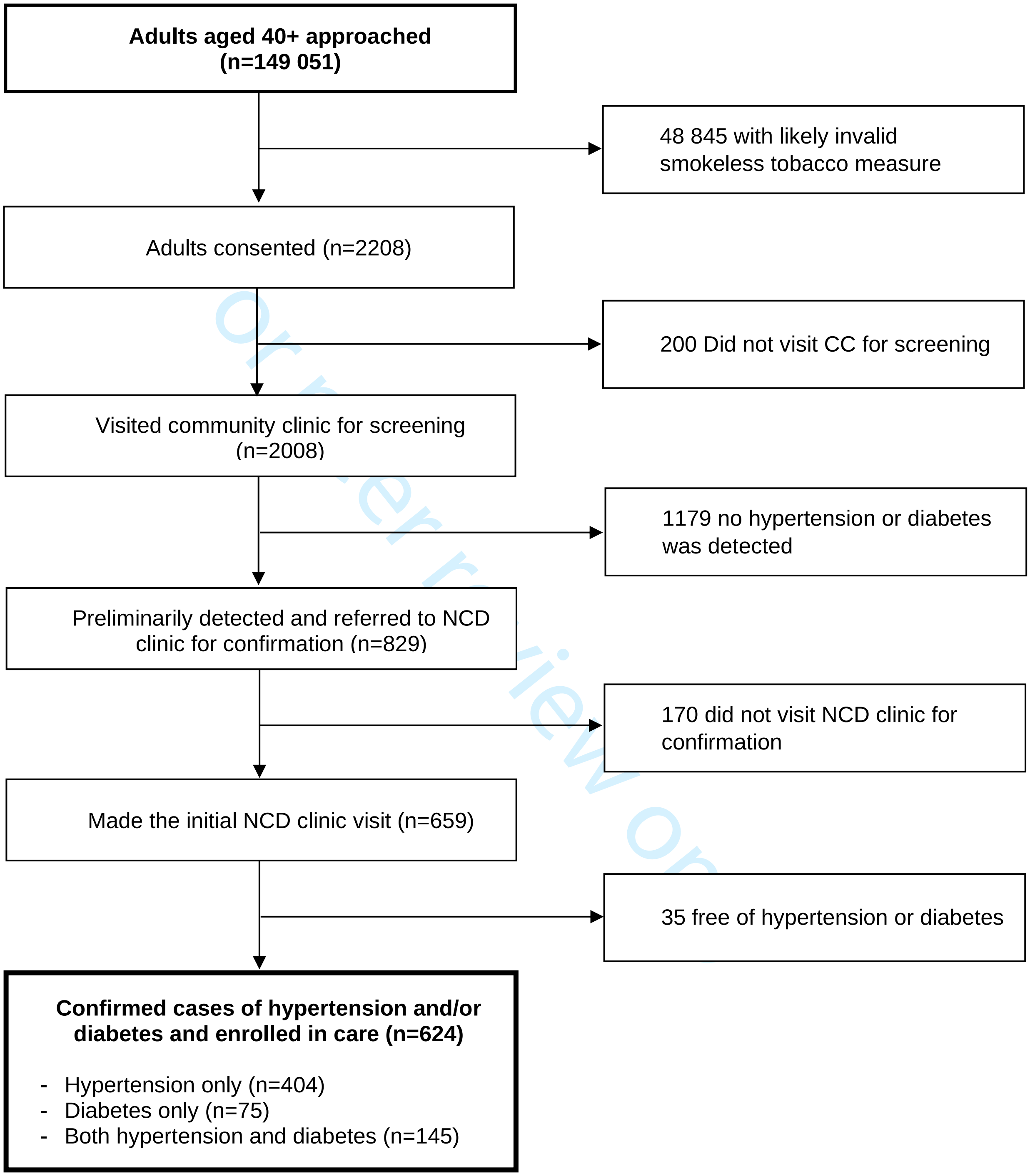

For peer review only - http://bmjopen.bmj.com/site/about/guidelines.xhtml

Supplementary Figure F. Change in mean blood pressure from baseline to follow-up visits *

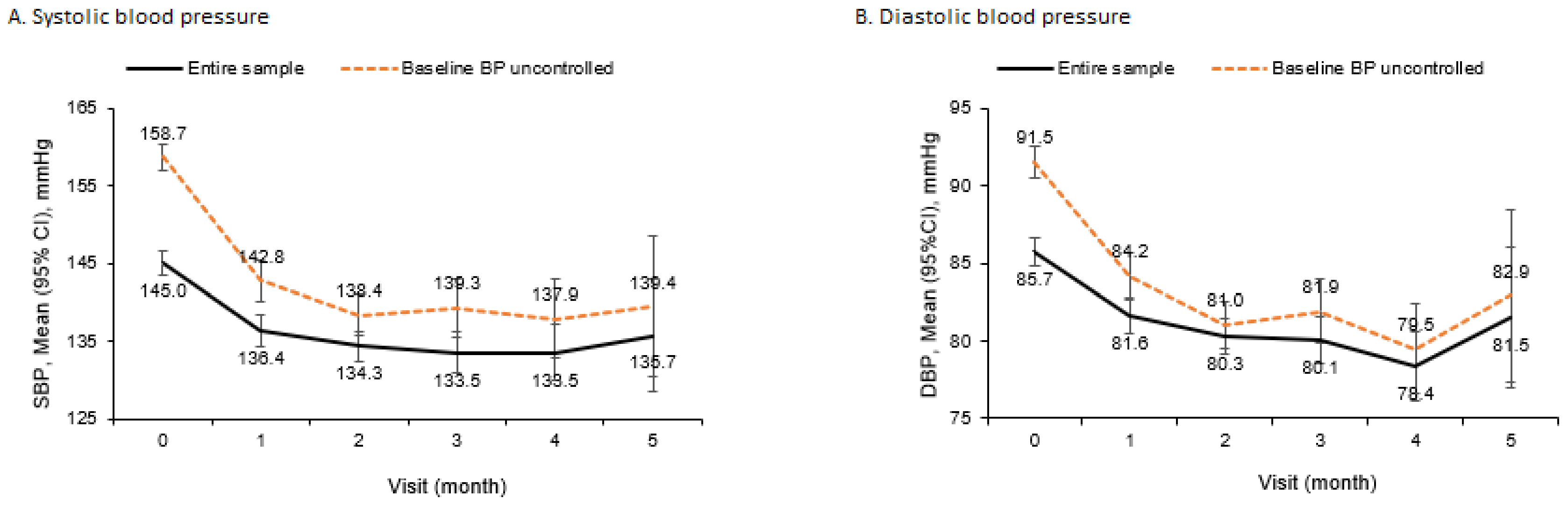

Entire sample	549	314	206	135	68	20
Uncontrolled	331	193	121	79	43	15

Entire sample	549	314	206	135	68	20
Uncontrolled	220	146	101	71	36	17

* BP, blood pressure; SBP, systolic blood pressure; DBP, diastolic blood pressure
Results adjusted age and sex.
Uncontrolled blood pressure at baseline was defined as systolic blood pressure ≥140 mmHg or diastolic blood pressure ≥90 mmHg.

For peer review only - http://bmjopen.bmj.com/site/about/guidelines.xhtml

Supplementary Figure G. Number of patient-visits per month occurred in NCD clinic and community clinic by types of tasks

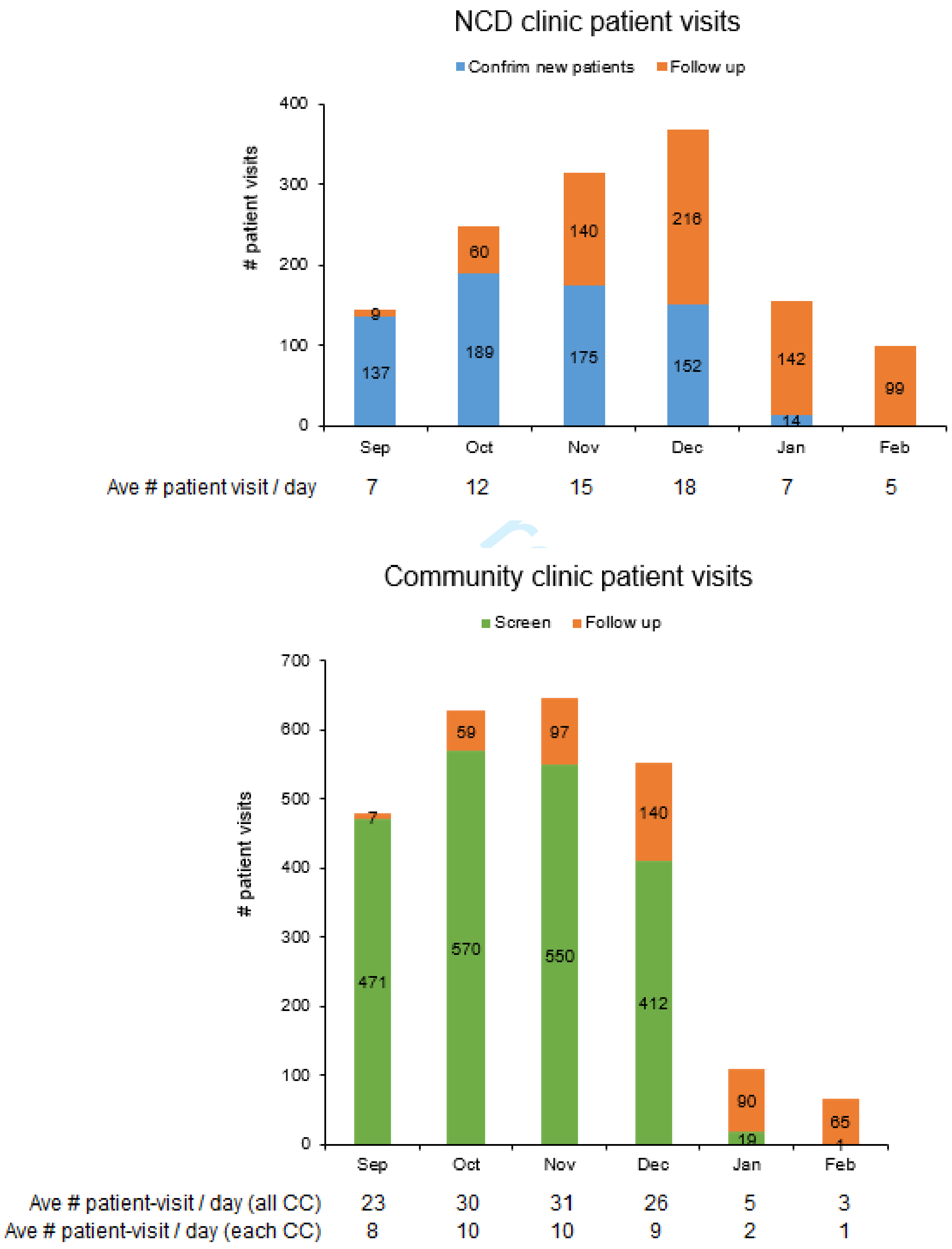

Supplementary Table A. Change in mean blood pressure from baseline to follow-up visits

Visit *	Systolic blood pressure		Diastolic blood pressure	
	Entire sample	Baseline uncontrolled cases †	Entire sample	Baseline uncontrolled cases †
1 month	-8.0 (-10.2, -5.9)	-15.2 (-17.8, -12.5)	-3.9 (-5.0, -2.7)	-7.2 (-8.7, -5.8)
2 months	-9.7 (-12.2, -7.3)	-19.7 (-22.7, -16.8)	-4.9 (-6.3, -3.6)	-10.2 (-11.9, -8.5)
3 months	-10.6 (-13.6, -7.2)	-18.8 (-22.8, -14.8)	-5.3 (-6.9, -3.6)	-9.5 (-11.7, -7.3)
4 months	-10.6 (-14.4, -6.8)	-19.9 (-24.8, -15.0)	-7.0 (-9.3, -4.6)	-11.8 (-14.7, -8.8)
5 months	-8.5 (-16.0, -0.8)	-18.3 (-27.5, -9.1)	-3.9 (-8.5, 0.8)	-8.3 (-14.1, -2.5)
Constant	126.7 (119.5, 134.1)	160.5 (150.7, 170.4)	77.2 (72.5, 81.8)	93.4 (86.5, 100.2)
N	549	331	549	331

* All models adjusted for age (40-49, 50-59, 60-69, 70-100), sex (male, female), education (no formal education, primary or lower, secondary or higher), religion (Islam, Hindu), BMI category (under, normal, overweight or obese), community clinics (Khorakhai, Manmathapur, Jahanabad).
† Baseline uncontrolled cases were defined as systolic blood pressure ≥ 140 mmHg or diastolic blood pressure ≥ 90 mmHg

For peer review only - http://bmjopen.bmj.com/site/about/guidelines.xhtml
For peer review only

Supplementary Table B. Factors associated with loss to follow-up

	LFU in the first visit	LFU over entire period
**Predictor**	IRR‡ (95% CI)	IRR‡ (95% CI)
Community clinic		
Khorakhai	1.00	1.00
Jahanabad	1.45 (1.02, 2.04)	1.21 (0.84, 1.74)
Manmathapur	1.58 (1.13, 2.22)	1.70 (1.22, 2.38)
Chronic conditions		
Both	1.00	1.00
Hypertension only	1.89 (1.31, 2.72)	1.85 (1.27, 2.70)
Diabetes only	1.27 (0.76, 2.14)	1.60 (0.96, 2.66)
Hypertension condition		
Known case, uncontrolled	1.00	1.00
Known case, controlled	1.42 (0.98, 2.06)	1.55 (1.07, 2.26)
New case	1.70 (1.20, 2.42)	1.48 (1.01, 2.15)
Religion		
Hindu	1.00	1.00
Islam	0.45 (0.32, 0.63)	0.85 (0.54, 1.32)

LFU, loss to follow up; IRR, incident rate ratio
Loss to follow-up over the entire study period is defined as no consultation recorded for two months or more.
IRRs derived from separate discrete time Poisson models controlling for age and sex, a logarithm exposure time were incorporated; only variables showing statistically significant associations were presented.

For peer review only - http://bmjopen.bmj.com/site/about/guidelines.xhtml

Supplementary Table C. Change in mean blood pressure from baseline to follow-up visits - sensitivity check for attrition and composition [*]

Visit	Original results	IPW weighting [‡]	Restricting to patients with 3+ follow-up visits
**Uncontrolled blood pressure** [§]			
1 month	0.63 (0.55, 0.72)	0.65 (0.57, 0.75)	0.63 (0.51, 0.78)
2 months	0.48 (0.39, 0.60)	0.49 (0.39, 0.61)	0.48 (0.36, 0.64)
3 months	0.44 (0.33, 0.57)	0.43 (0.32, 0.57)	0.44 (0.33, 0.59)
4 months	0.49 (0.35, 0.69)	0.48 (0.34, 0.68)	0.50 (0.35, 0.71)
5 months	0.64 (0.32, 1.25)	0.60 (0.29, 1.24)	0.65 (0.33, 1.27)
N	549	549	135
**Uncontrolled blood glucose** [¶]			
1 month	0.69 (0.58, 0.82)	0.71 (0.59, 0.85)	0.63 (0.49, 0.80)
2 months	0.66 (0.54, 0.81)	0.67 (0.53, 0.83)	0.59 (0.45, 0.77)
3 months	0.60 (0.45, 0.78)	0.62 (0.47, 0.82)	0.55 (0.41, 0.72)
4 months	0.54 (0.36, 0.80)	0.56 (0.37, 0.85)	0.47 (0.30, 0.72)
5 months	0.44 (0.20, 0.96)	0.42 (0.18, 0.95)	0.40 (0.18, 0.89)
N	220	220	71

[*] All models adjusted for age (40-49, 50-59, 60-69, 70-100), sex (male, female), education (no formal education, primary or lower, secondary or higher), religion (Islam, Hindu), BMI category (under, normal, overweight or obese), community clinics (Khorakhai, Manmathapur, Jahanabad).

[‡] The inverse probability weighting approach weighted the analysis by the inverse of the predicted probability of dropping out from the study. A conditional logit model with the same set of covariates described above were used to predict the probabilities.

[§] Uncontrolled blood pressure was defined as systolic blood pressure ≥ 140 mmHg or diastolic blood pressure ≥ 90 mmHg

[¶] Uncontrolled blood glucose was defined as fasting plasma glucose ≥ 7.0 mmol/L or random plasma glucose ≥ 11.1 mmol/L

only

For peer review only - http://bmjopen.bmj.com/site/about/guidelines.xhtml

Reporting checklist for quality improvement in health care.

Based on the SQUIRE guidelines.

Instructions to authors

Complete this checklist by entering the page numbers from your manuscript where readers will find each of the items listed below.

Your article may not currently address all the items on the checklist. Please modify your text to include the missing information. If you are certain that an item does not apply, please write "n/a" and provide a short explanation.

Upload your completed checklist as an extra file when you submit to a journal.

In your methods section, say that you used the SQUIREreporting guidelines, and cite them as:

Ogrinc G, Davies L, Goodman D, Batalden P, Davidoff F, Stevens D. SQUIRE 2.0 (Standards for QUality Improvement Reporting Excellence): revised publication guidelines from a detailed consensus process

		Reporting Item	Page Number
**Title**			
	#1	Indicate that the manuscript concerns an initiative to improve healthcare (broadly defined to include the quality, safety, effectiveness, patientcenteredness, timeliness, cost, efficiency,	1

For peer review only

		and equity of healthcare)	
**Abstract**			
	#02a	Provide adequate information to aid in searching and indexing	3
	#02b	Summarize all key information from various sections of the text using the abstract format of the intended publication or a structured summary such as: background, local problem, methods, interventions, results, conclusions	3
**Introduction**			
Problem description	#3	Nature and significance of the local problem	6,7
Available knowledge	#4	Summary of what is currently known about the problem, including relevant previous studies	6,7
Rationale	#5	Informal or formal frameworks, models, concepts, and / or theories used to explain the problem, any reasons or assumptions that were used to develop the intervention(s), and reasons why the intervention(s) was expected to work	6,7
Specific aims	#6	Purpose of the project and of this report	8
**Methods**			
Context	#7	Contextual elements considered important at the outset of introducing the intervention(s)	8,9
Intervention(s)	#08a	Description of the intervention(s) in sufficient detail that others could reproduce it	10-13

For peer review only

Intervention(s)	#08b	Specifics of the team involved in the work	10-13
Study of the Intervention(s)	#09a	Approach chosen for assessing the impact of the intervention(s)	14
Study of the Intervention(s)	#09b	Approach used to establish whether the observed outcomes were due to the intervention(s)	15
Measures	#10a	Measures chosen for studying processes and outcomes of the intervention(s), including rationale for choosing them, their operational definitions, and their validity and reliability	14
Measures	#10b	Description of the approach to the ongoing assessment of contextual elements that contributed to the success, failure, efficiency, and cost	14,15
Measures	#10c	Methods employed for assessing completeness and accuracy of data	14,15
Analysis	#11a	Qualitative and quantitative methods used to draw inferences from the data	15,16
Analysis	#11b	Methods for understanding variation within the data, including the effects of time as a variable	15,16
Ethical considerations	#12	Ethical aspects of implementing and studying the intervention(s) and how they were addressed, including, but not limited to, formal ethics review and potential conflict(s) of interest	14
**Results**			
	#13a	Initial steps of the intervention(s) and their evolution over time	16,17

For peer review only

		(e.g., time-line diagram, flow chart, or table), including modifications made to the intervention during the project	
	#13b	Details of the process measures and outcome	18
	#13c	Contextual elements that interacted with the intervention(s)	18,19
	#13d	Observed associations between outcomes, interventions, and relevant contextual elements	17,18
	#13e	Unintended consequences such as unexpected benefits, problems, failures, or costs associated with the intervention(s).	18,19
	#13f	Details about missing data	17
**Discussion**			
Summary	#14a	Key findings, including relevance to the rationale and specific aims	19
Summary	#14b	Particular strengths of the project	21
Interpretation	#15a	Nature of the association between the intervention(s) and the outcomes	19-21
Interpretation	#15b	Comparison of results with findings from other publications	19-21
Interpretation	#15c	Impact of the project on people and systems	19-21
Interpretation	#15d	Reasons for any differences between observed and anticipated outcomes, including the influence of context	19-21
Interpretation	#15e	Costs and strategic trade-offs, including opportunity costs	19-21
Limitations	#16a	Limits to the generalizability of the work	21

For peer review only

Limitations	#16b	Factors that might have limited internal validity such as confounding, bias, or imprecision in the design, methods, measurement, or analysis	21
Limitations	#16c	Efforts made to minimize and adjust for limitations	21
Conclusion	#17a	Usefulness of the work	22
Conclusion	#17b	Sustainability	22
Conclusion	#17c	Potential for spread to other contexts	22
Conclusion	#17d	Implications for practice and for further study in the field	22
Conclusion	#17e	Suggested next steps	22
**Other information**			
Funding	#18	Sources of funding that supported this work. Role, if any, of the funding organization in the design, implementation, interpretation, and reporting	2

None The SQUIRE 2.0 checklist is distributed under the terms of the Creative Commons Attribution License CC BY-NC 4.0. This checklist can be completed online using https://www.goodreports.org/, a tool made by the EQUATOR Network in collaboration with Penelope.ai

For peer review only
